# Taxation of veterinary antibiotics to reduce antimicrobial resistance

**DOI:** 10.1016/j.onehlt.2023.100650

**Published:** 2023-11-07

**Authors:** Alex L.K. Morgan, Dominic Moran, Thomas P. Van Boeckel

**Affiliations:** a Department of Environmental Systems Science, ETH Zürich, Zürich, Switzerland; b Global Academy of Agriculture and Food Systems, The Royal (Dick) School, of Veterinary Studies, The Roslin Institute, Easter Bush Campus, Midlothian, United Kingdom; c One Health Trust, Bangalore, India

**Keywords:** Antimicrobial resistance, Taxation, Mathematical model, Intervention, One health, Food-producing animals

## Abstract

Routine usage of antibiotics for animal health is a key driver of antimicrobial resistance (AMR) in food-producing animals. Taxation is a possible approach to incentivise appropriate antibiotic usage in food-producing animals. Taxation can be applied flatly across all antibiotic classes, targeted to single antibiotic classes, or scaled based on resistance in each class, so called “differential” taxation. However, quantifying the potential impact of taxation is challenging, due to the nonlinear and unintuitive response of AMR dynamics to interventions and changes in antibiotic usage caused by alterations in price. We combine epidemiological models with price elasticities of demand for veterinary antibiotics, to compare the potential benefits of taxation schemes with currently implemented bans on antibiotic usage.

Taxation strategies had effects comparable to bans on antibiotic usage in food-producing animals to reduce average resistance prevalence and prevent increases in overall infection. Taxation could also maximise the average number of antibiotics with a resistance prevalence of under 25% and potentially generate annual global revenues of ∼1 billion US$ under a 50% taxation to current prices of food-producing animal antibiotics. Differential taxation was also able to maintain a high availability of antibiotics over time compared to single and flat taxation strategies, while also having the lowest rates of intervention failure and highest potential revenue across all taxation strategies. These findings suggest that taxation should be further explored as a tool to combat the ongoing AMR crisis.

## Highlights

• Taxation can be as effective as bans on antibiotic usage in food-producing animals to reduce antimicrobial resistance.
• Taxation can generate ∼1 billion USD under a 50% increase in antibiotic price for food-producing animals.
• Economic interventions should be explored as a viable alternative to current bans on antibiotic usage in food-producing animals.

## 1 Introduction

Antimicrobial usage is a key contributor to rising antimicrobial resistance (AMR) in food-producing animals and human populations [1]. In particular, the usage of clinically important antimicrobials in food-producing animals has been linked to AMR in agricultural settings, which can spread to humans through foodborne, direct, or environmental transmission [[2], [3], [4], [5], [6]]. With an estimated 73% of all antimicrobials worldwide used in food-producing animals, an increased focus has been placed on controlling usage of veterinary antimicrobials to help preserve the efficacy of therapeutics for use in agriculture and human populations [7].

Interventions to target antimicrobial usage include bans or regulations on the prophylactic usage of antibiotics in agriculture. Such interventions were initiated in the EU in the 1990s, and with current WHO guidelines recommending the restriction of antimicrobials in growth promotion roles [[8], [9], [10]]. Bans on individual compounds and mode of application of veterinary antibiotics have also been successful, with literature suggesting reductions to resistance in clinically important *Enterococcus* spp. in broiler chickens and cattle. [4,11,12].

Market based instruments (MBIs) are an alternative to bans on antibiotic usage and include interventions such as taxation. MBIs have the benefit of allowing flexible compliance compared to bans, reducing the selective pressure for AMR by disincentivising usage, and generating income [7,[13], [14], [15]]. To date, antibiotic taxation schemes have only been implemented in a limited number of countries. Examples include a 2014 Belgian tax on veterinary antibiotics, based on the quantity of active ingredient sold and a Danish tax on critical care antibiotics [16,17]. This type of differentiated tax was originally implemented in pesticides and have also been effective, resulting in more conservative usage of toxic pesticides [[18], [19], [20]]. If applied in the context of antibiotics, these types of differentiated taxes could be used to reduce the usage/resistance of antibiotics with a high level of clinical importance.

Understanding and quantifying the impact of interventions, such as taxation, is complex due to the unintuitive nature of AMR dynamics [21]. However, epidemiological models can be used to better understand the non-linear dynamics of AMR and the impact of interventions [22]. Previous attempts to quantify the impact of taxation on antibiotic usage and AMR have extrapolated from economic analyses using metrics such as price elasticity of demand (PED) [7]. However, no model to date has explored how the price elasticities of antibiotics used in food-producing animals can be linked to the epidemiology of AMR, and how the non-linear dynamics of AMR might respond to the introduction of taxation.

In this work, synthetic price elasticities of demand are combined with an epidemiological model. We compare the effectiveness of different taxation strategies on the altering the price and usage of antibiotics, and subsequently on the prevalence of AMR, the number of overall infections caused by both drug-sensitive and resistant pathogens, and the maintenance of a portfolio of available antibiotics under a 25% resistance threshold. We explore multiple taxation strategies: flat taxation applied uniformly across all antibiotic classes, targeting single antibiotic classes and scaling taxation for each antibiotic class proportional to their relative level of resistance in food-producing animals (differential taxation). The effectiveness of taxation is compared with bans on usage of specific classes of antibiotics.

## 2 Materials and methods

### 2.1 Epidemiological model

**Fig. 1:**
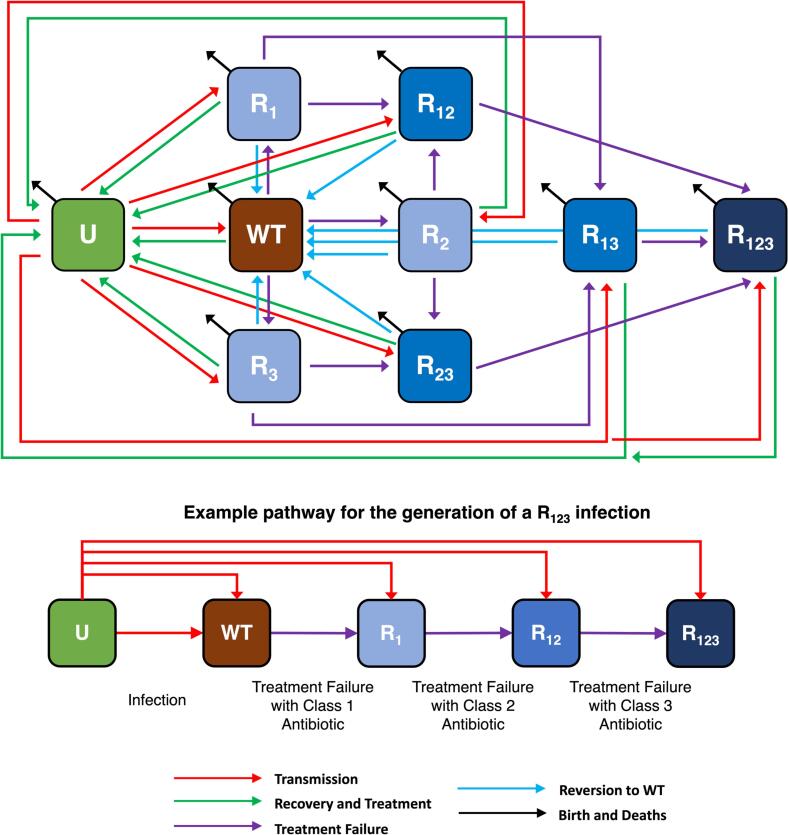
Transmission and generation of multi-drug resistance (MDR) in a population of food-producing animals exposed to three hypothetical antibiotic classes. Uninfected (U), Wild-Type (WT), Resistant to Class 1 (R_1_), Resistant to Class 2 (R_2_), Resistant to Class 3 (R_3_), Resistant to Class 1 + 2 (R_12_), Resistant to Class 1 + 3 (R_13_), Resistant to Class 2 + 3 (R_23_) and Resistant to Class 1 + 2 + 3 (R_123_). The full set of ordinary differential equations relating to the model can be found in the *Supplementary Material*.

The proportion of food-producing animals uninfected (U), infectious with wild-type bacteria (WT), bacteria resistant to one drug (R_1_, R_2_, R_3_), two drugs (R_12_, R_13_, R_23_) or three drugs (R_123_) was modelled using a deterministic model (Fig. 1). Uninfected food-producing animals are colonised/infected at a per-capita transmission rate (β) and enter the uninfecteds through birth rate λ, and leave through mortality at the same rate to ensure a constant population size. Transmission-related fitness costs of resistance were also modelled, reducing the efficacy of transmission for individuals infected with antibiotic-resistant strains (c_1_, c_2_, c_3_, c_12_, c_13_, c_23_, c_123_). Fitness costs had the impact of driving heterogeneity in transmission across the three modelled drug resistance classes. Recovery from WT, singly resistant, doubly resistant and triply resistant infections were modelled as rates r_WT_, r_r_, r_rr_, r_rrr_ respectively and were dependent on the extent of antibiotic usage of the three modelled antibiotic classes.

Antibiotic usage was described as the proportion of food-producing animals exposed to one of three antibiotic classes (σ_1_, σ_2_, σ_3_). The impact of antibiotic exposure is two-fold: 1) a therapeutic effect resulting in an increased rate of recovery (r_t_), with probability 1-ρ [23] and 2) a probability of treatment failure resulting in gain of resistance (ρ). Treatment failure occurs at rate η_wt_, η_rr_ and η_rrr_ for individuals colonised with singly, doubly or triply resistant strains.

Treatment failure and generation of resistance was assumed to occur in a stepwise fashion and can be considered analogous to antibiotic usage acting as a selection pressure, allowing for the proliferation of an implicitly assumed, small, non-infectious quantity of resistant bacteria. Reversion from resistance back to WT infection was assumed to only occur in infected food-producing animals not exposed to antibiotics (1-σ_1_ + σ_2_ + σ_3_), with a lack of antibiotic pressure allowing for fitter WT bacteria to outcompete resistant strains [24].

### 2.2 Baseline model fitting

The three antibiotic classes were fitted as a baseline scenario using an approximate Bayesian computation sequential Monte-Carlo (ABC-SMC) to ensure high (40%), medium (25%) and low (10%) levels of resistance respectively for the three antibiotics modelled (*Supplementary Material*) [25]. The aggregated dynamics of resistance to the three antibiotic classes were tracked in the model, defined as R_1_, R_2_ and R_3_, and defined as the sum of the population resistant to each antibiotic class (i.e. R_1_ = R_1_ + R_12_ + R_13_ + R_123_).

### 2.3 Taxation

Taxation was described as % alterations in the price of each antibiotic class, adapting the price elasticity of demand (PED) formula %Change in Usage=PED×%Change in Price, to model a % change in the proportion of the population using a specific antibiotic (σ_1_, σ_2_, σ_3_).

A synthetic demand matrix described the effect of PEDs, with the diagonal and non-diagonal elements representing the own-PED for each antibiotic and cross-PED respectively. The cross-PED can be interpreted as the impact of taxation of antibiotic classes on the usage of other antibiotics. We use a case study assigning first (class 1), second (class 2) and last-line antibiotic (class 3) identities to the three antibiotic classes, with the range of elasticity values obtained from an estimate of the distribution of own price elasticities of demand of veterinary antibiotics (mean values of −1.75 to −0.68) [7].

(1.1) $$\mathrm{PED}=\left[\begin{array}{ccc} -1.5 & 1 & 0.5\\ 0.5 & -1.25 & 0.75\\ 0.25 & 0.5 & -1 \end{array}\right]$$

Antibiotics with “first-line” identities were assumed to have higher elasticities due to the availability of alternative antibiotics, and that the elasticities of successive antibiotic classes decrease due to having fewer alternatives. The cross-PED was higher for antibiotics next in “priority” to the taxed antibiotic (e.g. antibiotic class 1 to 2), with an assumption that veterinarians are more likely to switch to the antibiotic next in therapeutic “importance”.

### 2.4 Interventions

Three taxation strategies were modelled, fixed at a baseline rate of 50% (increase in price of 50%) introduced after the prevalence of resistance reaches an endemic equilibrium (*t* = 8.22 years or 3000 days): 1) Taxation applied across all antibiotics (Flat Tax; FT), 2) single taxation to highest, medium and lowest resistance antibiotic classes (ST) or 3) a differential taxation scheme (DT).

The principle of differential taxation is that taxation is scaled at intervals or “rounds” (every 3 years at baseline) relative to the prevalence of resistance in the taxed antibiotic class (Fig. S1–2). As an example, assuming an 0.8, 0.5 and 0.25 resistance prevalence across the three antibiotic classes, changes to the taxation rate would be: 1.6× for class 1 (0.8/0.5), 1× for class 2 (0.5/0.5), and 0.5× for class 3 (0.25/0.5).

Taxation strategies were compared against bans on antibiotic usage to identify if similar impacts can be achieved when compared to the *status quo* of antibiotic usage interventions, in highest, medium, or lowest resistance antibiotic classes. Note that all non-differential taxation interventions were constant throughout the intervention period.

### 2.5 Performance criteria

To compare the efficacy of the intervention strategies, we considered three criteria relevant to AMR in food-producing animals: 1) maximising decreases to average resistance across all antibiotics, 2) preventing increases to overall infections and animal disease due to a loss in antibiotic pressure (due to changes in usage) and 3) maximising the average number of available antibiotics (defined at a baseline prevalence of resistance <25%) over the course of the modelled intervention period (*t* = 10,000 days or 20 years). The first two criteria were calculated relative to the change in overall antibiotic usage (Eq. 1.2–1.3).

(1.2) $$\mathrm{Change in Resistance}=\frac{\int_{t=3000}^{t=10300}{\mathrm{AvgRes}}_{\mathrm{Base}}-{\mathrm{AvgRes}}_{\mathrm{Int}}\mathrm{dt}}{\int_{t=3000}^{t=10300}{\mathrm{Usage}}_{\mathrm{Base}}-{\mathrm{Usage}}_{\mathrm{Int}}\;\mathrm{dt}}$$

(1.3) $$\mathrm{Change in Infections}=\frac{\int_{t=3000}^{t=10300}{\mathrm{TotInf}}_{\mathrm{Int}}-{\mathrm{TotInf}}_{\mathrm{Base}}\;\mathrm{dt}}{\int_{t=3000}^{t=10300}{\mathrm{Usage}}_{\mathrm{Base}}-{\mathrm{Usage}}_{\mathrm{Int}}\;\mathrm{dt}}$$

These relative performance criteria identify “efficient” interventions that have the greatest reductions in AMR for a given change in antibiotic usage (standardised at total antibiotic curtailment; σ_1_ + σ_2_ + σ_3_ = 1 to 0). These relative criteria also enable direct comparisons between interventions.

### 2.6 Uncertainty analysis

The proportion of times that each intervention was the “best-performing” strategy for each criterion was identified by running the interventions on *n* = 1000 biologically plausible parameter sets generated through Monte-Carlo sampling of parameters. Parameter ranges were centred around the baseline values obtained through ABC-SMC model fitting (Table 1).

**Table 1:** Parameter ranges used for uncertainty analysis.

Parameter	Description	Parameter Range	Baseline Values	References
*λ*	Birth/death rate in food-producing animals	7300^−1^ - 36.5^−1^ days	365^−1^ days	[26]
*β*	Rate of transmission between infected and uninfected food-producing animals	0–10	4.919	Fitted
*σ*_*x*_	Proportion of the population using class X antibiotic	0–1	0.25	Assumed
*r*_*X*_	Rate of recovery for food-producing animals with X-type infection	365^−1^ - 1^−1^ days	r_wt_ = 1/12 r_r_ = 1/10 r_rr_ = 1/8 r_rrr_ = 1/8 r_t_ = 1/7	Assumed
*η*_*wr*_^1^	Rate of conversion from wild-type to antibiotic-resistant infection	0.15–10	1.531	Fitted
*η*_*rw*_^1^	Rate of reversion from antibiotic-resistant to wild-type infection	0.006–0.6	0.062	Fitted
*η*_*rr*_^1^	Rate of conversion from singly resistant to doubly resistant infection	0.009–0.9	0.094	Fitted
*η*_*rrr*_^1^	Rate of conversion from doubly resistant to triply resistant infection	0.009–0.9	0.094	Fitted
*c*_*X*_^1^	Transmission-related fitness cost related to infection with resistance to class X antibiotic	0.5–1	c_1_ = 0.96 c_2_ = 0.90 c_3_ = 0.66 c_12_ = 0.63 c_13_ = 0.60 c_23_ = 0.59 c_123_ = 0.54	Fitted
*ρ*	Probability of treatment failure upon exposure to effective antibiotic	0–1	0.05	Assumed
*Base Tax*	Baseline taxation rate	0–1	0.5	Assumed

1 Parameters were subject to the following inequality constraints c_1_ ≈ c_2_ ≈ c_3_ > c_12_ ≈ c_13_ ≈ c_23_ > c_123_, r_wt_ > r_r_ > r_rr_ > r_rrr_ > r_t_, $\sum_{x=1}^{n}\sigma_{x}\leq 1$ (with *n* antibiotic classes) and η_WR_ > η_RW._

This metric was weighted by the probability of intervention failure, defined as an increase of both usage and resistance resulting from the intervention (*Supplementary Material*). The distribution of effect sizes for each intervention across each criterion was also assessed.

### 2.7 Scenario analyses

Scenario analyses were conducted to assess the robustness of the model to changes in model assumptions. This included alterations to the baseline taxation rate (10%, 25%, 75%, 90%), cross-elasticity of demand (0.4 or 0.6), varying the entire PED matrix (sampling the cross-PED from 0 to 1 and own-PED from −2 to 0), varying the threshold for an “effective antibiotic” (5%, 10%, 35%), the number of modelled antibiotics (two or four) and modelling specific food-producing animals (cattle *λ* = 365^−1^ days; broiler chicken *λ* = 42^−1^ days) and production systems (extensive *β* = 0.5; intensive *β* = 50).

### 2.8 Estimated yearly revenue from taxation

To identify the global annual revenue from the taxation of veterinary antibiotics used in food-producing animals (US$) for each taxation strategy, information was combined on: 1) model output on the % increases in price (taxation rate) over the 20-year intervention period for each intervention, and 2) the yearly market value of different antibiotic classes in food-producing animals.

To generate an estimate for the yearly market value of veterinary antibiotics, information on the sales and price of different antibiotic classes in food-producing animals was combined for each country globally. Details of the included antibiotic classes can be found in the *Supplementary Material*. Antibiotic price data from the US and China were used as proxies to represent high-income countries (HICs) and low/middle-income countries (LMICs) respectively. Pricing data was identified from online retailers and harmonised to US Dollars (US$) using a conversion rate from Chinese Yuan (*Supplementary Material*) [27,28]. The proxied price for HICs and LMICs was then multiplied by global antibiotic sales data for food-producing animals for each antibiotic class in every HIC and LMIC [29,30]. This enabled a determination of the yearly global revenue (US$) from each antibiotic class for all HICs and LMICs. Note that the total estimated revenue across each antibiotic class was scaled to match the estimated market value for veterinary antibiotics of 2.731 billion US$ [31].

Elasticity values were next assigned to each antibiotic class based on their respective level of resistance, classifying antibiotics as one of the three antibiotics modelled (1, 2 or 3) in the synthetic PED matrix (eq. 1.1). Prevalence data on resistance for each antibiotic class in *Salmonella* spp. and *Campylobacter* spp. was obtained from the 2021 NARMS report (US) and resistancebank.org (China) [32,33]. Higher resistance antibiotic classes were assumed to correspond to less critical antibiotics (group 1) (*Supplementary Material*).

## 3 Results

### 3.1 Efficacy of taxation schemes and bans

**Fig. 2:**
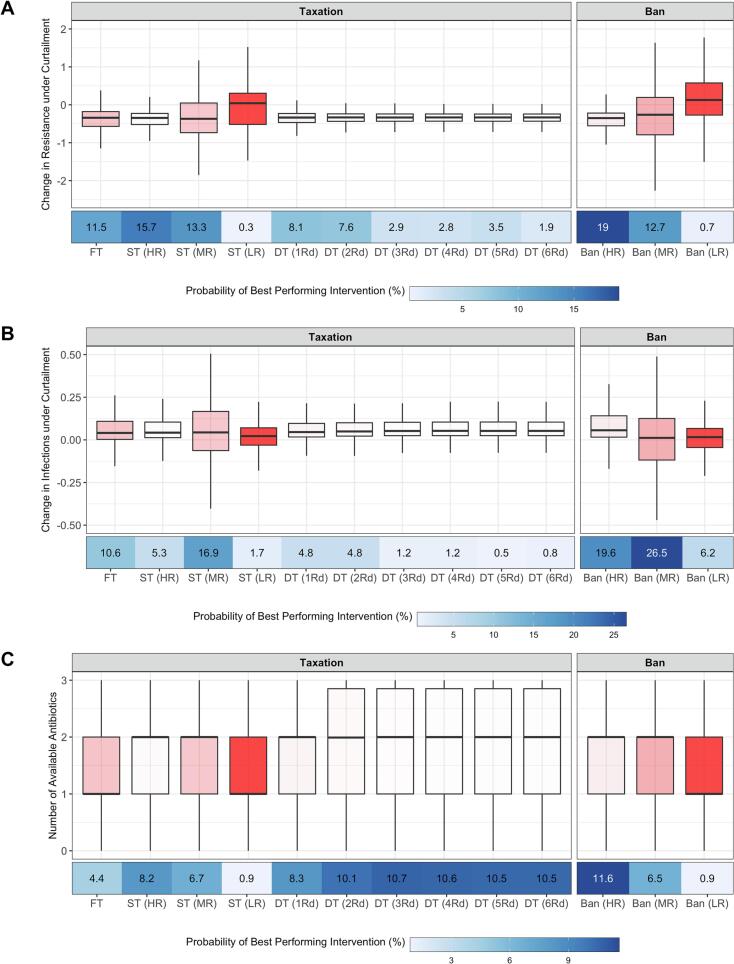
A) Changes to average resistance under total antibiotic curtailment, B) changes to overall infections under total antibiotic curtailment, C) Number of available antibiotics. FT = Flat Tax, ST = Single Tax, DT = Differential Tax, HR = High Resistance, MR = Medium Resistance and LR = Low Resistance. Multiple rounds of differential taxation were explored (1-6Rds). The intensity of box plot shading represents the proportion of runs resulting in increases to both usage and resistance, representing intervention failure (also used for weighting of intervention performance: 27.5%, 2.1%, 25.3%, 80.7%, 5.2%, 2.8%, 1%, 0.9%, 0.9%, 0.9%, 7.9%, 36% and 80.3%). Pairwise significance testing to compare distributions were included in the *Supplementary Material*.

Changes to average resistance suggest that curtailment of veterinary antibiotic usage in food-producing animals from full usage (σ_1_ + σ_2_ + σ_3_ = 1) to no usage (σ_1_ + σ_2_ + σ_3_ = 0), would result in a median reduction of the proportion of resistant infections in food-producing animals ranging between 0.26 (Ban MR; 95% CI: −2.22, 1.63) to 0.37 (ST LR; 95% CI: −1.85, 1.17) (Fig. 2A) across the interventions. Increases in infection ranged between 0.01 (Ban MR; 95% CI: −0.46, 0.49) to 0.06 (Ban HR 95% CI: −0.17, 0.33) (Fig. 2B). Additionally, the average number of available antibiotics ranged across interventions from a median value of 1 to 2 available antibiotics (95% CI: 0, 3) (Fig. 2C). The exception to these ranges of effect sizes was with interventions to target the lowest levels of resistance (Ban LR: 0.12; 95% CI: −1.5, 1.77).

However, bans (HR and MR) on antibiotic usage were identified as the “best” performing intervention according to the performance metric to minimise average resistance (19%; 95% CI: 17%, 22%), minimise increases in overall infection (26.5%; 95% CI: 24%, 29%) and maximise the average number of available antibiotics (11.6%; 95% CI: 11%, 12%) (Fig. 2).

Differential taxation was the most similar strategy to bans to maximise the average number of available antibiotics and with the smallest range of intervention failure rates (0.9% - 5.2%). Taken together across all three criteria, this suggests that taxation strategies can perform as effectively as bans on antibiotic usage for the range of parameters explored. To explore the similarity in effect size between taxation and bans, the taxation rate (HR) was incremented from 1% to 100%. A linear change in effect size to reduce resistance was observed, plateauing to a level comparable to bans at 60% taxation (HR: -0.36; 95% CI: −0.90, 0.13) (Fig. S5–6).

The results were found to be robust when alternative performance criteria were used: 1) absolute changes in resistance/infection, and 2) % changes to resistance/infections per % decrease in usage (Fig. S7–8). However, an exception was with an improved performance of single taxation to reduce resistance in the second alternative criteria. The results were also unchanged when the hierarchy of fitness costs assumption was removed (Fig. S9).

### 3.2 Scenario analyses

**Fig. 3:**
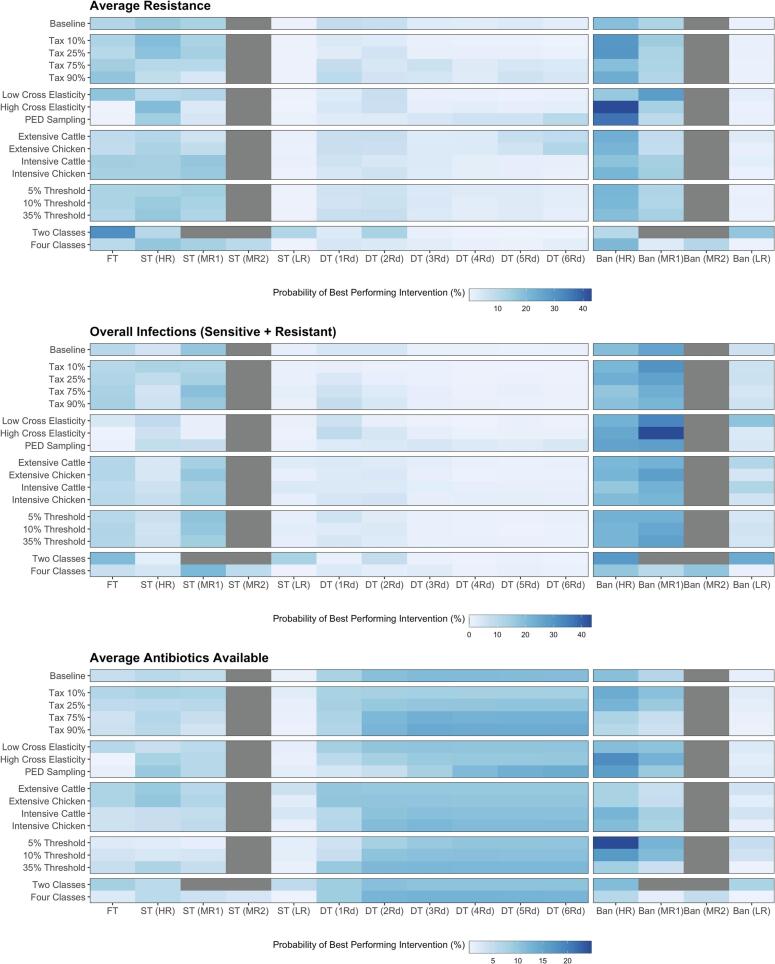
Probability that each intervention performs best to minimise average resistance, minimise increases to overall infections, and maximise the number of available antibiotics across different scenarios. FT = Flat Tax, ST = Single Tax, DT = Differential Tax, HR = High Resistance, MR1 = 2nd Lowest Resistance, MR2 = 3rd Lowest Resistance, LR = Low Resistance. The baseline for rescaling for differential taxation in the scenarios with two and four antibiotic classes was calculated as the average of the two medium resistance classes (4 classes) or the average of the only two antibiotic classes (2 classes). Note that box plots with effect sizes for all scenarios can be found in the *Supplementary Material* (Fig. S11–26).

Alternative scenarios were used to explore the robustness of best performing interventions. These scenarios related to the extent of taxation (90%, 75%, 25% and 10%), altering the PED matrix (cross PED values of 0.4 or 0.6 or varying the matrix), the number of antibiotic classes (two vs four), a case study with cattle and broiler chicken production systems (*β* = 0.5 or 50; *λ* = 1/365^−1^ or 1/42^−1^ days) and the threshold for the resistance for which an antibiotic is considered “available” (5%, 10% and 35%) (Fig. 3).

The qualitative pattern of best performing interventions was maintained across alternative scenarios, with bans on antibiotic usage maintained as the being the best performing strategy to reduce average resistance and overall infection (Fig. 3). The exception to this qualitative pattern was modelling two antibiotic classes, which increased the performance of taxation above bans on usage. Stricter thresholds for an available antibiotic, reducing the rate of taxation, varying the PED matrix and cross elasticity of demand also reduced the performance of differential taxation. The efficacy of flat taxation was also strongly impacted by alterations to the PED matrix. The use of extensive production reduced the probability of intervention failure and increased the average availability of effective antibiotics (Fig. S23–26).

### 3.3 Estimated revenue from taxation

**Fig. 4:**
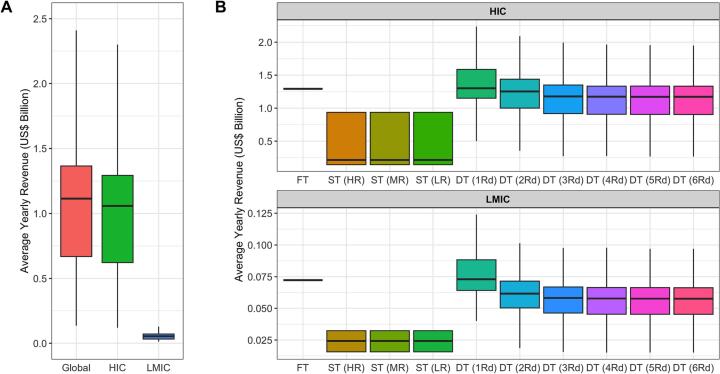
Projected revenue from taxation strategies for High-Income Countries (HICs), Lower-Middle Income Countries (LMICs). A) Aggregated median income for all countries, HICs and LMICs across all explored taxation strategies. B) Estimated revenue for each taxation strategy. FT = Flat Tax, ST = Single Tax, DT = Differential Tax, HR = High Resistance, MR = Medium Resistance, LR = Low Resistance.

The median global yearly revenue averaged across all considered taxation strategies was $1.11 billion (95% CI: 0.13, 2.4) (Fig. 4A). Differential taxation ($1.3 billion; 95% CI: 0.5, 2.23) and flat taxation strategies ($1.29 billion; 95% CI: 1.29, 1.29) generated the largest median revenue across explored taxation strategies (Fig. 4B).

The average yearly median revenue across all taxation strategies was higher in HICs ($1.06 billion; 95% CI: 0.11, 2.3) when compared to LMICs ($0.05 billion; 95% CI: 0.01, 0.12), attributable to the higher average price of antibiotics in the former case study (*Supplementary Material*). This analysis was also explored with alternative tax rates of 25% and 75%, with median global yearly revenue of $0.61 (95% CI: 0.06, 1.2) and $1.52 (95% CI: 0.18, 3.86) billion respectively (Fig. S30–31).

## 4 Discussion

Taxation strategies were found to have similar median effect sizes to outright bans on antibiotic usage in food-producing animals to maximise reductions to resistance, minimise increases in infections and maximise the average number of available antibiotics. Additionally, taxation could generate a global median annual revenue of $1.11 billion under a taxation rate of 50% that could be reinvested into antibiotic development or agricultural biosecurity. However, bans on antibiotic usage could more frequently achieve these effect sizes across the three criteria when compared to taxation for the explored parameter space.

Bans on antibiotic usage were identified as the best performing intervention across all three criteria. This can be attributable to the low variance and having the largest median effect size when compared across all modelled strategies. This adds support to the current and historical implementation of bans to control AMR in food-producing animals [34]. However, differential taxation, flat taxation and single taxation to high resistance (HR) classes also resulted in comparable variance and median effect sizes to bans, suggesting that taxation can be a plausible alternative to implemented bans on usage.

Market based interventions (MBIs) such as taxation and its near equivalent of a cap and tradeable permit scheme also have benefits over bans, allowing for flexibility in the usage of antibiotics, with bans being punitive of all users irrespective of their relative cost of abatements [15]. MBIs also generate yearly revenue, with ∼1 billion US$ estimated in this study. This should be placed into context with the estimated 1 to 2 billion US$ development cost for a novel antibiotic and the 540 billion US$ global support to agricultural producers, which could help improve biosecurity and reduce reliance on antibiotics used in food-producing animals [7,35,36]. In more budget restricted LMICs, the additional revenue could be directed for non-agricultural purposes with indirect benefits for farmers [37]. The introduction and coordination of MBIs can also be facilitated by National Action Plans (NAPs), with taxation collected by retailers and sent to respective government agencies [18,38].

Use of extensive production systems with lower transmission will also impact the efficacy of interventions, increasing the average availability of effective antibiotics and reducing intervention failure (Fig. 3). Mode of application may also impact patterns of usage, with prescription-based EU usage of antibiotics for food-producing animals likely reducing elasticity of antibiotics due to a limited access to alternatives [39]. Future understanding of the impact of economic interventions would benefit greatly from more accurate insight into the response of antibiotic usage to changing market conditions and production systems.

However, the main advantage of bans over taxation is the certainty in reductions to usage. In contrast, the efficacy of MBIs will be sensitive to uncertainty in market dynamics and some users may simply opt to pay a tax rather than altering consumption patterns [19]. There are also questions about the incidence of the burden of taxation, which will ultimately fall on the consumer, and the potential impacts on food security [40]. However, it is worth noting that given the punitive nature of bans, the harms of bans while reducing usage will be greater than for taxation. MBIs such a permit trading scheme can alternatively guarantee changes in usage through caps on usage, but carry uncertainty in the generation of revenue [15]. Future assessments of the viability of MBIs must therefore consider criteria other than the epidemiological outcomes explored by this study.

Differential taxation was identified as an effective strategy in this study, dynamically controlling resistance across the portfolio of antibiotics by promoting periods of high and low resistance [23,41,42]. The targeted nature of differential taxation is also what promotes the low level of intervention failure, preventing large compensatory increases in resistance. Differential taxation also resulted in the greatest certainty in effect size across considered interventions, which may prove to be beneficial for the risk-averse nature of agricultural policy making [43]. These unique benefits of differential taxation, combined with the similar median effect size to bans, suggests that differential taxation should be explored as a tool to ensure the availability of veterinary antibiotics.

However, the cyclical nature of differential taxation may mean antibiotics critical for food-producing animals such as glycopeptides and streptogramins undergo periods of high resistance [44]. Differential taxation will also require periodical recalibration of taxation, which might prove difficult for implementation, with literature citing stability and ease of implementation being critical for effective taxation [18,43]. Although beyond the scope of this study, future models could explore additional layers of taxation on “last-line” antibiotics, similar to contemporary Danish or Belgian schemes [16,17]. Implementation of differential taxation will require improvements to current AMR surveillance, so that taxation can be accurately calibrated based on the prevalence in each antibiotic class.

Despite HICs only making up 20.4% of global sales of veterinary antibiotics, HICs were estimated to contribute the majority of global revenue from taxation. However, the variation surrounding this estimate suggests scenarios where revenue can be twice higher than the median, or close to levels observed in LMICs. Risk-averse policy makers may opt for flat taxation, with little variation in revenue expected if this is the primary goal. The result of this analysis also introduces a series of ethical questions: is the unequal contribution from HICs fair, considering the historical usage of veterinary antibiotics and higher GDP in HICs? Additionally, what would be the implications of these taxes be on small-holding farmers in LMICs? Could a scheme be introduced to compensate farmers in LMICs for potential losses in productivity, or to implement taxation in HICs only?

Two scenarios demonstrated relatively higher rates of intervention failure: 1) high cross elasticity of demand, or 2) targeting the lowest resistance antibiotic class. Both can be attributed to the impact of compensatory increases in usage beyond what is being reduced through the intervention. Implementation of future economic interventions must avoid scenarios which lead to this increased risk of intervention failure, which has been identified in the implementation of pesticide taxation [19].

By integrating an epidemiological model with the price elasticity of demand, this study provides a first quantitative exploration into the viability and efficacy of taxation as a potential strategy to tackle AMR in food animals. We highlight taxation, as a potential tool for AMR control in agriculture, which should be explored through further modelling work. However, this must also be done in tandem with improvements to surveillance and an increased understanding of the elasticity of antibiotics for use in food animals. It is vital that we continue to explore tools and strategies that can effectively reduce AMR to tackle the ongoing AMR crisis in both food animals and in humans.

## Authors' contributions

ALKM participated in the study design, carried out model analysis and drafted the manuscript. TPVB participated in the study design and provided feedback on manuscript drafts. DM provided feedback on manuscript drafts.

## Funding

ALKM and TPVB were supported by the Swiss National Science Foundation Eccellenza Fellowship (No 181248) and the Branco Weiss Foundation. DM wishes to acknowledge UKRI funding under grant numbers BB/W018152/1, BB/T004436/1.

## Declaration of Competing Interest

All authors declare that they have no conflicts of interest.

## Data Availability

Data availability I have attached a GitHub link in the “attach data” step Github Repository (Original data)

## Appendix A Supplementary data

Supplementary material (text and figures) Image 1
