# Supplementary material for: Taxation of veterinary antibiotics to reduce antimicrobial resistance
Source: One Health. 2023 Nov 7;17:100650. doi: 10.1016/j.onehlt.2023.100650 (PMC10665208; doi:10.1016/j.onehlt.2023.100650)
Supplement: Supplementary file 1 — Supplementary material (text and figures) [file mmc1.docx]

***Supplementary Materials*** *- Quantifying the efficacy of taxation on antibiotics to control antimicrobial resistance in food animals using mathematical modelling*

*Model Equations*

$$\dot{U}= \lambda-\lambda U-\beta U\left( WT+R_{1}c_{1}+R_{2}c_{2}+R_{3}c_{3}+R_{12}c_{12}+R_{13}c_{13}+R_{23}c_{23}+R_{123}c_{123}+R_{1}c_{1}+R_{1}c_{1} \right)+r_{WT}WT\left( 1-{(\sigma}_{1}+\sigma_{2}+\sigma_{3}) \right)+r_{r}R_{1}\left( 1-\left( \sigma_{2}+\sigma_{3} \right) \right)+r_{r}R_{2}\left( 1-\left( \sigma_{1}+\sigma_{3} \right) \right)+r_{r}R_{3}\left( 1-\left( \sigma_{1}+\sigma_{2} \right) \right)+r_{rr}R_{12}\left( 1-\sigma_{3} \right)+r_{rr}R_{13}\left( 1-\sigma_{2} \right)+r_{rr}R_{23}\left( 1-\sigma_{1} \right)+r_{rrr}R_{123}+r_{t}\left( 1-\rho\right)\left( WT{(\sigma}_{1}+\sigma_{2}+\sigma_{3} \right)+R_{1}\left( \sigma_{2}+\sigma_{3} \right)+R_{2}\left( \sigma_{1}+\sigma_{3} \right)+R_{3}\left( \sigma_{1}+\sigma_{2} \right)+R_{12}\sigma_{3}+R_{13}\sigma_{2}+R_{23}\sigma_{1})$$

$$\dot{WT}=-\lambda WT+\beta UWT-r_{WT}WT\left( 1-{(\sigma}_{1}+\sigma_{2}+\sigma_{3}) \right)-r_{t}WT\left( 1-\rho\right){(\sigma}_{1}+\sigma_{2}+\sigma_{3})+\eta_{RW}(R_{1}+R_{2}+R_{3}+R_{12}+R_{13}+R_{23}+R_{123})(1-{(\sigma}_{1}+\sigma_{2}+\sigma_{3}))-\eta_{WR}WT\rho{(\sigma}_{1}+\sigma_{2}+\sigma_{3})$$

$$\dot{R_{1}=}-\lambda R_{1}+\beta UR_{1}c_{1}-r_{t}R_{1}\left( 1-\rho\right)\left( \sigma_{2}+\sigma_{3} \right)-r_{r}R_{1}\left( 1-\left( \sigma_{2}+\sigma_{3} \right) \right)-\eta_{rr}R_{1}\rho\sigma_{3}-\eta_{rr}R_{1}\rho\sigma_{2}-\eta_{RW}R_{1}\left( 1-\left( \sigma_{1}+\sigma_{2}+\sigma_{3} \right) \right)+\eta_{WR}\rho WT\sigma_{1}$$

$$\dot{R_{2}=}-\lambda R_{2}+\beta UR_{2}c_{2}-r_{t}R_{2}\left( 1-\rho\right)\left( \sigma_{1}+\sigma_{3} \right)-r_{r}R_{2}\left( 1-\left( \sigma_{1}+\sigma_{3} \right) \right)-\eta_{rr}R_{2}\rho\sigma_{1}-\eta_{rr}R_{2}\rho\sigma_{3}-\eta_{RW}R_{2}\left( 1-\left( \sigma_{1}+\sigma_{2}+\sigma_{3} \right) \right)+\eta_{WR}\rho WT\sigma_{2}$$

$$\dot{R_{3}=}-\lambda R_{3}+\beta UR_{3}c_{3}-r_{t}R_{3}\left( 1-\rho\right)\left( \sigma_{1}+\sigma_{2} \right)-r_{r}R_{3}\left( 1-\left( \sigma_{1}+\sigma_{2} \right) \right)-\eta_{rr}R_{3}\rho\sigma_{1}-\eta_{rr}R_{3}\rho\sigma_{1}-\eta_{RW}R_{3}\left( 1-\left( \sigma_{1}+\sigma_{2}+\sigma_{3} \right) \right)+\eta_{WR}\rho WT\sigma_{3}$$

$$\dot{R_{12}=}-\lambda R_{12}+\beta UR_{12}c_{12}-r_{t}R_{12}\left( 1-\rho\right)\sigma_{3}-r_{rr}R_{12}\left( 1-\sigma_{3} \right)-\eta_{rrr}R_{12}\rho\sigma_{3}-\eta_{RW}R_{12}\left( 1-\left( \sigma_{1}+\sigma_{2}+\sigma_{3} \right) \right)+\eta_{RR}\rho R_{1}\sigma_{2}+\eta_{RR}\rho R_{2}\sigma_{1}$$

$$\dot{R_{13}=}-\lambda R_{13}+\beta XUc_{13}-r_{t}R_{13}\left( 1-\rho\right)\sigma_{2}-r_{rr}R_{13}\left( 1-\sigma_{2} \right)-\eta_{rrr}R_{13}\rho\sigma_{2}-\eta_{RW}R_{13}\left( 1-\left( \sigma_{1}+\sigma_{2}+\sigma_{3} \right) \right)+\eta_{RR}\rho R_{1}\sigma_{3}+\eta_{RR}\rho R_{3}\sigma_{1}$$

$$\dot{R_{23}=}-\lambda R_{23}+\beta UR_{23}c_{23}-r_{t}R_{23}\left( 1-\rho\right)\sigma_{3}-r_{rr}R_{23}\left( 1-\sigma_{2} \right)-\eta_{rrr}R_{23}\rho\sigma_{1}-\eta_{RW}R_{23}\left( 1-\left( \sigma_{1}+\sigma_{2}+\sigma_{3} \right) \right)+\eta_{RR}\rho R_{2}\sigma_{3}+\eta_{RR}\rho R_{3}\sigma_{2}$$

$$\dot{R_{123}=}-\lambda R_{123}+\beta XUc_{123}-r_{rrr}R_{123}-\eta_{rrr}\rho(R_{23}\sigma_{1}+R_{13}\sigma_{2}+R_{12}\sigma_{3})-\eta_{RW}R_{123}\left( 1-\left( \sigma_{1}+\sigma_{2}+\sigma_{3} \right) \right)$$

(eqn S1.1)

*Performance Criteria*

We considered three primary criteria in this study: 1) average decrease in resistance per unit % of overall antibiotic usage decreased, 2) total increase in infections per unit % of overall antibiotic usage decreased and 3) the average number of effective antibiotics over the course of the intervention (defined at baseline at a resistance prevalence of 25%). These criteria are described in the *Materials and Methods* section.

*Caveats to Taxation*

There are several caveats to how taxation was modelled. If usage drops to 0% due to an intervention, then we modelled antibiotic usage for this class as 1% (σ_x_ = 0.01). The rationale behind this was due to a lack of explicit modelling of antibiotic prices in this study, with only relative % changes in price and usage being modelled. Therefore, if usage drops to 0, and there is a % change in usage due to taxation, then usage will not change (σ_x_ = 0). We do not consider this an accurate assumption, so we apply a non-zero level of antibiotic usage (0.01) which will allow antibiotic usage to increase again after a decrease to 0.

*Differential Taxation*

Differential taxation works by scaling the taxation to the level of resistance found in each antibiotic class. Calculation of the scaled taxation rate may occur multiple times with “rounds” of differential taxation at fixed intervals of 3 years (365 x 3 = 1095 days). Scaling factors for taxation are calculated relative to the class with medium levels of resistance, identified before the initiation of the intervention (t = 3000). As an illustrative example, we model three antibiotic classes with a prevalence of resistance of 0.4, 0.25 and 0.1 respectively (Figure S1). At the initiation of the intervention at t = 3000, we calculate scaling factors of 1.6, 1 and 0.4 and apply this to a baseline taxation rate of 50%. This results in taxation (or a change in price) of 80%, 50% and 20% respectively. These are then applied to the baseline PED matrix, resulting in -90%, -27.5% and +57.5% usage of class 1, 2 and 3 antibiotics respectively (σ_1_, σ_2_, σ_3_).


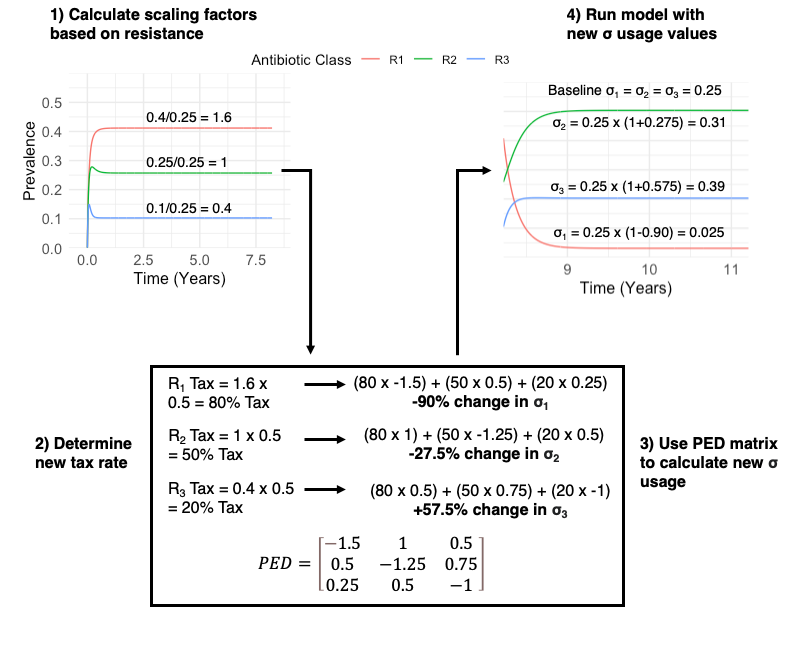


**Figure S1. Illustrative example of the calculation of the first round of differential taxation on antibiotic usage and the dynamics of R_1_, R_2_ and R_3_ antibiotic resistance**. Fitness costs, PED matrix and baseline sigma values used in this example are obtained from the baseline parameterisation responsible for Figure 2.

The next round of differential taxation occurs 1095 days after the initiation of the first round of differential taxation (Figure S2). With a new prevalence of resistance of 0.03, 0.5 and 0.2 for antibiotic class 1, 2 and 3 respectively, we calculate new scaling factors of 0.12, 2 and 0.8. Applied to the baseline taxation rate of 50%, this results in a new tax rate of 6%, 100% and 40% respectively. Note that the scaling factors are still calculated relative to the medium resistance antibiotic class before the initiation of differential taxation (*t* = 3000 days).


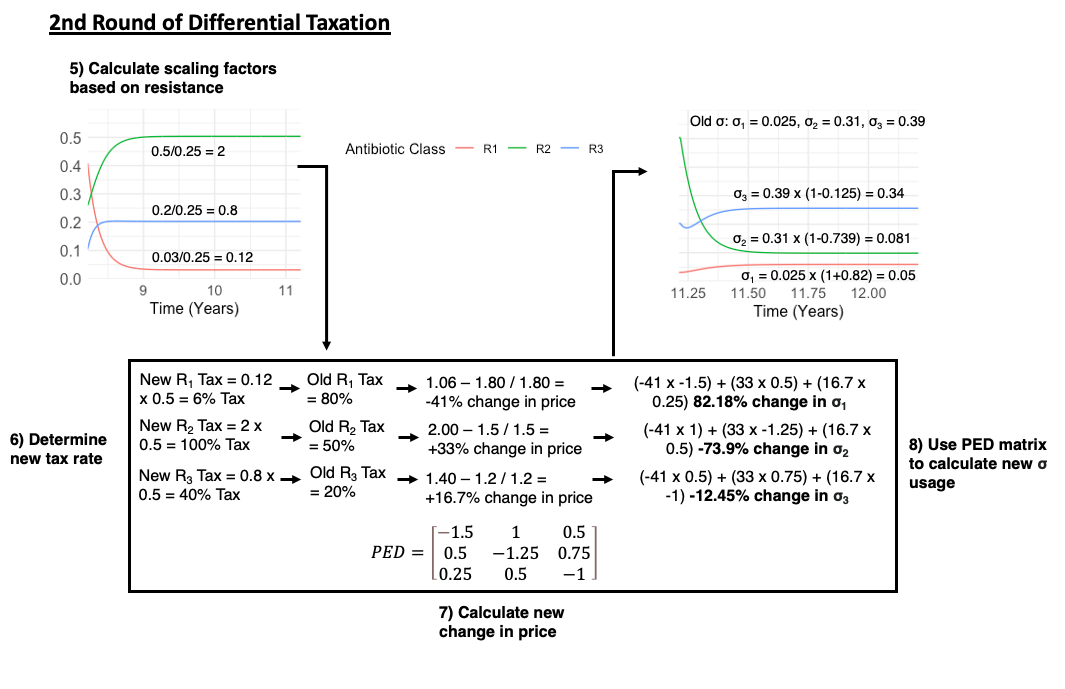


**Figure S2. Illustrative example of the calculation of the second round of differential taxation on antibiotic usage and the dynamics of R_1_, R_2_ and R_3_ antibiotic resistance**. Fitness costs, PED matrix and baseline sigma values used in this example are obtained from the baseline parameterisation responsible for Figure 2.

This new taxation rate should not be applied to antibiotic usage (σ_x_) after the 1^st^ round of differential taxation, which is the usage occurring after the initial round of 80%, 50% and 20% taxation. As an illustrative example, this would be identical to applying a 6% tax on top of an 80% tax for antibiotic class 1. Instead, we calculate the % change in price relative to the price of the antibiotic before the implementation of the intervention (*t* = 3000).

For example, if we assume an arbitrary price of $1, then for antibiotic class 1 in the first round of taxation, this results in an increase in price from $1 to $1.80 ($1 x 1.8). In the second round of differential taxation, taxation is reduced to 6%. This results in a new price of $1.06 ($1 x 1.06). Therefore, the change in price from the first to the second round of differential taxation can be considered ($1.06 - $1.80) / $1.80 = -41.1%. We apply this change in price to the PED matrix resulting in σ_1_ = 0.05, σ_2_ = 0.081 and σ_3_ = 0.34 respectively. Additionally, if differential taxation results in an increase in usage which drives σ_1_ + σ_2_ + σ_3_ > 1 at any stage during differential taxation, then total usage is rescaled to be bound between 0 and 1.

*ABC SMC Parameter Inference*

An approximate Bayesian computation sequential monte Carlo (ABC-SMC) approach was used to estimate the marginal posterior probability distribution for twelve model parameters (θ) given the data$, \theta=\{\beta,\eta_{wr},\eta_{rw},\eta_{rr},\eta_{rrr},c_{1},c_{2},c_{3},c_{12},c_{13},c_{23},c_{123}\}$ [1, 2]. This was done to fit the three modelled antibiotic classes to three target prevalence values for resistance: 40%, 25% and 10%.

Other model parameters were not fitted as estimates with high levels of certainty were available (*λ*) or due to the lack of importance of the model parameters for promoting heterogeneity in resistance among modelled antibiotic classes (*ρ*, *r_wt_*, *r_t_*, *r_r_*, *r_rr_*, *r_rrr_*)

Both *η_rr_* and *η_rrr_* were assumed to hold the same value in the baseline illustrative example scenario to maintain simplicity of parameterisation and due to the lack of importance of both parameters for maintaining heterogeneity in resistance across antibiotic classes. Therefore ABC-SMC was used to fit both parameters with the same value. These two parameters were allowed to individually vary in subsequent sensitivity analyses.

Transmission-related fitness costs of resistance were assumed to have a hierarchical relationship of *c_1_* = *c_2_*= *c_3_* > *c_12_* = *c_13_* = *c_23_* > *c_123_*. The rate of conversion from resistance-to-wild type infection was also assumed to be lower than the rate of conversion from wild type-to-resistance. This was assumed due to a priori exploration of the model, where only *η_WR_* > *η_RW_* promoted heterogeneity between R_1_, R_2_ and R_3_ resistances, which is what is observed through surveillance data.

A decision was made to use differences in transmission related fitness costs of resistance to promote differences in resistance, rather than heterogeneity in antibiotic usage (*σ_1_*, *σ_2_*, *σ_3_*) for the illustrative baseline scenario. This was only performed for baseline scenarios and antibiotic usage and fitness costs were allowed to vary in subsequent sensitivity analysis. Prior distributions for each fitted parameter can be found in Table S1.

**Table S1. Prior distributions used for ABC-SMC model fitting**

| Parameter | Description | Prior Distribution |
| --- | --- | --- |
| *β* | Per capita rate of transmission between infected and susceptible food animals | $U\left( 0, 10 \right)$ |
| *η_wr_* | Per capita rate of conversion from wild-type to antibiotic-resistant infection | $U\left( 0, 4 \right)$ |
| *η_rw_* | Per capita rate of reversion from antibiotic-resistant to wild-type infection | $U\left( 0, 4 \right)$ |
| *η_rr_* | Per capita rate of conversion from singly resistant to doubly resistant infection | $U\left( 0,0.5 \right)$ |
| *η_rrr_* | Per capita rate of conversion from doubly resistant to triply resistant infection | $U\left( 0,0.5 \right)$ |
| *c_1_* | Transmission-related fitness cost related to infection with resistance to class 1 antibiotic | $U\left( 0.5,1 \right)$ |
| *c_2_* | Transmission-related fitness cost related to infection with resistance to class 2 antibiotic | $U\left( 0.5,1 \right)$ |
| *c_3_* | Transmission-related fitness cost related to infection with resistance to class 3 antibiotic | $U\left( 0.5,1 \right)$ |
| *c_12_* | Transmission-related fitness cost related to infection with resistance to class 1 and 2 antibiotic | $U\left( 0.5,1 \right)$ |
| *c_13_* | Transmission-related fitness cost related to infection with resistance to class 1 and 3 antibiotic | $U\left( 0.5,1 \right)$ |
| *c_23_* | Transmission-related fitness cost related to infection with resistance to class 2 and 3 antibiotic | $U\left( 0.5,1 \right)$ |
| *c_123_* | Transmission-related fitness cost related to infection with resistance to class 1, 2 and 3 antibiotic | $U\left( 0.5,1 \right)$ |

The ABC-SMC model fit was run for ten generations, with each generation running until the acceptance of 1000 particles. Acceptance thresholds (*ε*) were required for each of the three target values, calculating a distance measure between the modelled and target value. These were generated through an iterative trial-error process to ensure that distance measures and summary statistics were as close to target values, while still ensuring that the ABC-SMC process was time-efficient. Target thresholds were the target prevalence multiplied by scaling factors of 1, 0.8, 0.6, 0.4, 0.3, 0.2, 0.15, 0.1, 0.05 and 0.01 for each subsequent generation.

A multivariate normal distribution was chosen for the ABC-SMC perturbation kernel [2], with the randomly sampled mean and covariance matrix calculated from the previously accepted generation of accepted particles. An intersection metric was used to ensure that accepted particles satisfied tolerance values set for the distance measure for each generation. Approximated posterior distributions can be found below (Figure S3).


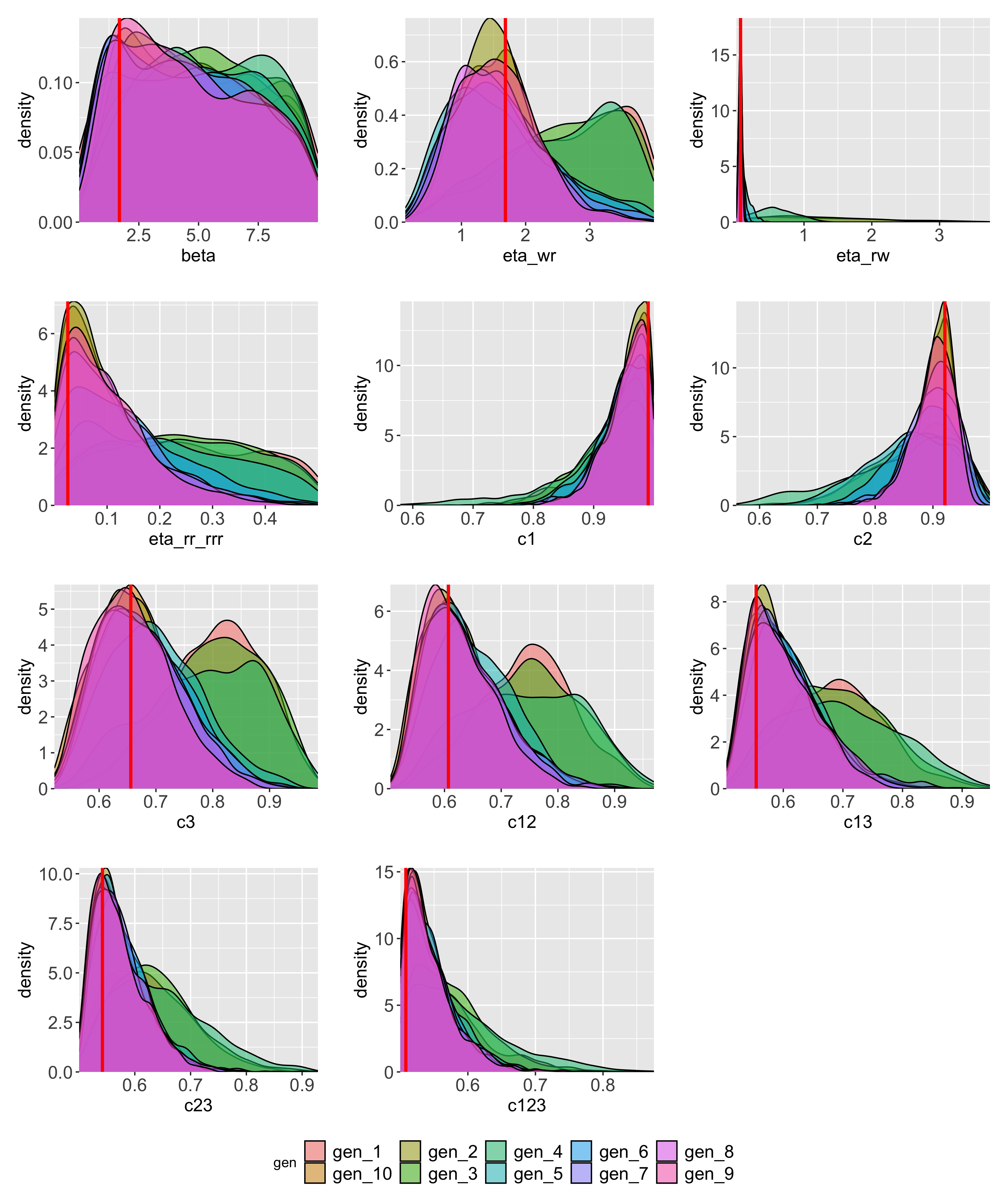


**Figure S3. Approximated posterior distributions for the fitted model parameters.** The estimated posterior distribution for each generation is highlighted by fill colours. Red line represents the mean from the 10^th^ generation for each parameter.

Mean point estimates from the posterior probability distributions of the 10^th^ accepted generation were used as the final parameter sets for each respective case study (Table S2).

**Table S2. Fitted parameter values and descriptions used for baseline.**

| Parameter | Description | (Fitted) Parameter Values^1^ |
| --- | --- | --- |
| *λ* | Per capita birth/death rate in food animals | 365^-1^ days |
| *β* | Per capita rate of transmission between infected and susceptible food animals | **4.919** |
| *σ_x_* | Proportion of the population using class X antibiotic | 0.25 at baseline |
| *r_wt_* | Per capita rate of recovery for food animals with wild type infection | 12^-1^ days |
| *r_r_* | Per capita rate of recovery for food animals with singly resistant infection | 10^-1^ days |
| *r_rr_* | Per capita rate of recovery for food animals with doubly resistant infection | 9^-1^ days |
| *r_rrr_* | Per capita rate of recovery for food animals with triply resistant infection | 8^-1^ days |
| *r_t_* | Per capita rate of recovery for food animals successfully treated with antibiotic | 7^-1^ days |
| *η_wr_* | Per capita rate of conversion from wild-type to antibiotic-resistant infection | **1.531** |
| *η_rw_* | Per capita rate of reversion from antibiotic-resistant to wild-type infection | **0.062** |
| *η_rr_* | Per capita rate of conversion from singly resistant to doubly resistant infection | **0.094** |
| *η_rrr_* | Per capita rate of conversion from doubly resistant to triply resistant infection | **0.094** |
| *c_1_* | Transmission-related fitness cost related to infection with resistance to class 1 antibiotic | **0.956** |
| *c_2_* | Transmission-related fitness cost related to infection with resistance to class 2 antibiotic | **0.903** |
| *c_3_* | Transmission-related fitness cost related to infection with resistance to class 3 antibiotic | **0.664** |
| *c_12_* | Transmission-related fitness cost related to infection with resistance to class 1 and 2 antibiotic | **0.626** |
| *c_13_* | Transmission-related fitness cost related to infection with resistance to class 1 and 3 antibiotic | **0.600** |
| *c_23_* | Transmission-related fitness cost related to infection with resistance to class 2 and 3 antibiotic | **0.596** |
| *c_123_* | Transmission-related fitness cost related to infection with resistance to class 1, 2 and 3 antibiotic | **0.541** |
| *ρ* | Probability of treatment failure upon exposure to antibiotic (for which the infection is susceptible to) | 0.05 |
| *Base Tax* | Baseline taxation rate | 0.5 (50%) |

^1^Note that values in bold are mean point estimates from the posterior distribution of fitted parameters.

*Uncertainty Analysis*

An uncertainty analysis was conducted to the assess the efficacy of each explored intervention to maximise or minimise the three criteria across a range of realistic parameter values. This was done by using Monte-Carlo sampling to draw parameter values for each explored parameter. These were *λ*, *β*, *σ_1_*, *σ_2_*, *σ_3_*, *r_WT_*, *r_r_*, *r_rr_*, *r_rrr_*, *r_t_*, *η_WR_*, *η_RW_*, *η_rr_*, *η_rrr_*, *c_1_*, *c_2_*, *c_3_*, *c_12_*, *c_13_*, *c_23_*, *c_123_*, and *ρ* parameters. Parameters such as the PED matrix, base tax rate, intervention start date and time between interventions were fixed.

The upper and lower bounds of each explored parameter was set as roughly an order of magnitude below and above the value obtained from the ABC-SMC inference approach. The exception was with transmission related fitness cost of resistance parameters which were bound between 0.5-1. Transmission-related fitness costs of resistance were assumed to have a hierarchical relationship of *c_1_* ≈ *c_2_* ≈ *c_3_* > *c_12_* ≈ *c_13_* ≈ *c_23_* > *c_123_*. The rate of conversion from resistance-to-wild type infection was also assumed to be lower than the rate of conversion from wild type-to-resistance. This was assumed due to *a priori* exploration of the model, where only *η_WR_* > *η_RW_* promoted heterogeneity between R_1_, R_2_ and R_3_.

The rate of recovery was also assumed to follow a specific hierarchy, *r_WT_* > *r_r_* > *r_rr_* > *r_rrr_* > *r_T_*, based on the assumption that the accumulation of resistance genes is deleterious and have detrimental impact on the fitness of the bacteria. Fitter bacteria were assumed to have an enhanced ability to establish infectious populations within infected livestock and therefore the period of infectiousness is longer (1/*r_x_*). Treatment with effective antibiotics was assumed to result in the lowest fitness and subsequently the shortest infectious period.

N = 1,000 parameter combinations were run and values for the performance criteria were generated. If a parameter set resulted in a disease-free equilibrium, then parameters were redrawn and ran again until a non-trivial disease equilibrium was identified.

*Intervention Failure*

Parameter combinations which resulted in an increase in both overall antibiotic usage and average resistance were not included in the uncertainty analyses. These scenarios were denoted as having experienced “intervention failure”. The rationale for this exclusion was the potential for confounding the performance criteria.

Due to the relative nature of the performance criteria (change in resistance per D-ABU), a 50% *increase* in average resistance and overall usage compared to baseline usage, is identical to a 50% *decrease* in average resistance and overall usage compared to baseline usage (Figure S4). Both scenarios result in a change in average resistance per D-AMU of 1%. Therefore, we exclude “intervention failure” scenarios from uncertainty analyses but use these values as weights for the performance metric used in Figure 2-3.

Intervention failure results primarily from 1) high cross elasticity of demand, or 2) targeting the lowest resistance antibiotic class. The former results from large compensatory increases in usage beyond what is being reduced through taxation. The latter is due to compensatory increases in usage occurring in antibiotic classes with high resistance, which is associated with high levels of usage and low fitness costs of resistance. This ultimately leads to disproportionately greater compensatory increases in overall resistance and usage when curtailment occurs.

**
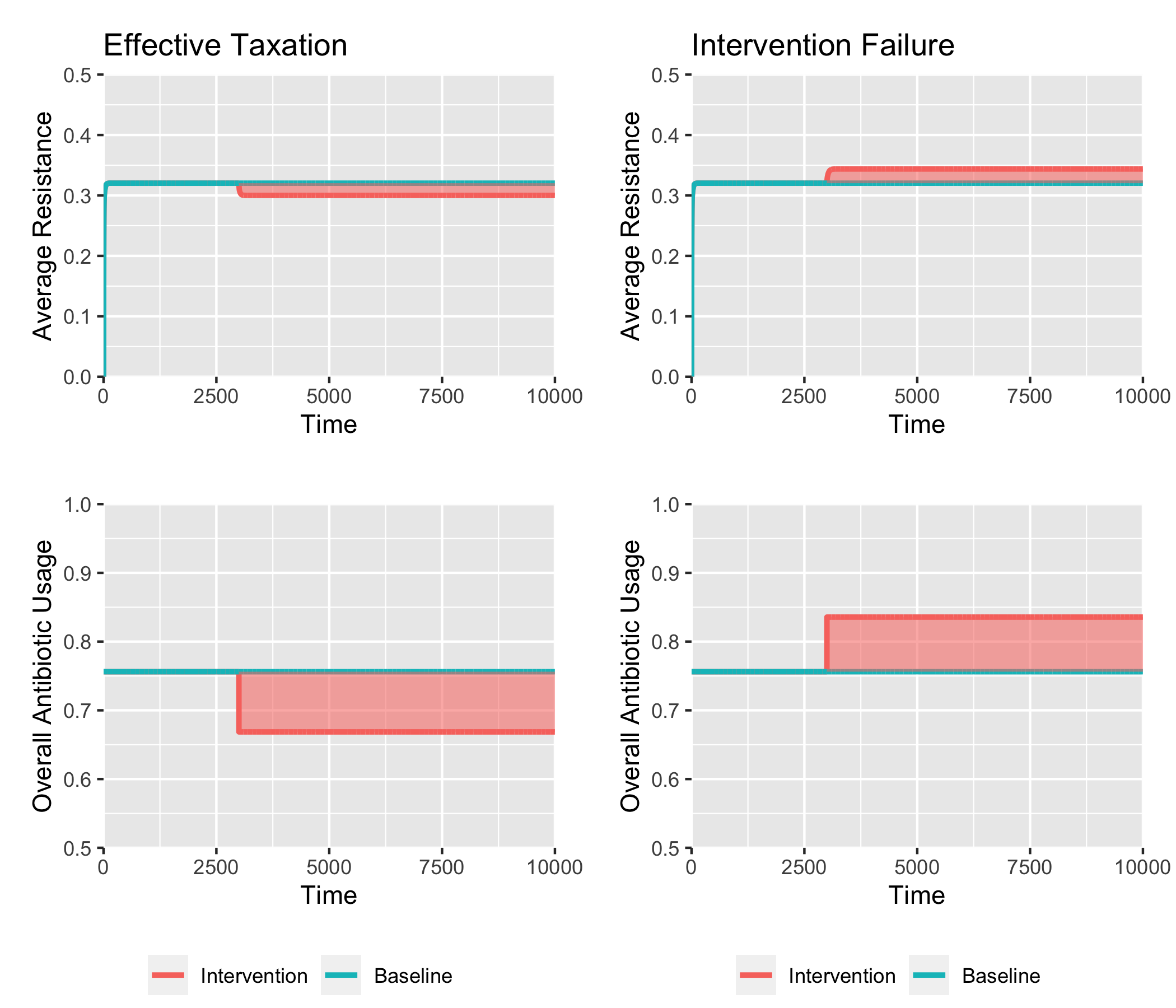
**

**Figure S4. Comparison of an “effective intervention” versus “intervention failure” with regards to changes in the average resistance and overall antibiotic usage.** Effective interventions result in a decrease in resistance and usage relative to baseline (the integral of which is defined in the filled red area) (left). Intervention failure is defined as an intervention resulting in an increase in resistance and usage relative to baseline (right).

*Statistical Analysis and Comparison*

A Shapiro-Wilks test was used to assess the normality of the distributions for the % change in average resistance, overall infections and maximising the average number of available antibiotics. These tests identified non-normality in the data for the average resistance and total infections optimisation criteria. Pairwise non-parametric Wilcoxon signed ranked tests were used to compare the distribution of the optimal strategy for each optimisation criteria against all others (Table S3-4). Due to the use of multiple pairwise comparisons, a Bonferroni correction was applied. We note that only effect sizes were reported in the main text, as care must be taken with the interpretation of significance testing, with high number of Monte-Carlo simulations making biologically unmeaningful differences between interventions significant [3].

**Table S3. Significance testing using Wilcoxon signed-ranked tests for each scenario.** Comparing the most optimal intervention against all other interventions for the average resistance performance criteria.

| **Tax Strategy** | **Scenarios** | | | | | | | | | | | | | | | | |
| --- | --- | --- | --- | --- | --- | --- | --- | --- | --- | --- | --- | --- | --- | --- | --- | --- | --- |
|  | **Baseline** | **5% Thresh** | **10% Thresh** | **35% Thresh** | **10% Tax** | **25% Tax** | **75% Tax** | **90% Tax** | **Intensive**  **Chickens** | **Extensive**  **Chickens** | **Intensive**  **Cattle** | **Extensive**  **Cattle** | **Two Classes** | **Four Classes** | **Low Comp PED** | **High Comp PED** | **All PED Vary** |
| **Flat Tax** | 1.0000 | 1.0000 | 1.0000 | 1.0000 | 1.0000 | 1.0000 | 1.0000 | 1.00000 | 1.00000 | 0.0010 | 0.45878 | 1.7e-05 | **Optimal** | 1.00000 | 0.89304 | 1.00000 | 0.00406 |
| **Single Tax (HR)** | 1.0000 | 1.0000 | 1.0000 | 1.0000 | 0.0405 | 0.25523 | 1.0000 | 1.00000 | 1.00000 | 1.0000 | 1.00000 | 1.0000 | 1.00000 | 1.00000 | 0.61988 | 0.05519 | 1.00000 |
| **Single Tax (MR1)** | 1.0000 | 1.0000 | 1.000 | 1.0000 | 1.0000 | 1.00000 | 0.28263 | 0.00030 | 1.00000 | 2.8e-10 | 0.32844 | 7.2e-12 |  | 1.00000 | 1.00000 | 4.4e-08 | 1.00000 |
| **Single Tax (MR2)** |  |  |  |  |  |  |  |  |  |  |  |  |  | 1.00000 |  |  |  |
| **Single Tax (LR)** | 6.9e-15 | 1.0e-13 | 2.6e-15 | <2e-16 | 1.0000 | 0.00049 | <2e-16 | 2.4e-13 | 8.2e-11 | < 2e-16 | 7.7e-08 | 1.0000 | 0.63072 | 1.00000 | 0.00024 | 4.7e-05 | 0.00198 |
| **Diff Tax**  **(1 Rd)** | 1.0000 | 1.0000 | 0.278 | 1.0000 | 1.0000 | 0.14974 | 1.0000 | 1.00000 | 0.07616 | 1.0000 | 1.00000 | 1.0000 | 1.00000 | 0.31307 | 0.00043 | 3.9e-13 | 1.00000 |
| **Diff Tax**  **(2 Rd)** | 1.0000 | 1.0000 | 0.055 | 1.0000 | 1.0000 | 0.12793 | 0.00321 | 0.04215 | 0.00079 | 1.0000 | 0.70649 | 1.0000 | 1.00000 | 0.00254 | 0.00073 | 1.0e-10 | 1.00000 |
| **Diff Tax**  **(3 Rd)** | 1.0000 | 1.0000 | 0.123 | 1.0000 | 1.0000 | 0.10964 | 0.02018 | 0.18178 | 0.00168 | 0.6032 | 1.00000 | 1.0000 | 1.00000 | 0.00124 | 0.00028 | 4.2e-10 | 1.00000 |
| **Diff Tax**  **(4 Rd)** | 1.0000 | 1.0000 | 0.089 | 1.0000 | 1.0000 | 0.16176 | 0.01528 | 0.13324 | 0.00132 | 0.4156 | 1.00000 | 1.0000 | 1.00000 | 0.00210 | 0.00032 | 6.1e-09 | 1.00000 |
| **Diff Tax (5 Rd)** | 1.0000 | 1.0000 | 0.106 | 1.0000 | 1.0000 | 0.15539 | 0.01857 | 0.11695 | 0.00148 | 0.2881 | 1.00000 | 1.0000 | 1.00000 | 0.00097 | 0.00035 | 1.8e-09 | 1.00000 |
| **Diff Tax**  **(6 Rd)** | 1.0000 | 1.0000 | 0.087 | 1.0000 | 1.0000 | 0.12563 | 0.01132 | 0.09409 | 0.00107 | 0.2847 | 1.00000 | 1.0000 | 1.00000 | 0.00112 | 0.00036 | 9.8e-10 | 0.85000 |
| **Ban**  **(HR)** | **Optimal** | **Optimal** | **Optimal** | **Optimal** | **Optimal** | **Optimal** | **Optimal** | **Optimal** | **Optimal** | **Optimal** | **Optimal** | **Optimal** | **0.03252** | **Optimal** | 1.00000 | **Optimal** | **Optimal** |
| **Ban (MR1)** | 0.0016 | 1.0000 | 3.4e-09 | 0.0016 | 0.00576 | 6.6e-05 | 5.9e-07 | 3.0e-07 | 0.01576 | < 2e-16 | 0.02740 | 1.4e-11 |  | 1.0e-09 | **Optimal** | 4.1e-15 | 3.1e-09 |
| **Ban (MR2)** |  |  |  |  |  |  |  |  |  |  |  |  |  | 1.00000 |  |  |  |
| **Ban (LR)** | <2e-16 | <2e-16 | <2e-16 | 1.0e-07 | 1.8e-15 | <2e-16 | < 2e-16 | 8.5e-16 | < 2e-16 | < 2e-16 | 1.3e-12 | < 2e-16 | 3.4e-10 | 1.7e-05 | 4.9e-13 | 0.54408 | < 2e-16 |

^1^Note that the most optimal strategy for each scenario (column) is denoted by a shaded in box. ^2^HR = High Resistance, MR1 = 2^nd^ Lowest Resistance, MR2 = 3^rd^ Lowest Resistance, LR = Low Resistance.

**Table S4. Significance testing using Wilcoxon signed-ranked tests for each scenario.** Comparing the most optimal intervention against all other interventions for the overall infections performance criteria.

| **Tax Strategy** | **Scenarios** | | | | | | | | | | | | | | | | |
| --- | --- | --- | --- | --- | --- | --- | --- | --- | --- | --- | --- | --- | --- | --- | --- | --- | --- |
|  | **Baseline** | **5% Thresh** | **10% Thresh** | **35% Thresh** | **10% Tax** | **25% Tax** | **75% Tax** | **90% Tax** | **Intensive**  **Chickens** | **Extensive**  **Chickens** | **Intensive**  **Cattle** | **Extensive**  **Cattle** | **Two Classes** | **Four Classes** | **Low Comp PED** | **High Comp PED** | **All PED Vary** |
| **Flat Tax** | 7.3e-07 | 1.1e-07 | 0.00018 | 0.00272 | 0.00030 | 0.00052 | 1.00000 | 8.1e-06 | 0.42406 | 0.00087 | 3.4e-08 | 7.7e-06 | 0.03252 | 1.00000 | 0.00230 | 1.9e-15 | < 2e-16 |
| **Single Tax (HR)** | 3.2e-12 | 5.2e-09 | 6.4e-06 | 8.5e-05 | 1.00000 | 0.66960 | 1.00000 | 1.6e-09 | 0.80626 | 4.6e-06 | 3.4e-10 | 9.7e-08 | 4.5e-05 | 0.50214 | 2.5e-06 | 1.1e-10 | < 2e-16 |
| **Single Tax (MR1)** | 0.03336 | 1.00000 | 0.00032 | 0.10041 | 1.7e-06 | 1.6e-05 | 0.28263 | 1.00000 | 0.01172 | 0.00036 | 2.3e-06 | 1.9e-05 |  | **Optimal** | 1.00000 | 1.00000 | 2.9e-05 |
| **Single Tax (MR2)** |  |  |  |  |  |  |  |  |  |  |  |  |  | 1.00000 |  |  |  |
| **Single Tax (LR)** | 1.00000 | 1.4e-06 | 1.00000 | 1.00000 | 0.52092 | 1.00000 | 1.00000 | 1.00000 | 1.00000 | 1.00000 | 1.000 | 1.000 | 0.00028 | 1.00000 | 3.8e-14 | 0.02428 | 1.1e-11 |
| **Diff Tax**  **(1 Rd)** | 1.2e-12 | 1.7e-14 | 5.8e-08 | 1.7e-07 | 9.8e-05 | 1.3e-06 | 4.9e-10 | 7.2e-09 | 0.12087 | 1.3e-05 | 5.6e-09 | 2.9e-08 | 0.00019 | 0.18013 | 0.00173 | 3.6e-15 | < 2e-16 |
| **Diff Tax**  **(2 Rd)** | 6.0e-16 | 9.7e-15 | 1.1e-12 | 6.1e-11 | 2.2e-06 | 2.0e-09 | < 2e-16 | 9.1e-15 | 0.00199 | 7.2e-08 | 2.7e-13 | 1.7e-10 | 0.00029 | 0.00112 | 0.07179 | < 2e-16 | < 2e-16 |
| **Diff Tax**  **(3 Rd)** | <2e-16 | < 2e-16 | 3.8e-13 | 2.1e-12 | 1.7e-06 | 5.5e-10 | < 2e-16 | < 2e-16 | 0.00033 | 1.2e-08 | 8.6e-14 | 1.3e-11 | 0.00025 | 0.00039 | 0.15183 | < 2e-16 | < 2e-16 |
| **Diff Tax**  **(4 Rd)** | <2e-16 | < 2e-16 | 1.6e-13 | 1.4e-12 | 8.5e-07 | 2.2e-10 | < 2e-16 | < 2e-16 | 0.00045 | 6.8e-09 | 5.3e-14 | 1.3e-11 | 0.00026 | 0.00043 | 0.13880 | < 2e-16 | < 2e-16 |
| **Diff Tax (5 Rd)** | <2e-16 | < 2e-16 | 7.7e-14 | 5.9e-13 | 8.9e-07 | 4.0e-10 | < 2e-16 | < 2e-16 | 0.00032 | 1.5e-09 | 4.2e-14 | 4.0e-12 | 0.00025 | 0.00036 | 0.15286 | < 2e-16 | < 2e-16 |
| **Diff Tax**  **(6 Rd)** | <2e-16 | < 2e-16 | 1.7e-13 | 5.3e-14 | 8.6e-07 | 1.2e-10 | < 2e-16 | < 2e-16 | 0.00017 | 9.0e-10 | 2.9e-14 | 2.8e-12 | 0.00020 | 0.00021 | 0.15544 | < 2e-16 | < 2e-16 |
| **Ban (HR)** | <2e-16 | 5.8e-08 | 2.6e-05 | 3.7e-06 | 9.9e-12 | 1.3e-08 | 7.0e-11 | 8.2e-08 | 0.00173 | 1.8e-07 | 7.4e-11 | 5.9e-09 | **Optimal** | 0.04824 | 0.68445 | < 2e-16 | 2.9e-14 |
| **Ban (MR1)** | **Optimal** | **Optimal** | **Optimal** | **Optimal** | **Optimal** | **Optimal** | **Optimal** | **Optimal** | **Optimal** | **Optimal** | **Optimal** | **Optimal** |  | 1.3e-05 | **Optimal** | **Optimal** | **Optimal** |
| **Ban (MR2)** |  |  |  |  |  |  |  |  |  |  |  |  |  | **1.00000** |  |  |  |
| **Ban (LR)** | <2e-16 | 8.4e-16 | 1.00000 | 1.00000 | 1.00000 | 1.00000 | 1.00000 | 1.00000 | 0.06933 | 1.00000 | 1.000 | 1.000 | 1.9e-15 | 1.00000 | <2e-16 | 1.00000 | 0.01961 |

^1^Note that the most optimal strategy for each scenario (column) is denoted by a shaded in box. ^2^HR = High Resistance, MR1 = 2^nd^ Lowest Resistance, MR2 = 3^rd^ Lowest Resistance, LR = Low Resistance.

*Incrementing the Taxation Rate*

To explore similarities between the effect size for taxation and bans on antibiotic usage. Single taxation on high resistance (HR) classes was incremented from 1% to 100%, at 5% intervals. At every taxation increment, an uncertainty analysis (n = 1000) was conducted using the same sampling and parameter ranges as in the main analysis. This allowed for the identification of uncertainty in the estimate for the change in resistance per unit decrease in usage (Figure S5).

This was also conducted for bans on the HR class. However, *a priori*, taxation rate has no impact on bans, so this would not change the efficacy of the intervention. Instead, we included this as a direct comparison to the taxation analysis (Figure S6).


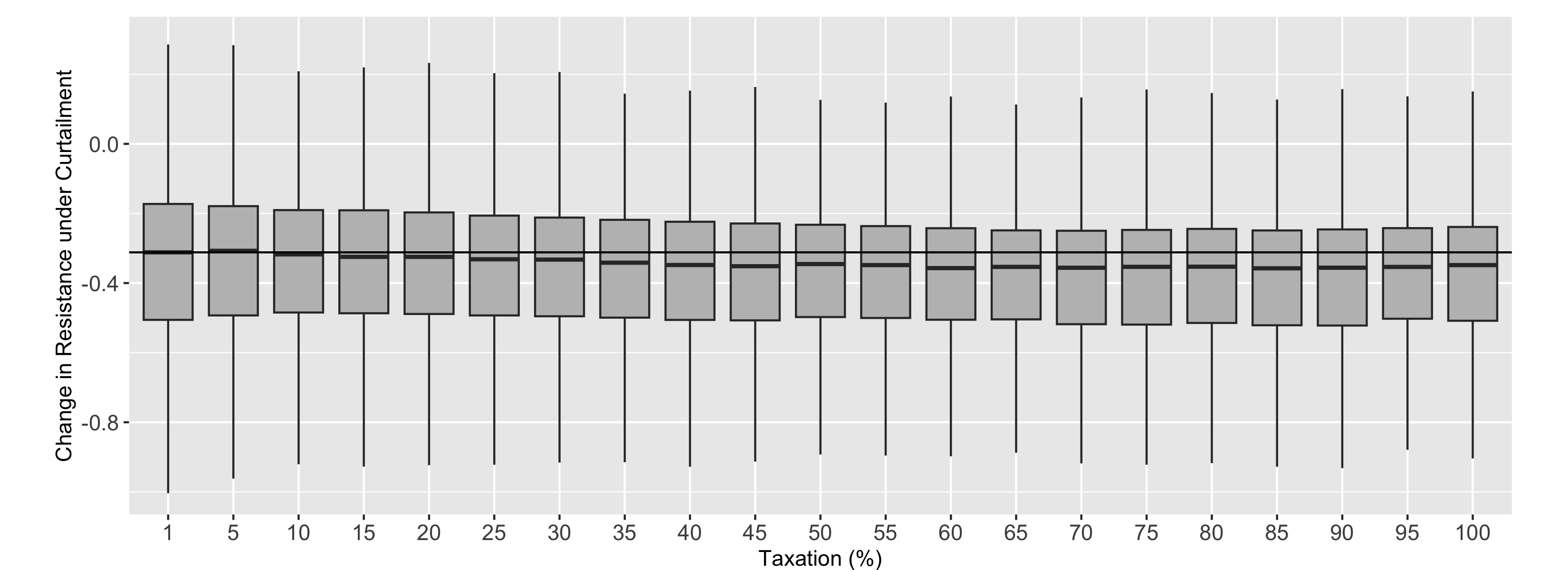
**Figure S5. Impact of incrementing single taxation on the high resistance class from 1% to 100% on the changes to average resistance under total antibiotic curtailment.** The horizontal line represents the median efficacy of the intervention at 1% taxation.


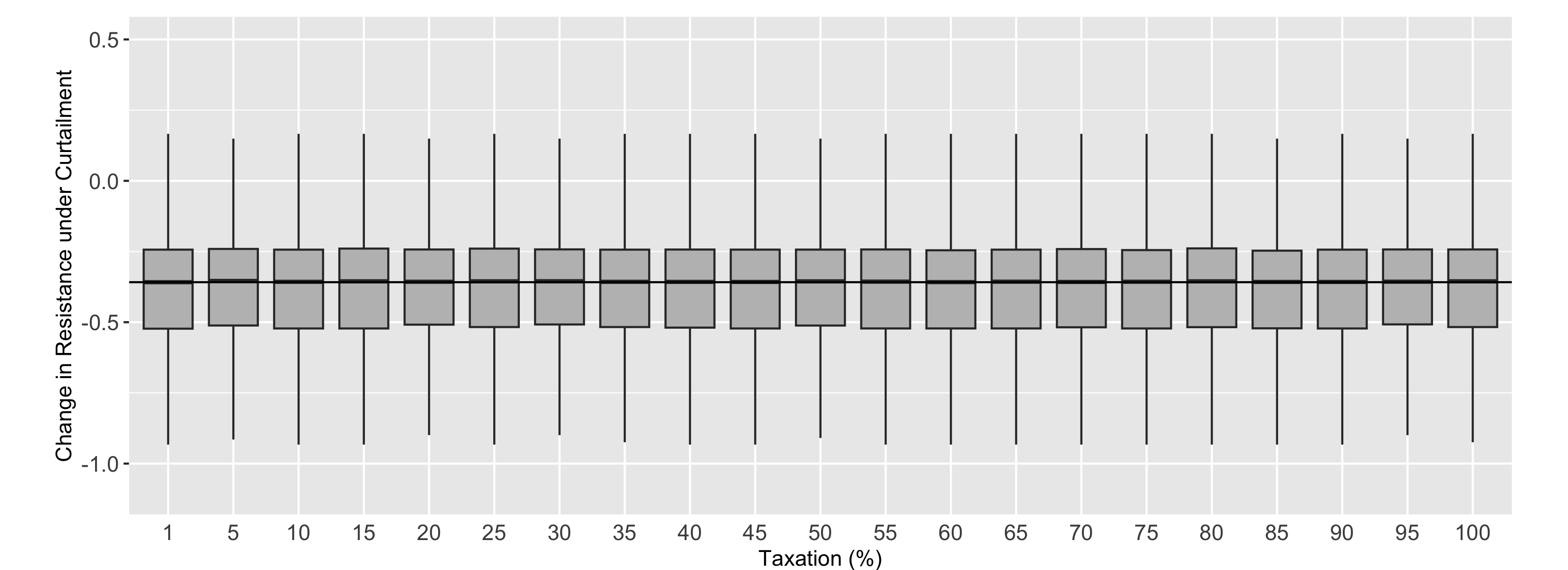


**Figure S6. Impact of incrementing bans on the high resistance class from 1% to 100% on the changes to average resistance under total antibiotic curtailment.** The horizontal line represents the median efficacy of the intervention at 1% taxation.

*Changes to Outcome Measure*

Two alternative outcome measures were explored for the uncertainty analysis. The first measure removes the denominator of the original outcome measure (eqn 1.3-4) and calculates the change in average resistance and total infections over the course of the intervention (eqn S1.2-3) (Figure S7).

$$Resistance Reduction=\frac{\int_{t=3000}^{t=10300} {AvgRes}_{Base} -{AvgRes}_{Int}dt}{10300-3000}$$

(eqn S1.2)

$$Infection Reduction=\frac{\int_{t=3000}^{t=10300} {AvgInf}_{Base} -{AvgInf}_{Int}dt}{10300-3000}$$

(eqn S1.3)


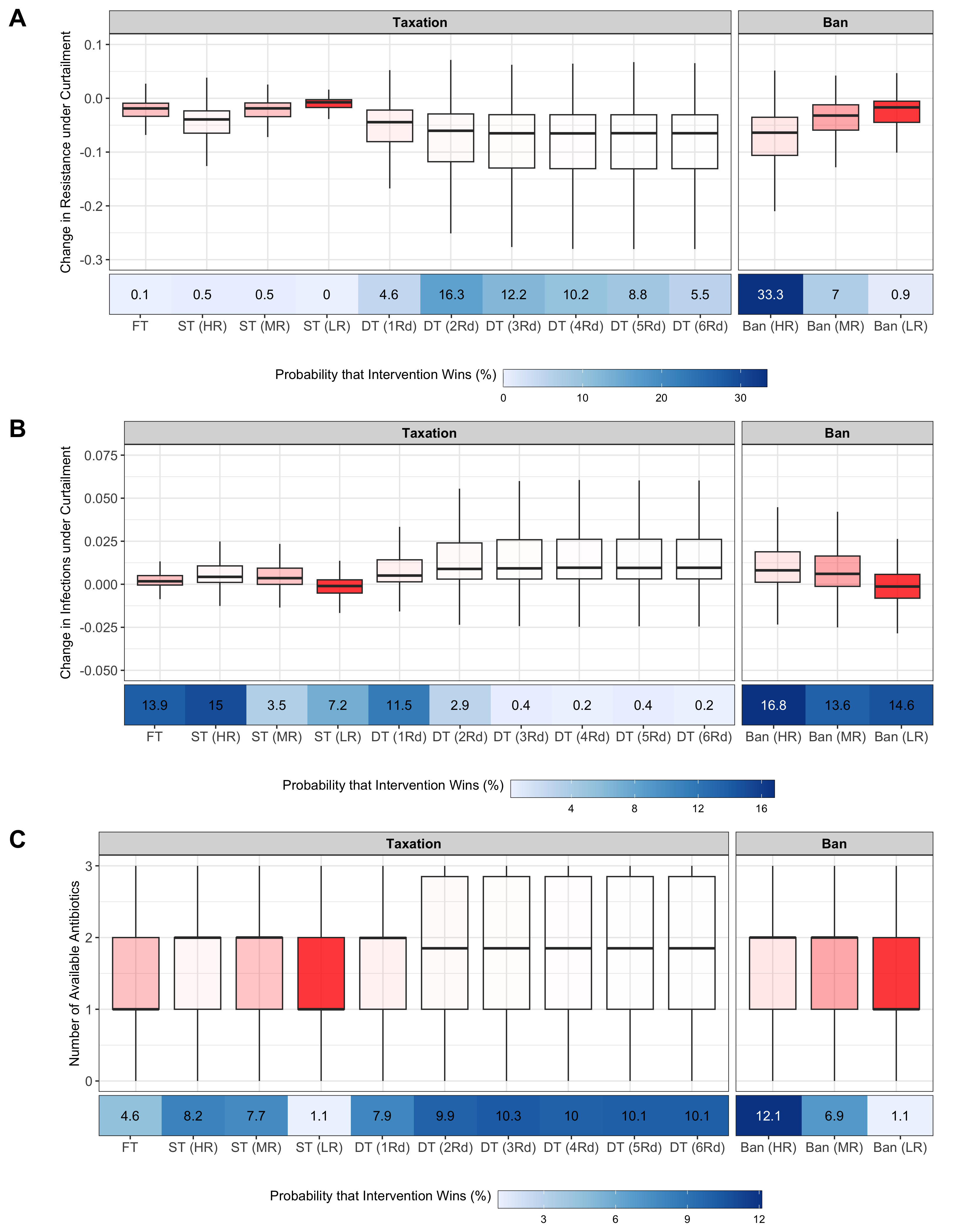


**Figure S7. A) Changes to average resistance, B) changes to overall infections, C) Number of available antibiotics.** FT = Flat Tax, ST = Single Tax, DT = Differential Tax, HR = High Resistance, MR = Medium Resistance and LR = Low Resistance. Multiple rounds of differential taxation were explored (1-6Rds). The intensity of box plot shading represents the proportion of runs resulting in increases to both usage and resistance, representing intervention failure and being identical to the original analysis (also used for weighting of intervention performance: 27.5%, 2.1%, 25.3%, 80.7%, 5.2%, 2.8%, 1%, 0.9%, 0.9%, 0.9%, 7.9%, 36% and 80.3%).

The second measure explores the % change in average resistance/overall infections per % change in antibiotic usage (eqn S1.4-5). This describes the relative % change in resistance/infections and usage from baseline levels for both the numerator and the denominator in the original outcome measure (eqn 1.4-5) (Figure S8).

$$Change in Resistance=\frac{\int_{t=3000}^{t=10300} ({AvgRes}_{Base} -{AvgRes}_{Int})/{AvgRes}_{Base} dt}{\int_{t=3000}^{t=10300} {(Usage}_{Base}-{Usage}_{Int})/{Usage}_{Base} dt}$$

(eqn S1.4)

$$Change in Infection=\frac{\int_{t=3000}^{t=10300} {(TotInf}_{Int}-{TotInf}_{Base})/{TotInf}_{Int} dt}{\int_{t=3000}^{t=10300} {(Usage}_{Base}-{Usage}_{Int})/{Usage}_{Base} dt}$$

(eqn S1.5)


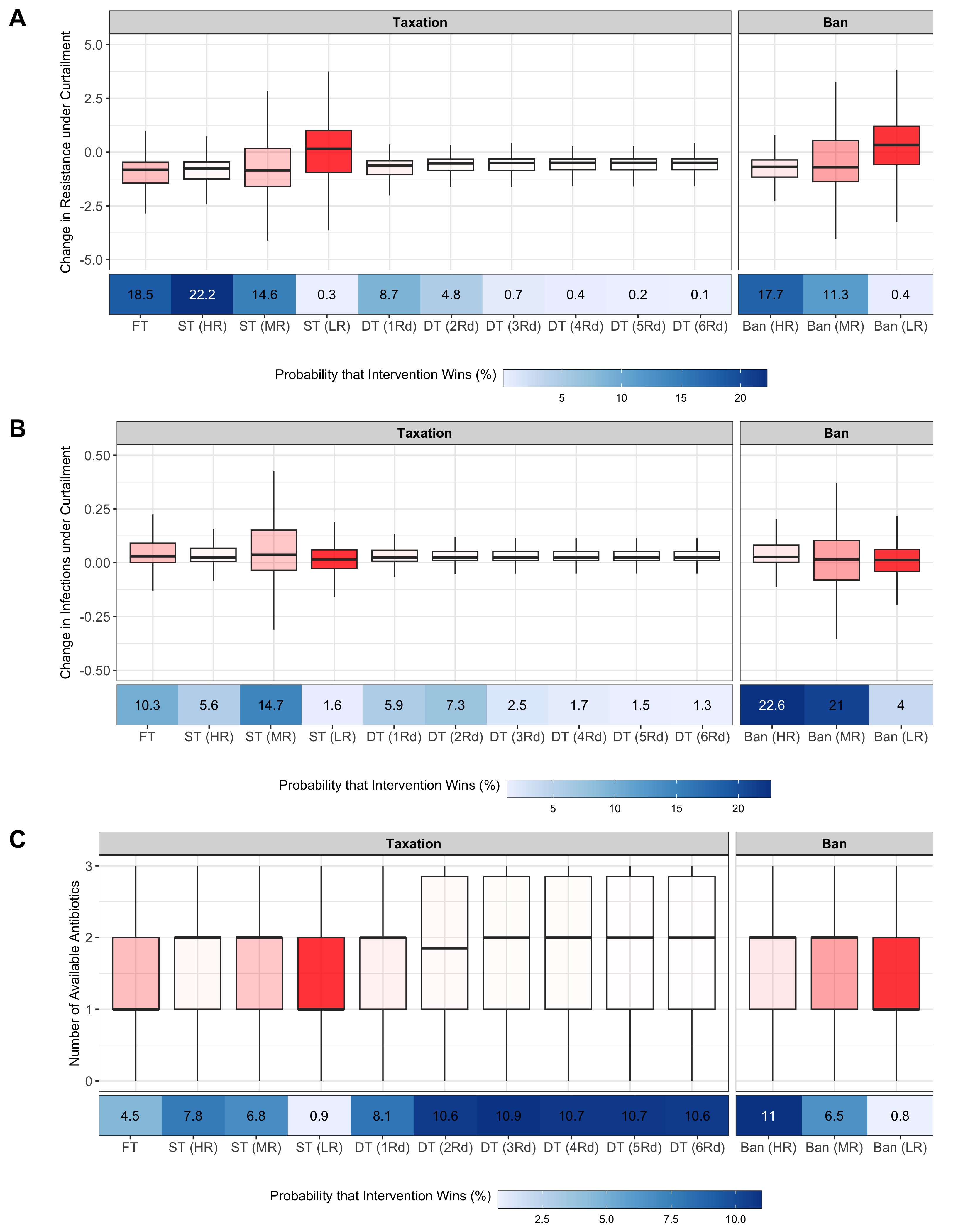


**Figure S8. A) % change to average resistance per % change in antibiotic usage, B) % change to overall infections per % change in antibiotic usage, C) Number of available antibiotics.** FT = Flat Tax, ST = Single Tax, DT = Differential Tax, HR = High Resistance, MR = Medium Resistance and LR = Low Resistance. Multiple rounds of differential taxation were explored (1-6Rds). The intensity of box plot shading represents the proportion of runs resulting in increases to both usage and resistance, representing intervention failure and being identical to the original analysis (also used for weighting of intervention performance: 27.5%, 2.1%, 25.3%, 80.7%, 5.2%, 2.8%, 1%, 0.9%, 0.9%, 0.9%, 7.9%, 36% and 80.3%).

*Fitness Cost Hierarchy*

We also explored the impact of removing the assumption of a fitness cost hierarchy on the uncertainty analysis (c_1_ ≈ c_2_ ≈ c_3_ > c_12_ ≈ c_13_ ≈ c_23_ > c_123_). Instead, we allow all fitness cost values (c_x_) to have any value limited by the explored parameter range (Figure S9).


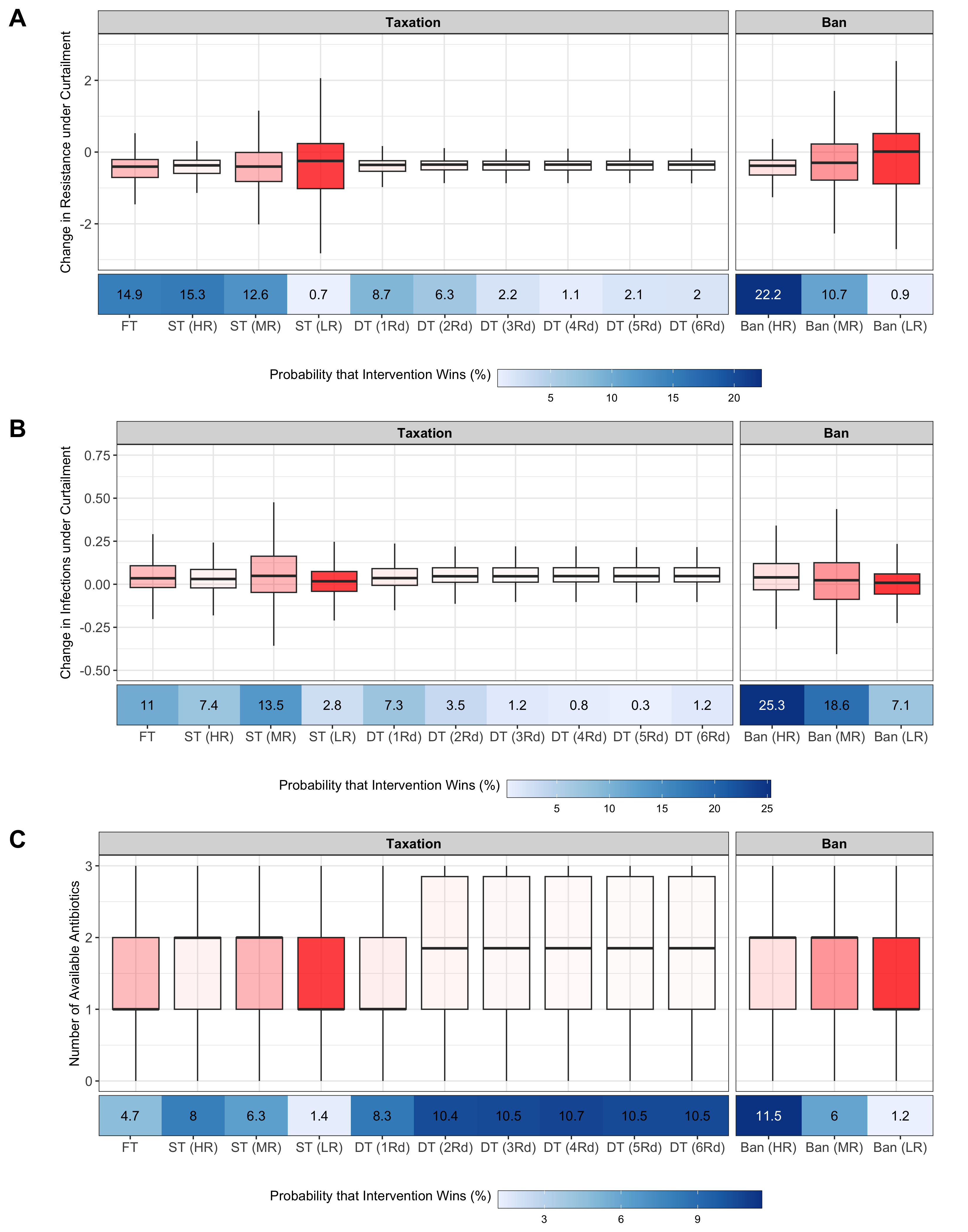


**Figure S9. A) Changes to average resistance under total antibiotic curtailment, B) changes to overall infections under total antibiotic curtailment, C) Number of available antibiotics with a no hierarchy of fitness costs.** FT = Flat Tax, ST = Single Tax, DT = Differential Tax, HR = High Resistance, MR = Medium Resistance and LR = Low Resistance. The intensity of box plot shading represents the proportion of runs resulting in increases to both usage and resistance, representing intervention failure (also used for weighting of intervention performance: 26.5%, 5.1%, 29.1%, 76.1%, 6.8%, 3.8%, 2.8%, 2.2%, 2.0%, 1.8%, 11.6%, 41.1% and 77.9% respectively).

*Alternative Scenarios*

Alternative scenarios were explored for the uncertainty analysis. These can be grouped into an exploration of PED matrix scenarios (high or low compensatory increases in usage), the number of antibiotics explored (2 vs 4), thresholds for what is considered an “available” antibiotic (5%, 10% and 35%) and the extent of taxation (25% and 75%). A comparison of the impact of each scenario on the probability of intervention failure is included (Figure S10).


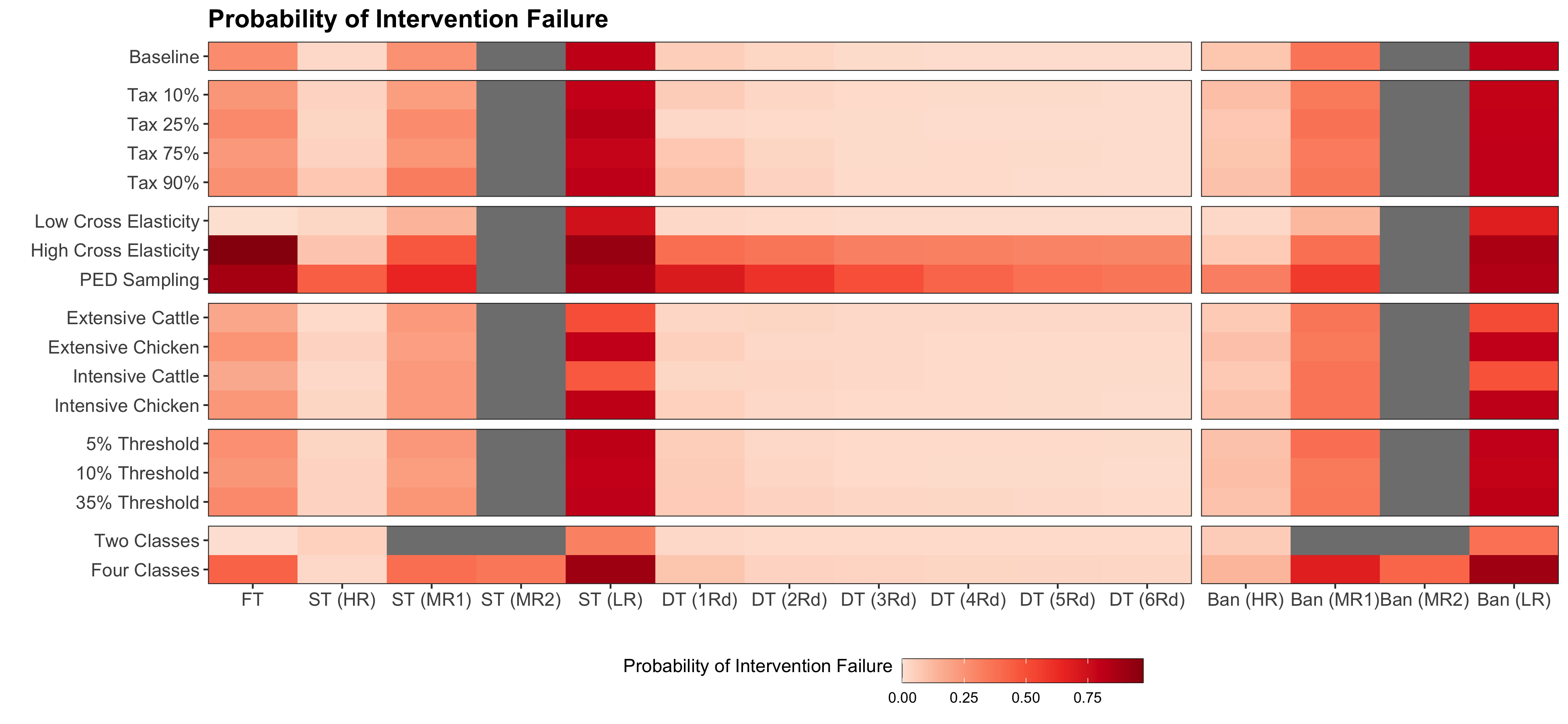


**Figure S10.** **Probability of intervention failure across each of the different alternative scenarios explored.** FT = Flat Tax, ST = Single Tax, DT = Differential Tax, HR = High Resistance, MR1 = 2^nd^ Lowest Resistance, MR2 = 3^rd^ Lowest Resistance, LR = Low Resistance.

The effect size across the three performance criteria and a comparison of the “best performing” intervention was also conducted for each scenario (Figure S10-16).

*PED Matrix Scenarios*

In this study, we considered three sets of PEDs. A baseline PED matrix was chosen with a realistic case study assigning first-line, second-line and last-line identities to the three groups. We also modelled two alternative case studies with unitary elasticity of demand for the own price elasticity of demand and differing cross elasticities of demand (non-diagonal elements). These two scenarios represent, that compensatory increases in usage of other antibiotics will be lower (0.4) or higher (0.6) than decreases to the taxed antibiotic class (eqn S1.6-7).

$$PED=\left[ \begin{matrix} -1 & 0.4 & 0.4 \\ 0.4 & -1 & 0.4 \\ 0.4 & 0.4 & -1 \end{matrix} \right]$$

(eqn S1.6)

$$PED=\left[ \begin{matrix} -1 & 0.6 & 0.6 \\ 0.6 & -1 & 0.6 \\ 0.6 & 0.6 & -1 \end{matrix} \right]$$

(eqn S1.7)

As an illustrative example, we take the first column of the lower compensatory increases PED matrix (eqn 1.2), PED[,1] = [-1, 0.4, 0.4], and identify the change in usage of class 1 antibiotics (σ_1_) assuming a 50% taxation rate: 0.5 x 0.4 + 0.5 x 0.4 + 0.5 x -1 = -10% change in usage. The resulting output from these alternative scenarios can be found below (Figure S11-12).

The PED matrix was also varied in a separate scenario analysis, by treating the cross-price elasticity of demand (0 to 1) and own price elasticity of demand (-2 to 0) as variables which could be sampled uniformly (Figure S13).


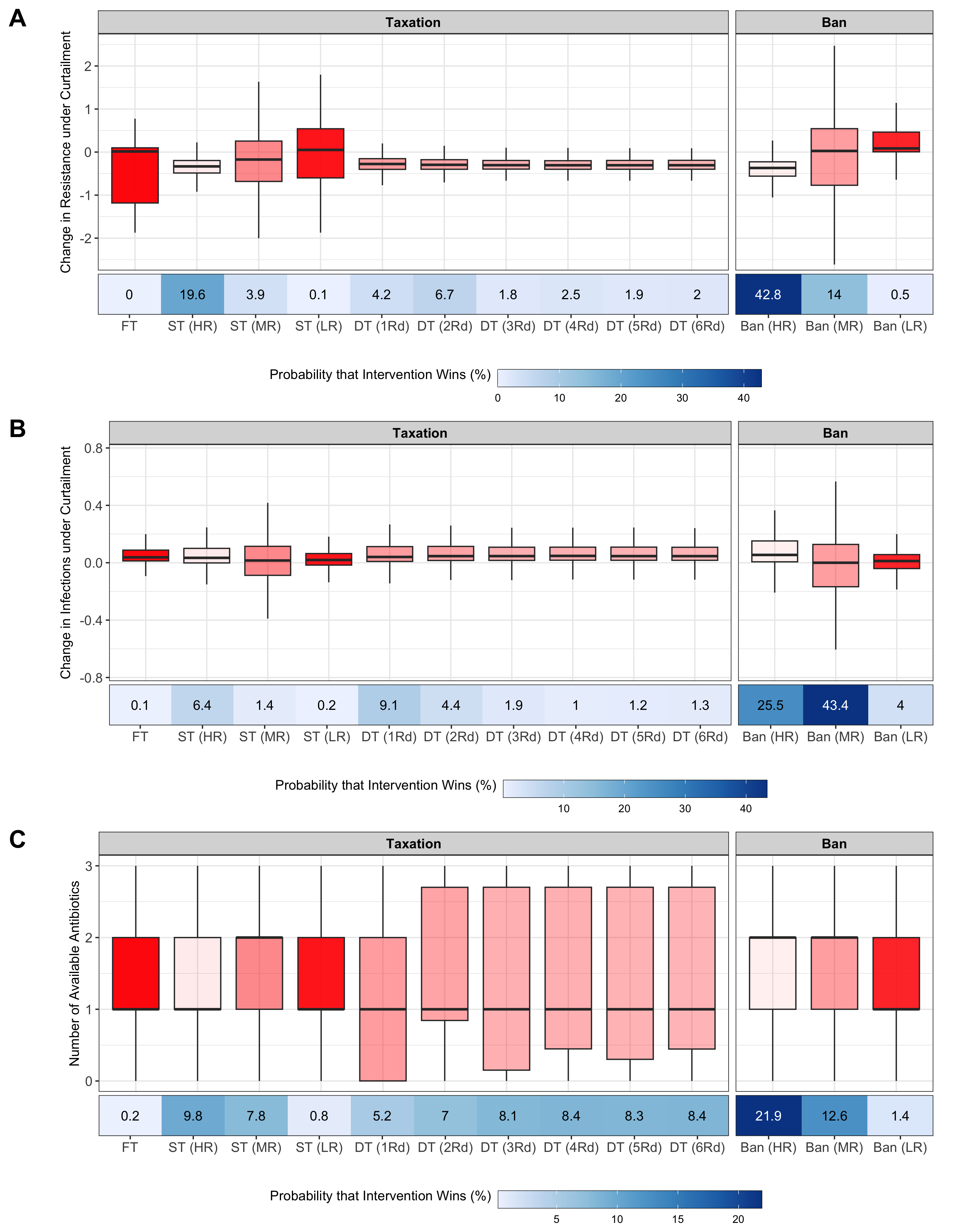


**Figure S11. A) Changes to average resistance under total antibiotic curtailment, B) changes to overall infections under total antibiotic curtailment, C) Number of available antibiotics with a PED matrix modelling severe compensatory increases in usage.** FT = Flat Tax, ST = Single Tax, DT = Differential Tax, HR = High Resistance, MR = Medium Resistance and LR = Low Resistance. The intensity of box plot shading represents the proportion of runs resulting in increases to both usage and resistance, representing intervention failure (also used for weighting of intervention performance: 97.3%, 8.4%, 46.7%, 92.1%, 38.9%, 35.6%, 31.5%, 31.3%, 30.3%, 29.6%, 6.3%, 38% and 86.3% respectively).

*
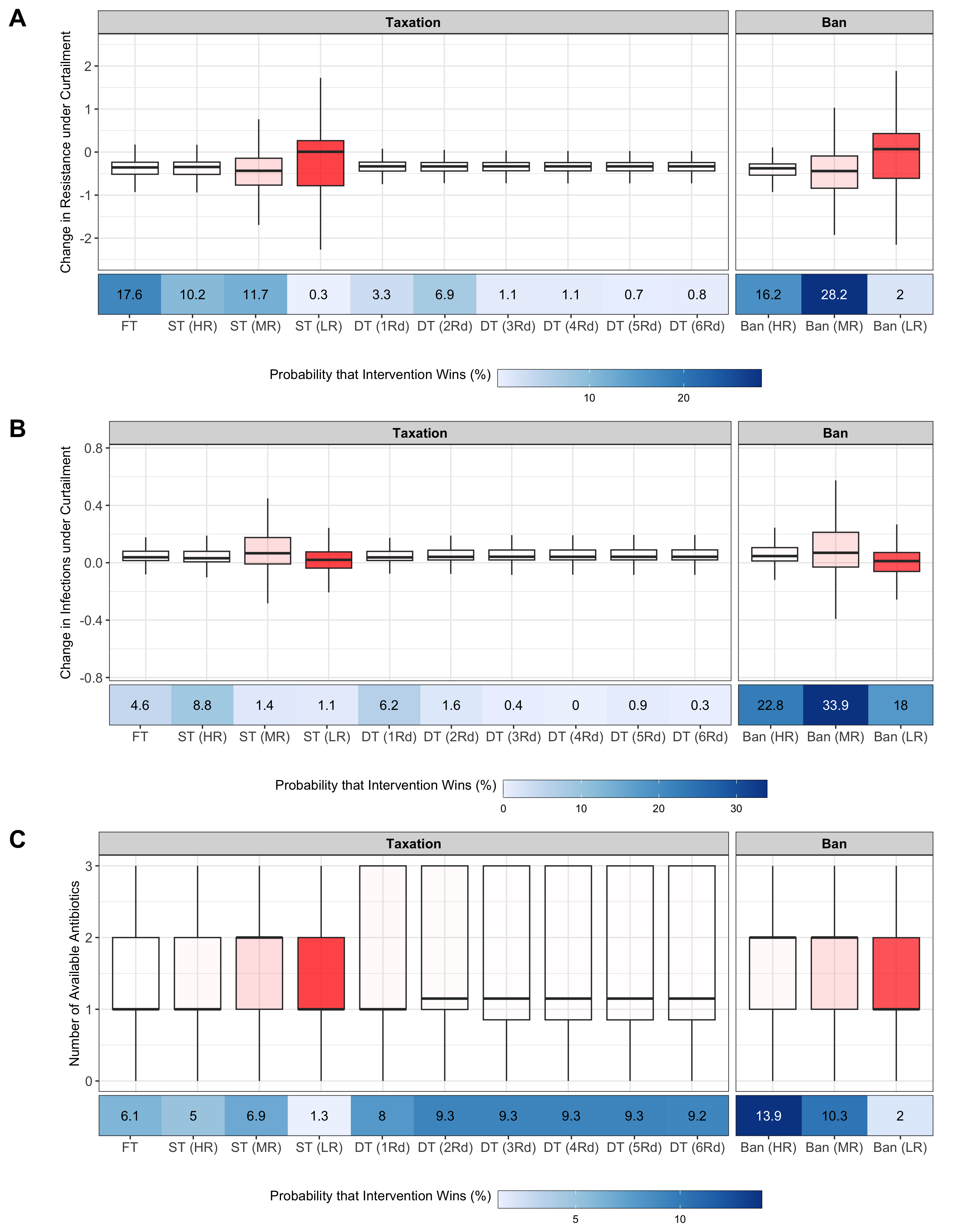
*

**Figure S12. A) Changes to average resistance under total antibiotic curtailment, B) changes to overall infections under total antibiotic curtailment, C) Number of available antibiotics with a PED matrix modelling lower compensatory increases in usage.** FT = Flat Tax, ST = Single Tax, DT = Differential Tax, HR = High Resistance, MR = Medium Resistance and LR = Low Resistance. The intensity of box plot shading represents the proportion of runs resulting in increases to both usage and resistance, representing intervention failure (also used for weighting of intervention performance: 0%, 2.5%, 14%, 74.3%, 2.4%, 1.6%, 0.9%, 0.9%, 0.9%, 0.7%, 2.2%, 12.3% and 68% respectively).


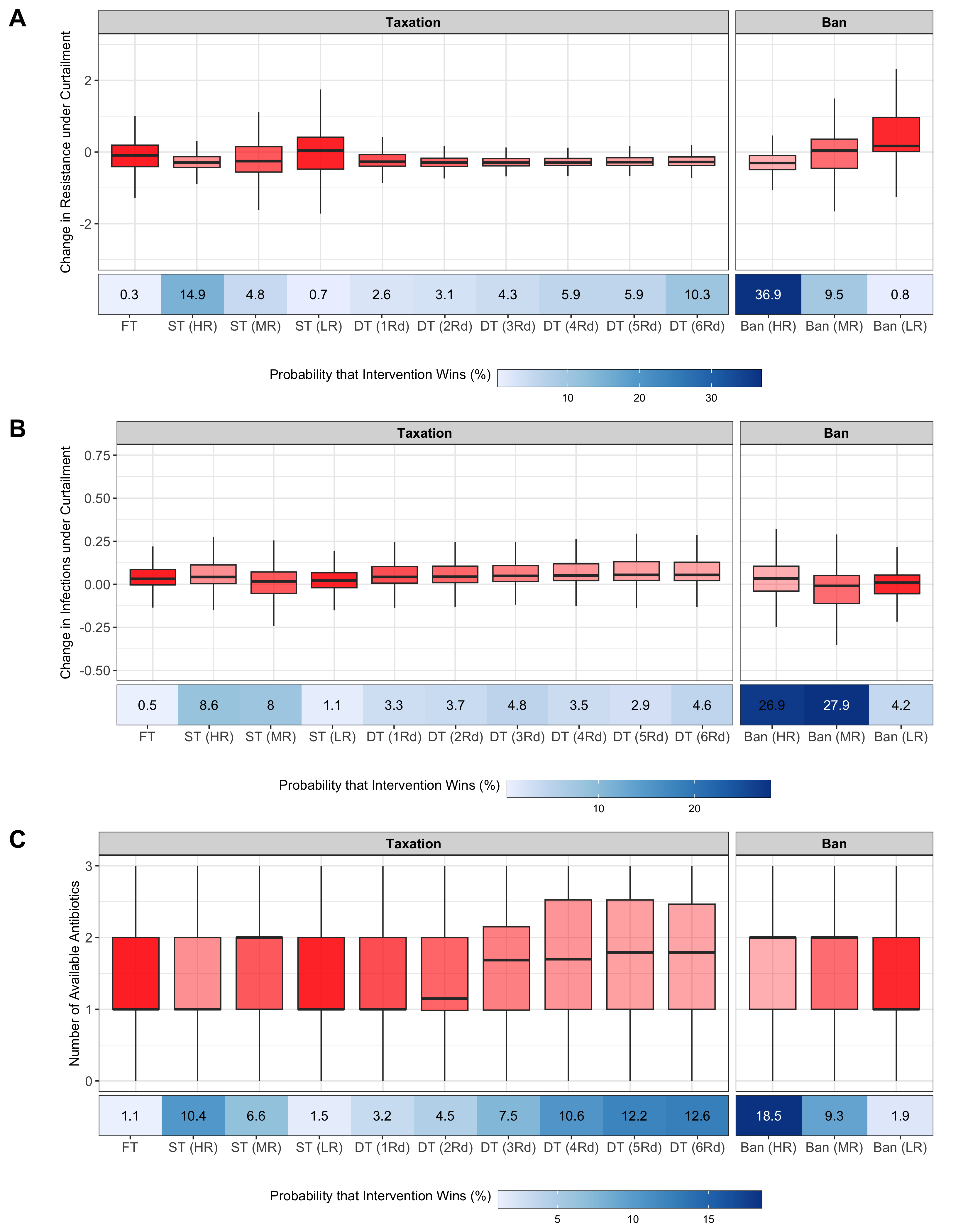


**Figure S13. A) Changes to average resistance under total antibiotic curtailment, B) changes to overall infections under total antibiotic curtailment, C) Number of available antibiotics with a PED matrix that varies.** FT = Flat Tax, ST = Single Tax, DT = Differential Tax, HR = High Resistance, MR = Medium Resistance and LR = Low Resistance. The intensity of box plot shading represents the proportion of runs resulting in increases to both usage and resistance, representing intervention failure (also used for weighting of intervention performance: 88.2%, 44.4%, 65%, 86.7%, 69.7%, 60.2%, 50.3%, 41.9%, 37.7%, 35.5%, 31.9%, 57.7% and 84.4% respectively).

*Number of antibiotics explored*

We additionally explored two scenarios with two and four antibiotic classes explored (Figure S14-15). The primary difference in this scenario, compared to the baseline, was that the baseline for the calculation of differential taxation was changed from the class with medium levels of antibiotic resistance, to the average between the two classes, or the average of the two intermediate resistance classes.

Note that two alternative PED matrices were used for the two scenarios (eqn S1.8-9). These PEDs were formulated to have a similar first-line, second-line etc. PED structure as the matrix used in the baseline example (eqn 1.1).

$$PED=\left[ \begin{matrix} -1.5 & 0.75 \\ 0.5 & -1 \end{matrix} \right]$$

(eqn 1.8)

$$PED=\left[ \begin{matrix} -1.75 & 0.75 & 0.5 & 0.25 \\ 0.5 & -1.5 & 0.75 & 0.5 \\ 0.25 & 0.5 & -1.25 & 0.75 \\ 0 & 0.25 & 0.5 & -1 \end{matrix} \right]$$

(eqn 1.9)

**
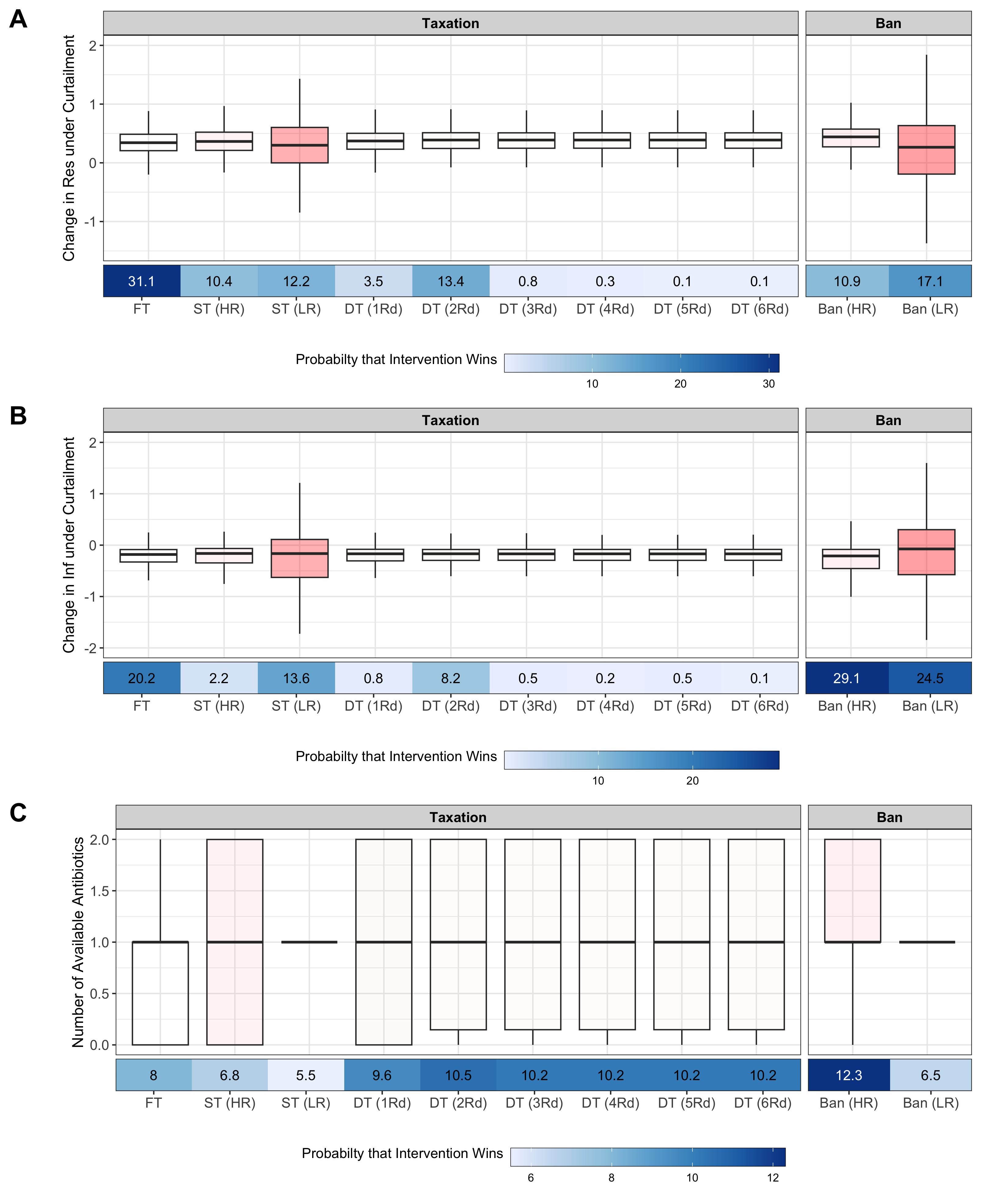
**

**Figure S14. A) Changes to average resistance under total antibiotic curtailment, B) changes to overall infections under total antibiotic curtailment, C) Number of available antibiotics with two antibiotics.** FT = Flat Tax, ST = Single Tax, DT = Differential Tax, HR = High Resistance and LR = Low Resistance. The intensity of box plot shading represents the proportion of runs resulting in increases to both usage and resistance, representing intervention failure (also used for weighting of intervention performance: 0.2%, 4.3%, 31.3%, 1.8%, 1.7%, 1.6%, 1.6%, 1.6%, 1.5%, 5.6% and 37.4% respectively).


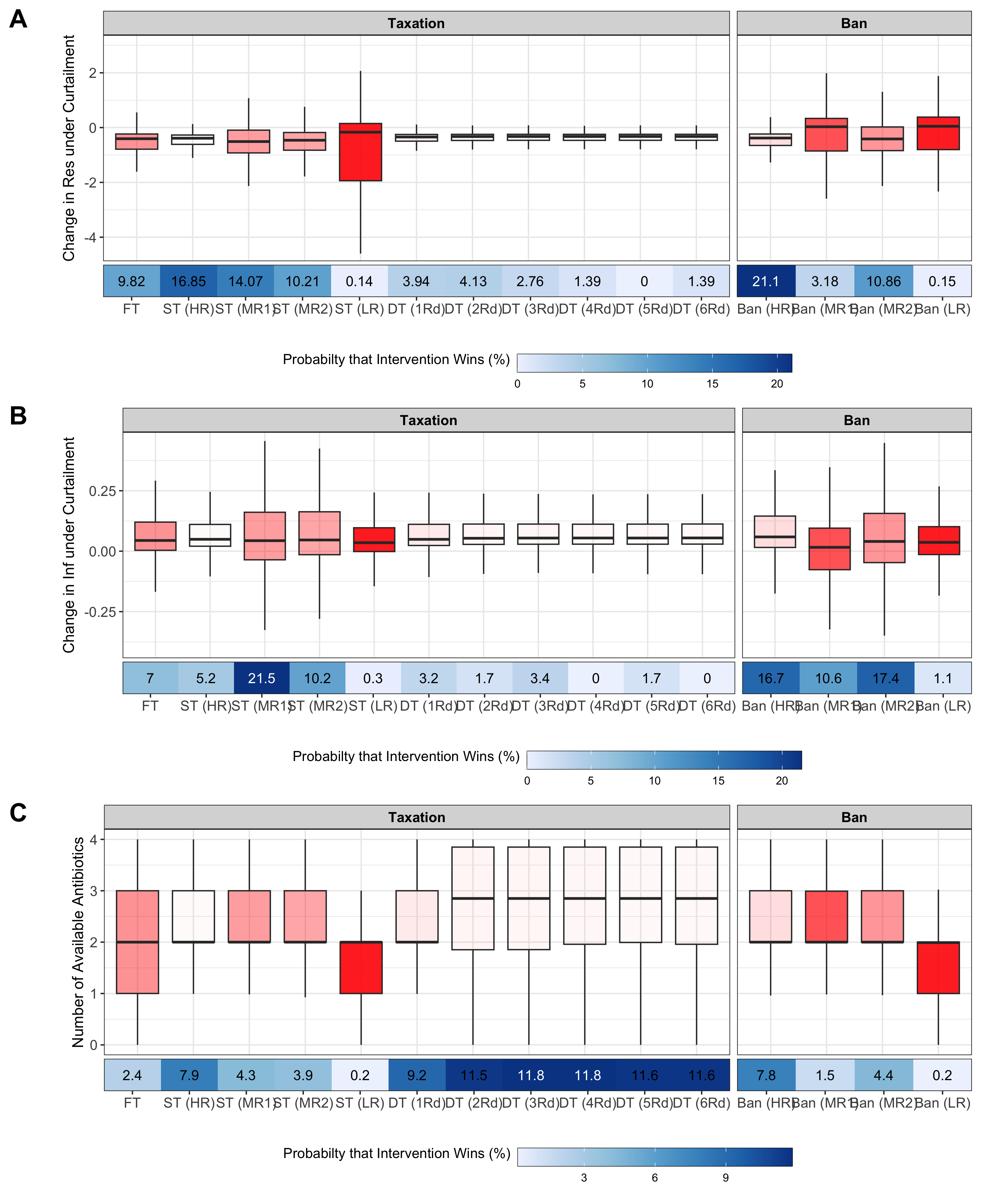


**Figure S15. A) Changes to average resistance under total antibiotic curtailment, B) changes to overall infections under total antibiotic curtailment, C) Number of available antibiotics with four antibiotics.** FT = Flat Tax, ST = Single Tax, DT = Differential Tax, HR = High Resistance, MR1 = 2nd Highest Resistance, MR2 = 2nd Lowest Resistance and LR = Low Resistance. The intensity of box plot shading represents the proportion of runs resulting in increases to both usage and resistance, representing intervention failure (also used for weighting of intervention performance: 42.8%, 1.8%, 38.5%, 35.1%, 90.1%, 8.2%, 3.8%, 3.4%, 3.1%, 2.9%, 2.7%, 13.2%, 68.2%, 41.6% and 89.3% respectively).

*Threshold for an “available antibiotic”*

The baseline for the threshold for an available antibiotic, was considered 25%. This was explored in sensitivity analysis with lower thresholds of 35%, 10% and 5% (Figure S16-18).

*
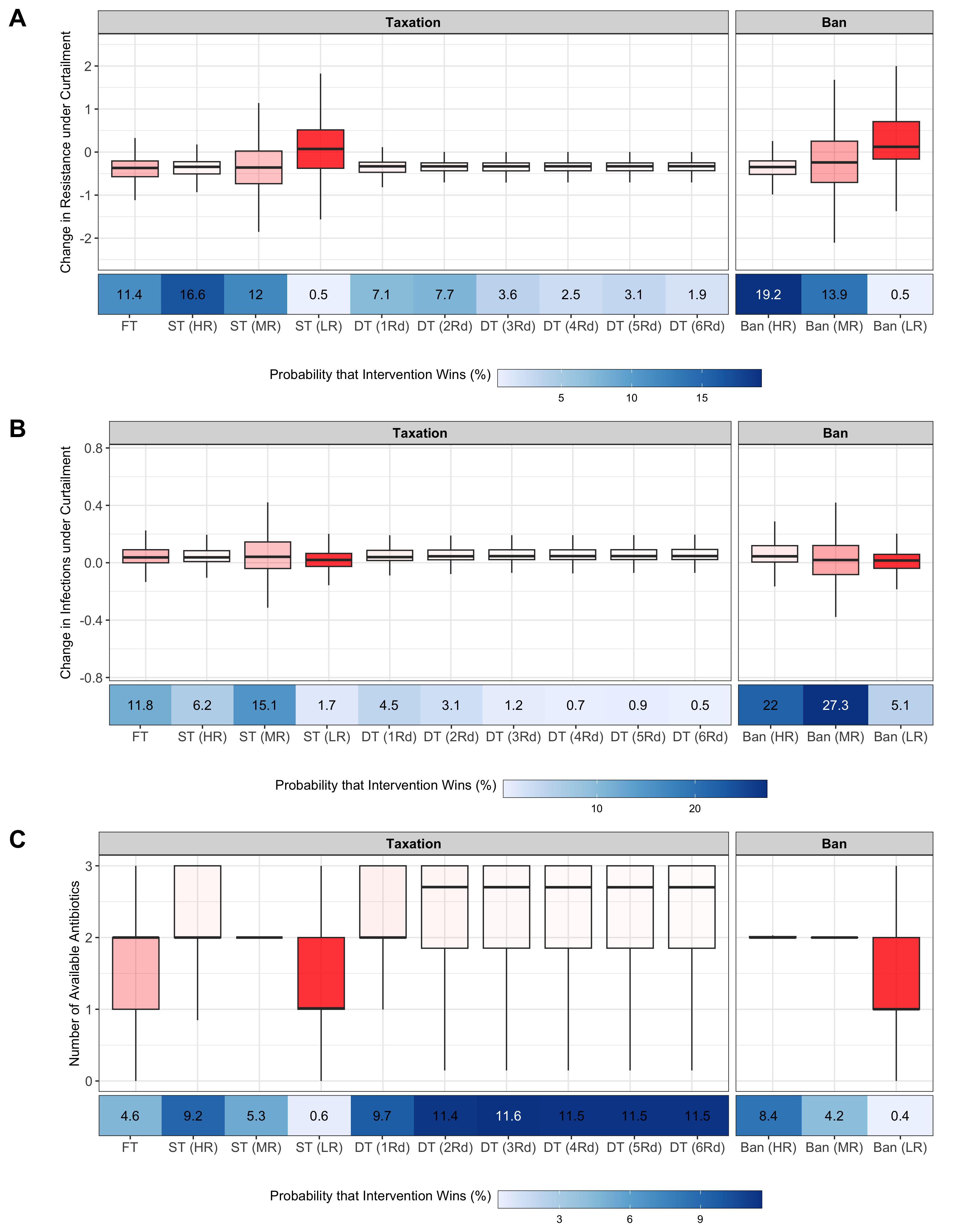
*

**Figure S16. A) Changes to average resistance under total antibiotic curtailment, B) changes to overall infections under total antibiotic curtailment, C) Number of available antibiotics with a 35% threshold for an “available antibiotic”.** FT = Flat Tax, ST = Single Tax, DT = Differential Tax, HR = High Resistance and LR = Low Resistance. The intensity of box plot shading represents the proportion of runs resulting in increases to both usage and resistance, representing intervention failure (also used for weighting of intervention performance: 28.3%, 3.8%, 24.4%, 80.5%, 6%, 3.9% 2.6%, 2.5%, 2.2%, 1.7%, 8.8%, 34.5% and 80.7% respectively).

*
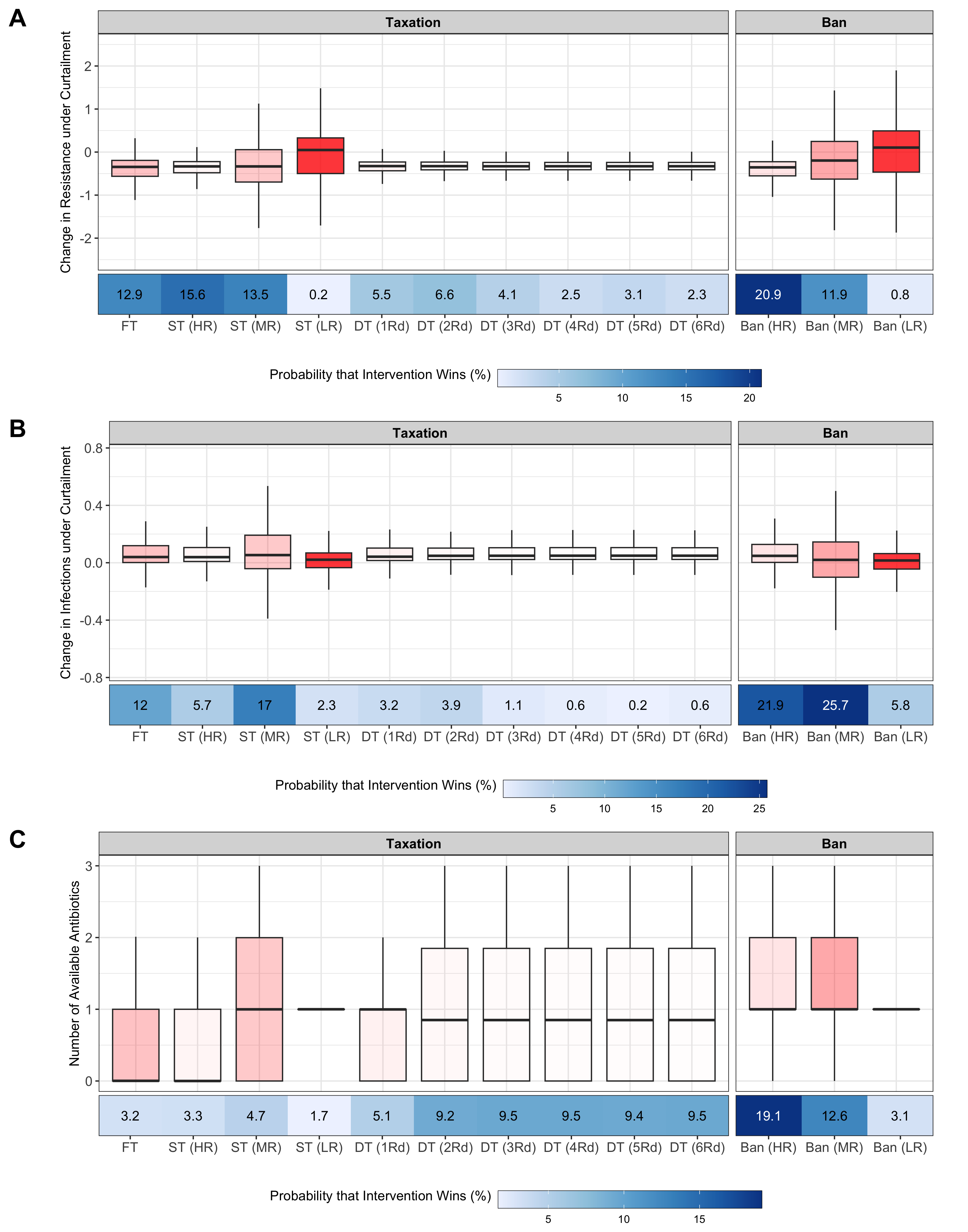
*

**Figure S17. A) Changes to average resistance under total antibiotic curtailment, B) changes to overall infections under total antibiotic curtailment, C) Number of available antibiotics with a 10% threshold for an “available antibiotic”.** FT = Flat Tax, ST = Single Tax, DT = Differential Tax, HR = High Resistance and LR = Low Resistance. The intensity of box plot shading represents the proportion of runs resulting in increases to both usage and resistance, representing intervention failure (also used for weighting of intervention performance: 24%, 3.9% 21.2% 79.4% 5.6% 2.6% 1.7% 1.3% 1.1% 0.9% 10.4% 34% and 78.9% respectively).

*
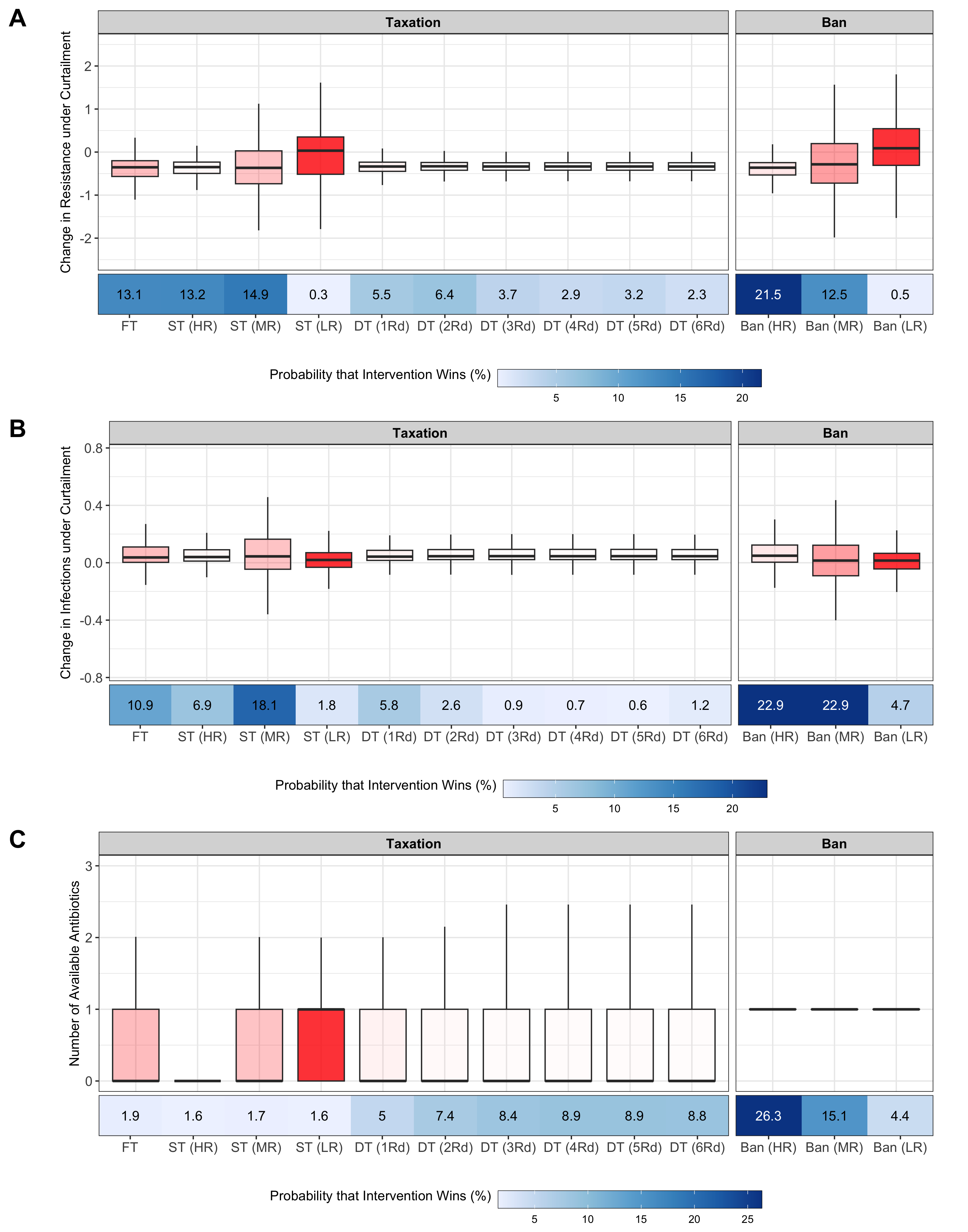
*

**Figure S18. A) Changes to average resistance under total antibiotic curtailment, B) changes to overall infections under total antibiotic curtailment, C) Number of available antibiotics with a 35% threshold for an “available antibiotic”.** FT = Flat Tax, ST = Single Tax, DT = Differential Tax, HR = High Resistance and LR = Low Resistance. The intensity of box plot shading represents the proportion of runs resulting in increases to both usage and resistance, representing intervention failure (also used for weighting of intervention performance: 28.3%, 3.8%, 24.4%, 80.5%, 6%, 3.9%, 2.6%, 2.5%, 2.2%, 1.7%, 8.8%, 34.5% and 80.7% respectively).

*Taxation Rate*

The baseline taxation rate for all interventions was 50% (a 50% increase in the price of veterinary antibiotics). This was also explored in scenario analysis with 10%, 25%, 75% and 90% taxation rates (Figure S19-22).


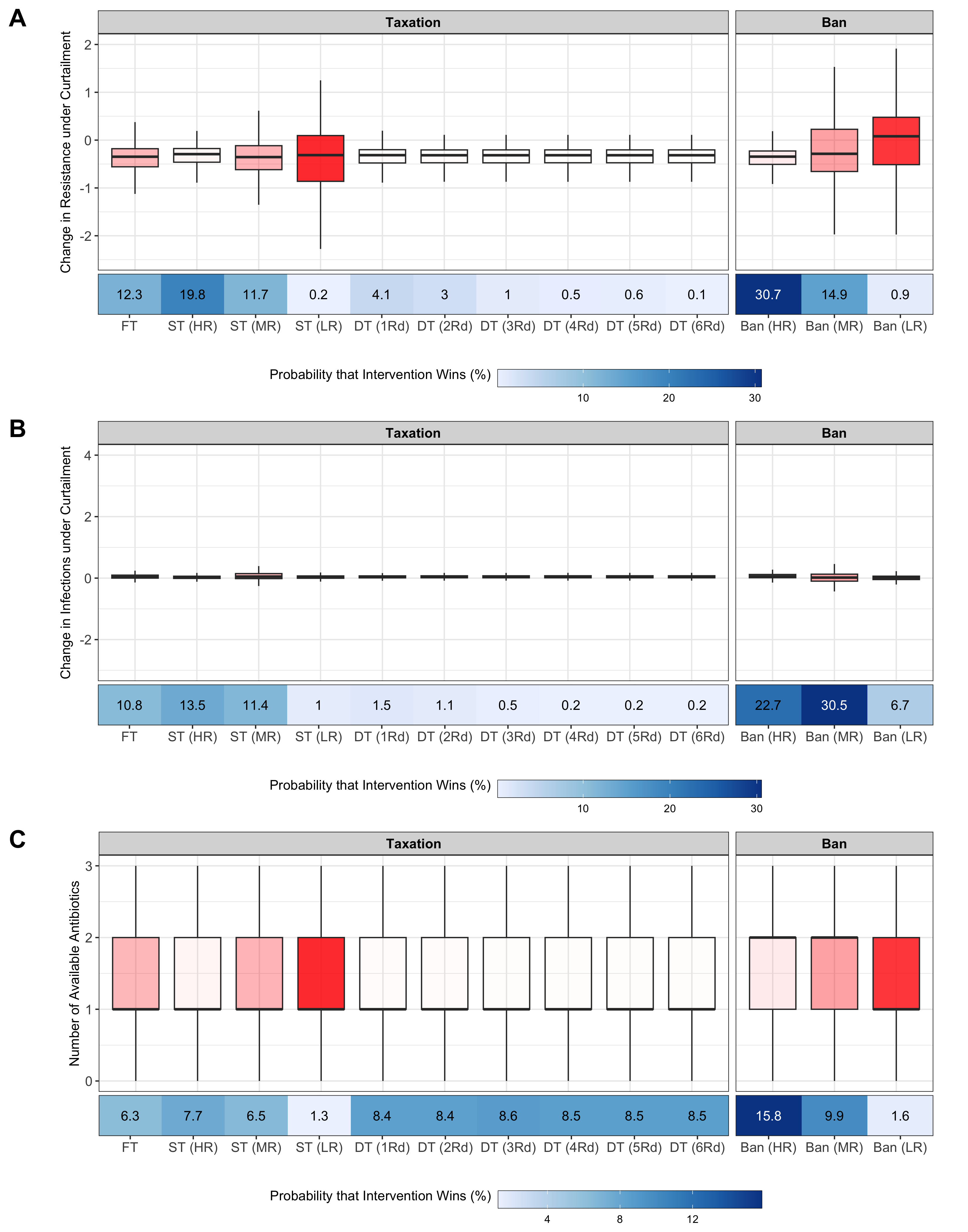


**Figure S19. A) Changes to average resistance under total antibiotic curtailment, B) changes to overall infections under total antibiotic curtailment, C) Number of available antibiotics with a 10% taxation rate.** FT = Flat Tax, ST = Single Tax, DT = Differential Tax, HR = High Resistance and LR = Low Resistance. The intensity of box plot shading represents the proportion of runs resulting in increases to both usage and resistance, representing intervention failure (also used for weighting of intervention performance: 28.1%, 4.0%, 29.3%, 83.9%, 1.6%, 1.5%, 1.2%, 1.1%, 1.1%, 1.1%, 8.2%, 36.7% and 78.8% respectively).

*
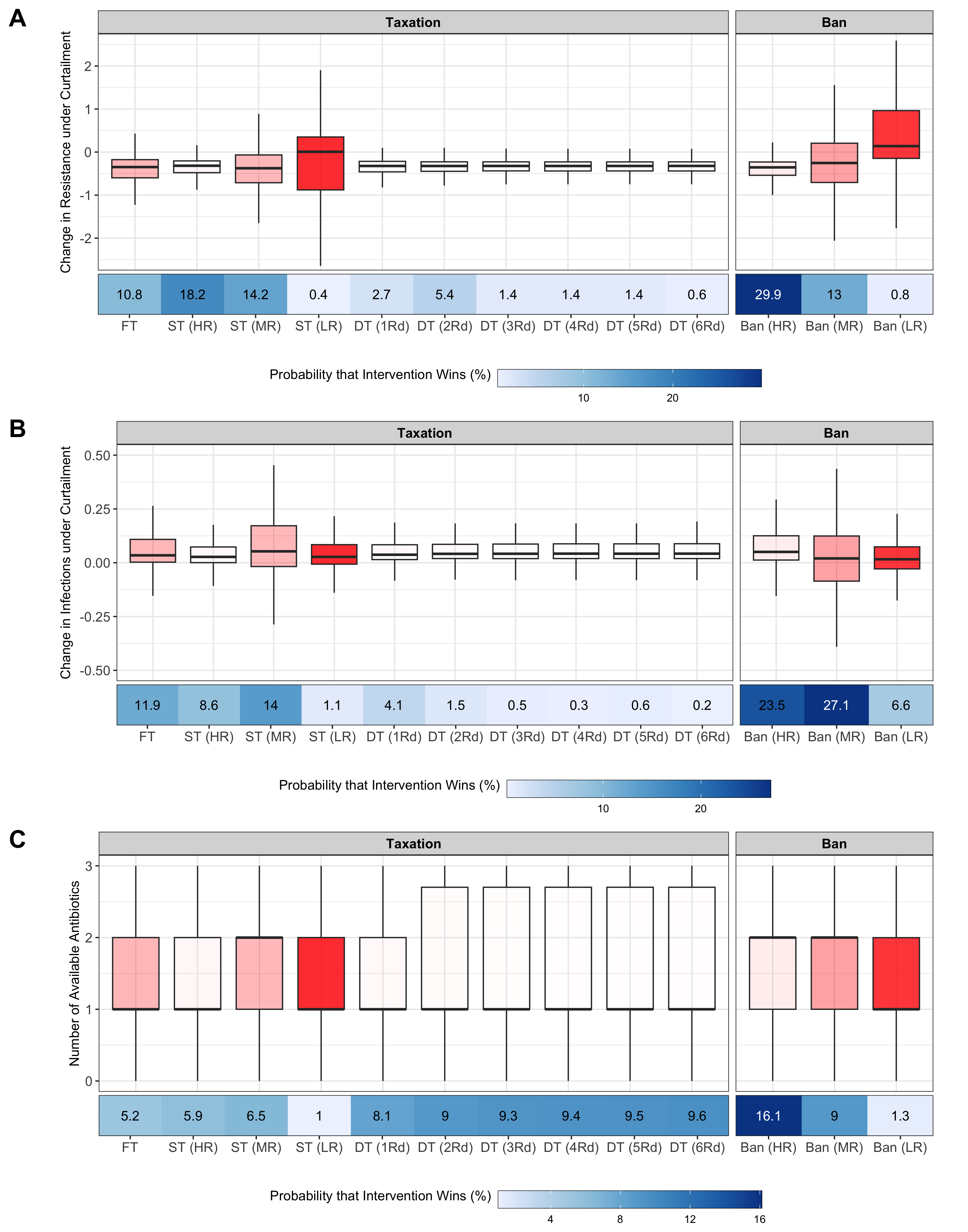
*

**Figure S20. A) Changes to average resistance under total antibiotic curtailment, B) changes to overall infections under total antibiotic curtailment, C) Number of available antibiotics with a 25% taxation rate.** FT = Flat Tax, ST = Single Tax, DT = Differential Tax, HR = High Resistance and LR = Low Resistance. The intensity of box plot shading represents the proportion of runs resulting in increases to both usage and resistance, representing intervention failure (also used for weighting of intervention performance: 28.3%, 3.8%, 24.4%, 80.5%, 6%, 3.9%, 2.6%, 2.5%, 2.2%, 1.7%, 8.8%, 34.5% and 80.7% respectively).

*
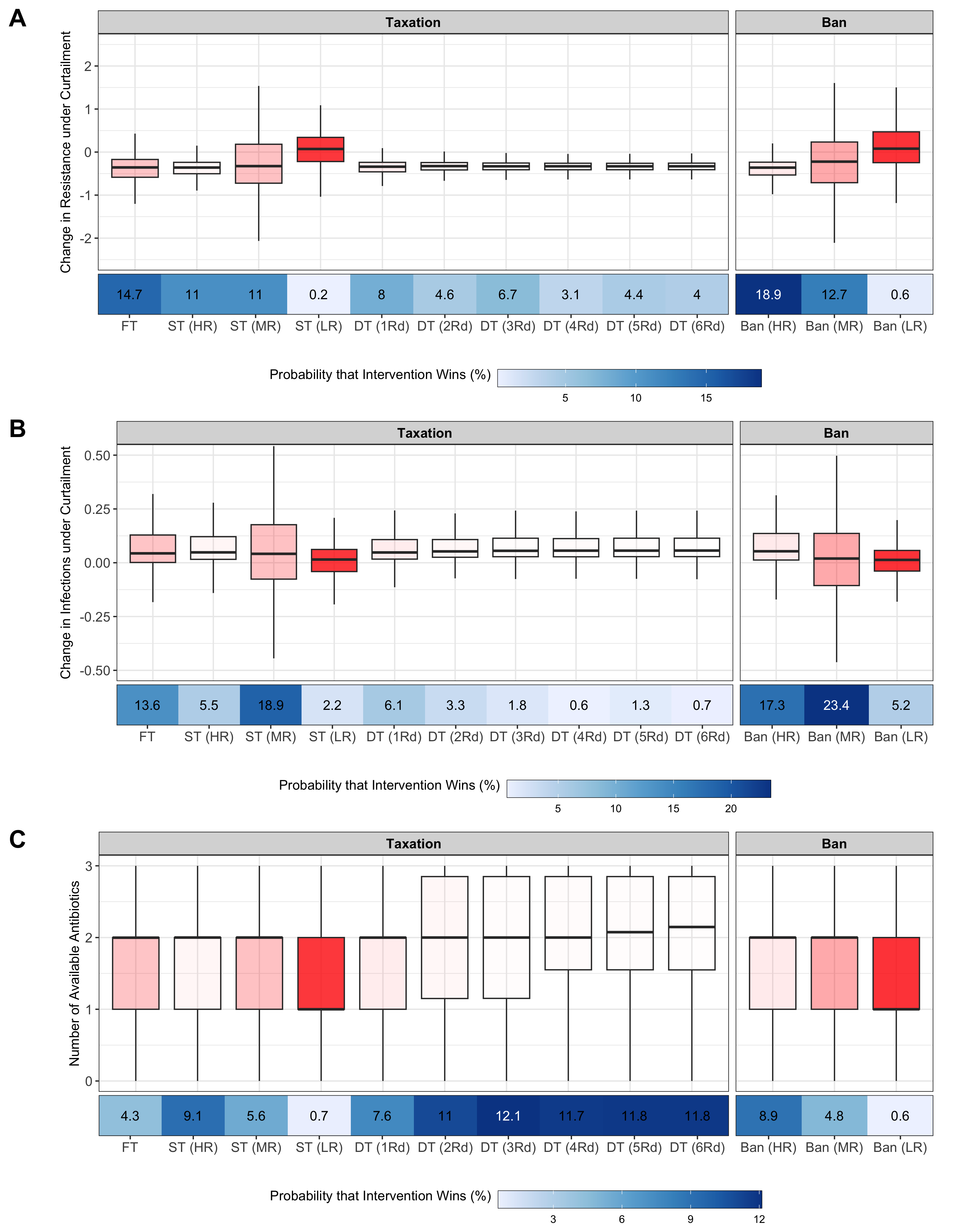
*

**Figure S21. A) Changes to average resistance under total antibiotic curtailment, B) changes to overall infections under total antibiotic curtailment, C) Number of available antibiotics with a 75% taxation rate.** FT = Flat Tax, ST = Single Tax, DT = Differential Tax, HR = High Resistance and LR = Low Resistance. The intensity of box plot shading represents the proportion of runs resulting in increases to both usage and resistance, representing intervention failure (also used for weighting of intervention performance: 28.3%, 3.8%, 24.4%, 80.5%, 6%, 3.9%, 2.6%, 2.5%, 2.2%, 1.7%, 8.8%, 34.5% and 80.7% respectively).


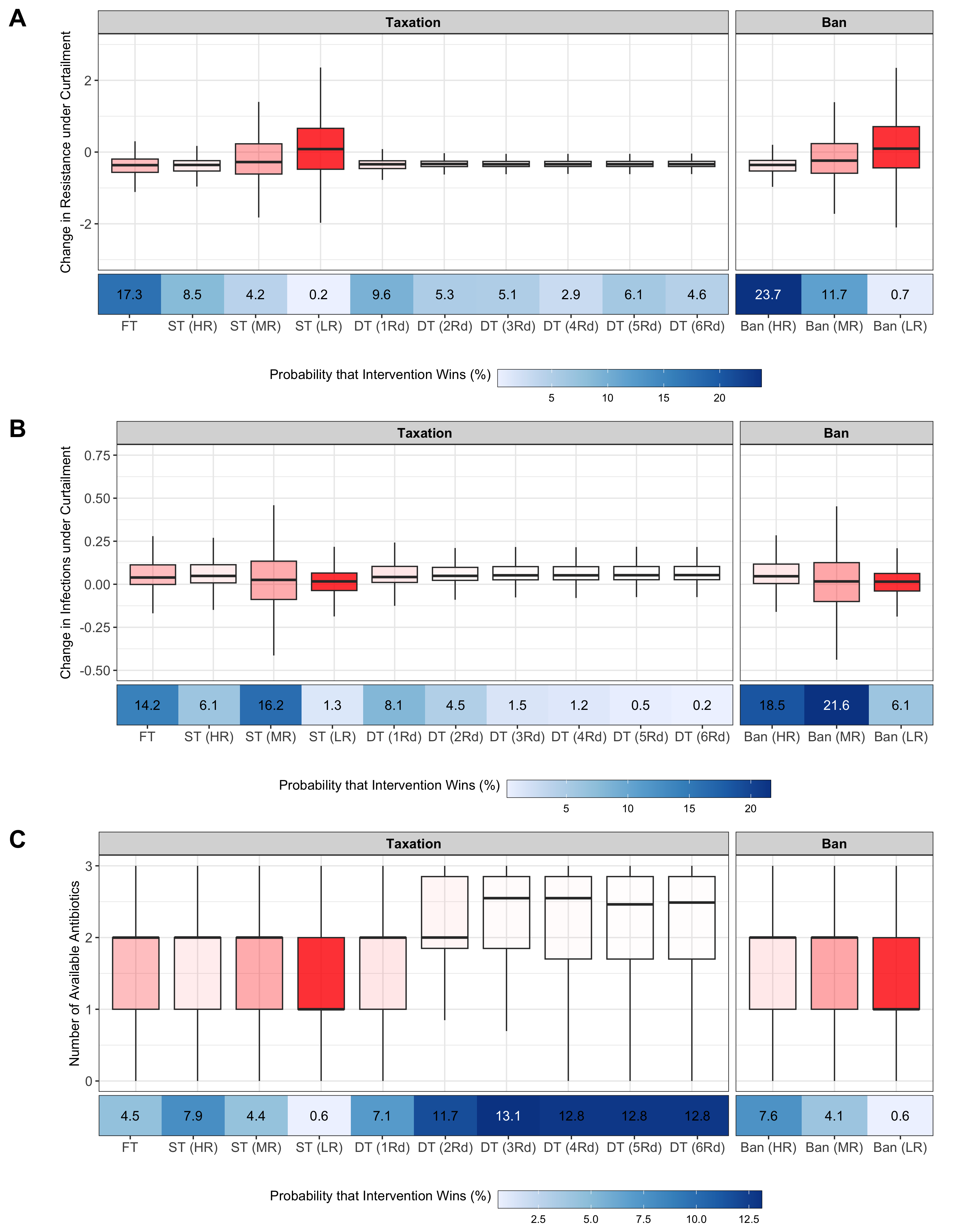


**Figure S22. A) Changes to average resistance under total antibiotic curtailment, B) changes to overall infections under total antibiotic curtailment, C) Number of available antibiotics with a 90% taxation rate.** FT = Flat Tax, ST = Single Tax, DT = Differential Tax, HR = High Resistance and LR = Low Resistance. The intensity of box plot shading represents the proportion of runs resulting in increases to both usage and resistance, representing intervention failure (also used for weighting of intervention performance: 25.9%, 7.4%, 33%, 81%, 9.8%, 3.9%, 1.5%, 1.5%, 0.9%, 0.7%, 9.1%, 35%, and 80.4% respectively).

*Case Study of Livestock Production System*

Specific case studies of livestock species and production system were also included. This included cattle (*λ* = 365^-1^ days) vs broiler chicken (*λ* = 42^-1^ days) livestock species, varying the birth/death rate for each species and keeping this value static in the uncertainty analysis.

Extensive vs intensive production systems were also considered. This was modelled by assuming a “best” and “worst” case scenario for each production system respectively. With extensive production assumed to have an order of magnitude lower transmission when compared to baseline parameterisation (*β* = 0.5), and intensive production assumed to have an order of magnitude higher than baseline (*β* = 50) (Figure S23-26).


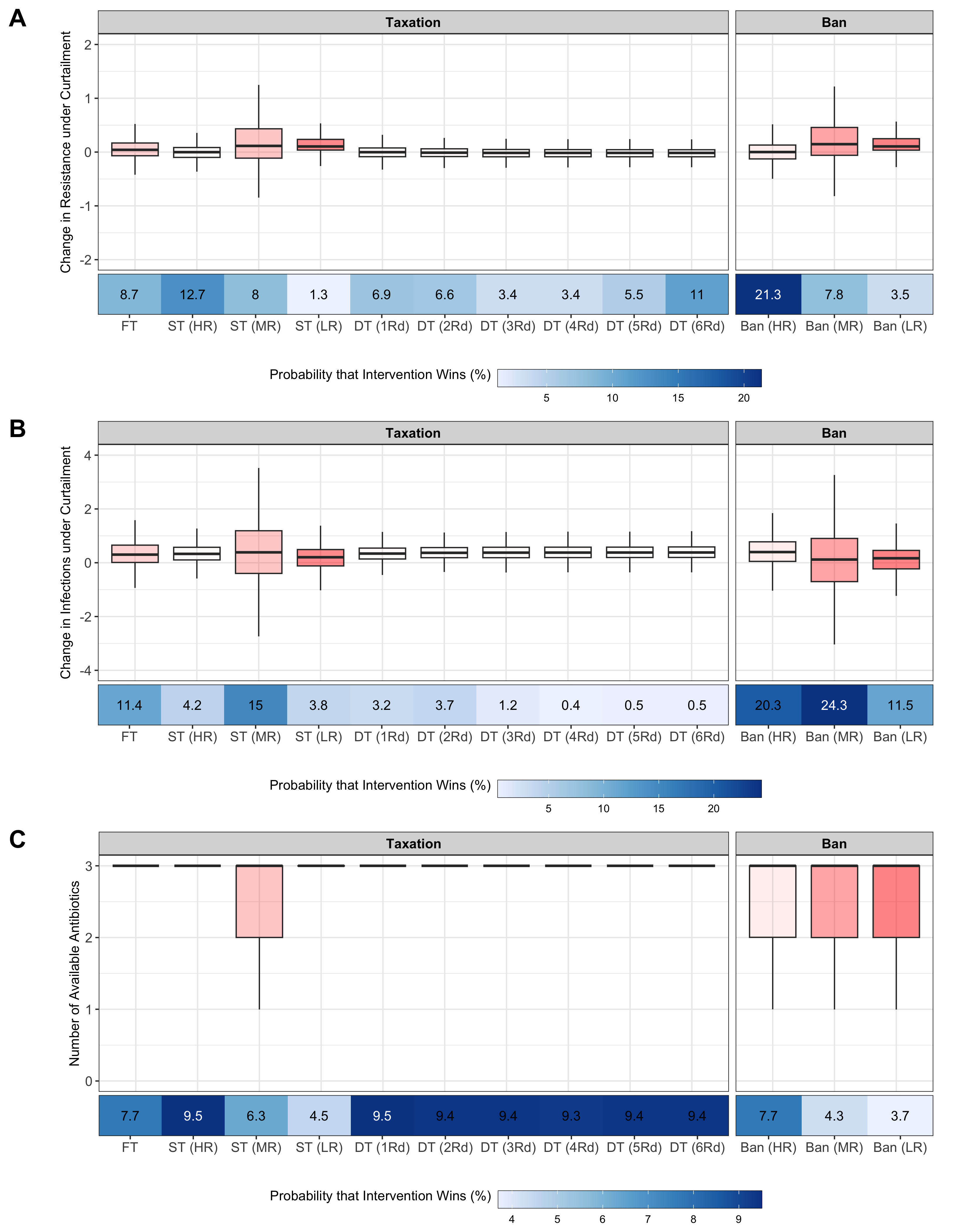


**Figure S23. A) Changes to average resistance under total antibiotic curtailment, B) changes to overall infections under total antibiotic curtailment, C) Number of available antibiotics with a broiler chicken (*λ* = 42^-1^ days) in extensive (*β* = 0.5) production system case study.** FT = Flat Tax, ST = Single Tax, DT = Differential Tax, HR = High Resistance and LR = Low Resistance. The intensity of box plot shading represents the proportion of runs resulting in increases to both usage and resistance, representing intervention failure (also used for weighting of intervention performance: 17.4%, 1.8%, 22.8%, 46.4%, 2.5%, 2.7%, 1.8%, 1.7%, 1.3%, 1.1%, 7.0%, 35.9% and 49.1% respectively).


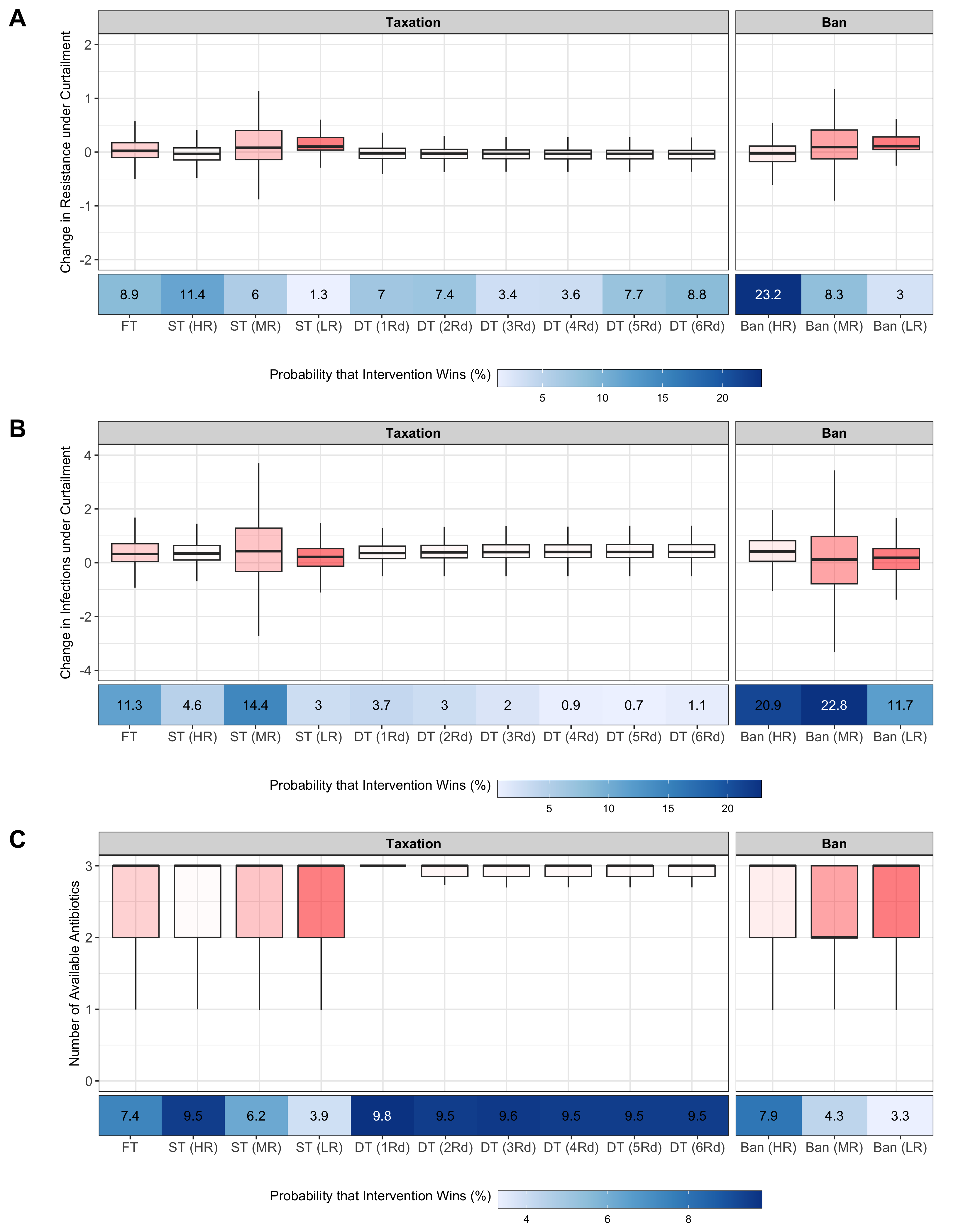


**Figure S24. A) Changes to average resistance under total antibiotic curtailment, B) changes to overall infections under total antibiotic curtailment, C) Number of available antibiotics with a cattle (*λ* = 365^-1^ days) in extensive (*β* = 0.5) production system case study.** FT = Flat Tax, ST = Single Tax, DT = Differential Tax, HR = High Resistance and LR = Low Resistance. The intensity of box plot shading represents the proportion of runs resulting in increases to both usage and resistance, representing intervention failure (also used for weighting of intervention performance: 18.2%, 1.6%, 22.7%, 50.5%, 2.6%, 3.1%, 2.2%, 2.2%, 2.0%, 1.9%, 6.5%, 35.6% and 51.4% respectively).


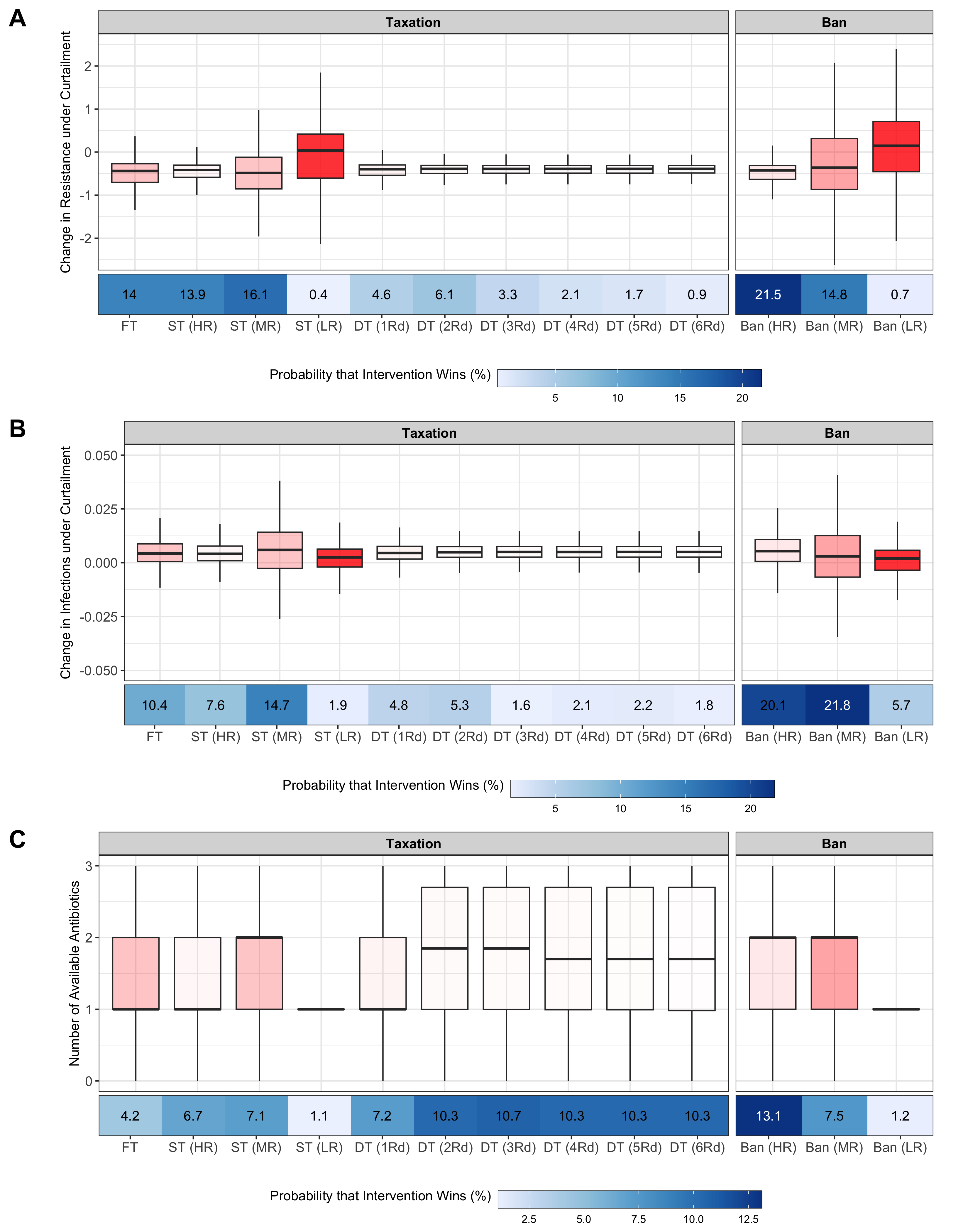


**Figure S25. A) Changes to average resistance under total antibiotic curtailment, B) changes to overall infections under total antibiotic curtailment, C) Number of available antibiotics with a chicken (*λ* = 42^-1^ days) in intensive (*β* = 50) production system case study.** FT = Flat Tax, ST = Single Tax, DT = Differential Tax, HR = High Resistance and LR = Low Resistance. The intensity of box plot shading represents the proportion of runs resulting in increases to both usage and resistance, representing intervention failure (also used for weighting of intervention performance: 23.6%, 3.1%, 22.8%, 80.8%, 4.4%, 2.0%, 1.5%, 1.5%, 1.2%, 0.9%, 8.9%, 35.7% and 81.0% respectively).


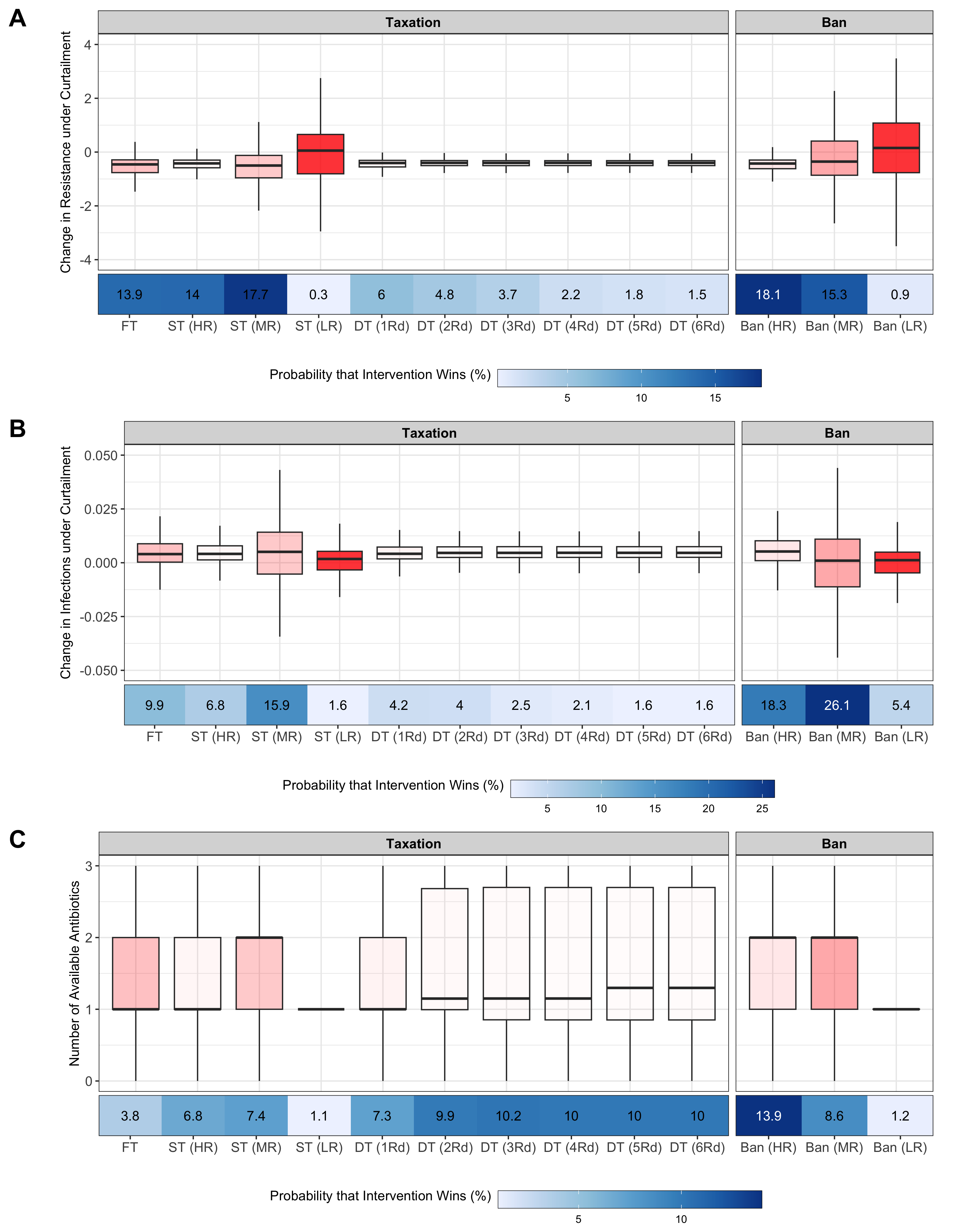


**Figure S26. A) Changes to average resistance under total antibiotic curtailment, B) changes to overall infections under total antibiotic curtailment, C) Number of available antibiotics with a cattle (*λ* = 365^-1^ days) in intensive (*β* = 50) production system case study.** FT = Flat Tax, ST = Single Tax, DT = Differential Tax, HR = High Resistance and LR = Low Resistance. The intensity of box plot shading represents the proportion of runs resulting in increases to both usage and resistance, representing intervention failure (also used for weighting of intervention performance: 24.8%, 3.7%, 21.1% 80.2%, 4.7%, 2.4%, 1.8%, 1.6%, 1.5%, 1.4%, 9.6%, 34.0% and 80.0% respectively).

*Baseline Run, Average Resistance, Overall Infections and Usage Trajectory Plots*

As a supplementary analysis we also include trajectory plots for the three antibiotic classes, average resistance and overall infections (both drug sensitive and resistant) over the course of the model simulation (Figure S27-28). We also include trajectory plots identifying the changes in overall usage (σ_1_ + σ_2_ + σ_3_) over the course of the model simulation (Figure S29). All supplementary trajectory plots were parameterised identically to the baseline plots in Figure 2.

**
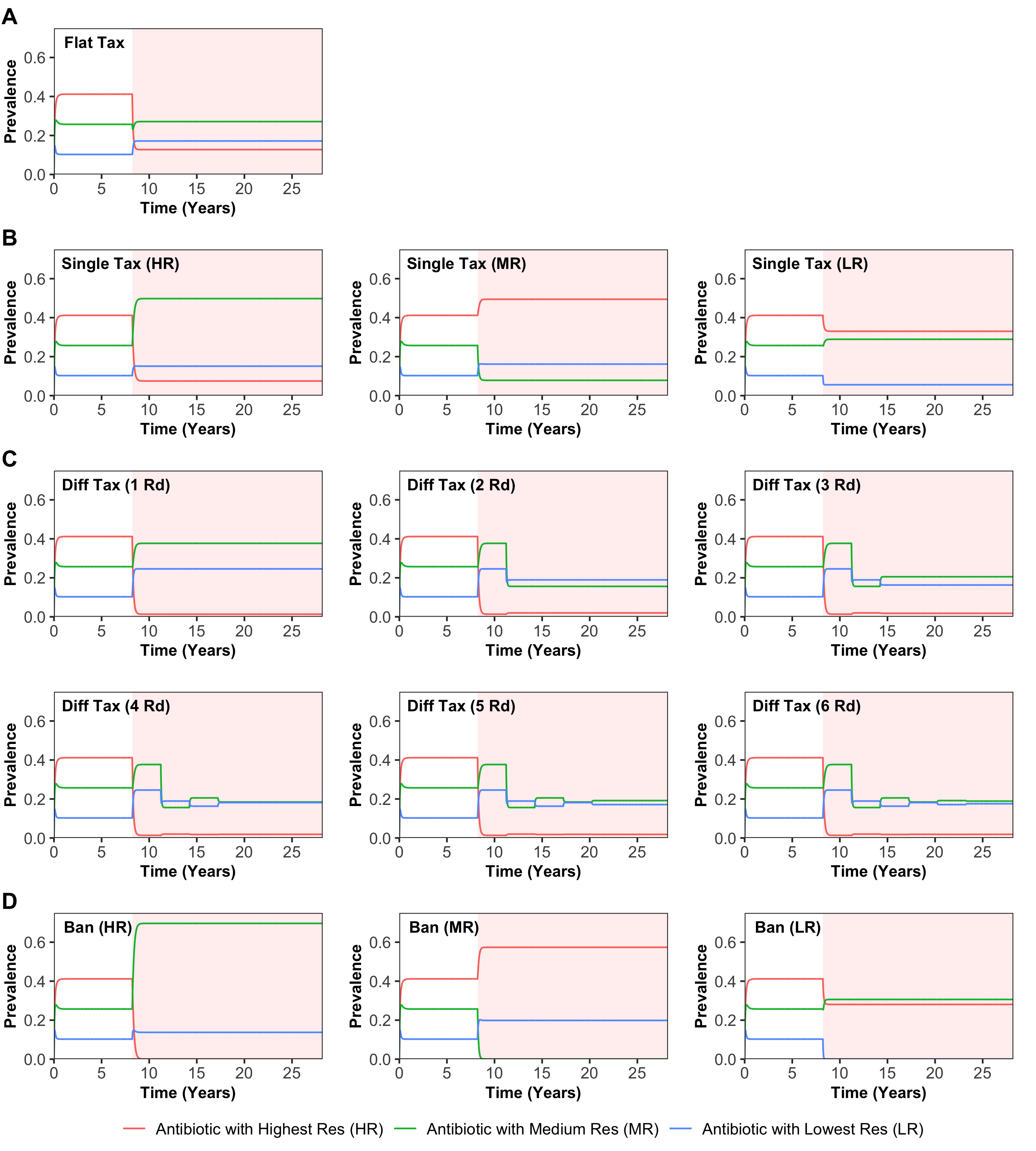
**

**Figure S27. Dynamics of resistance in a food animal population for three antibiotic classes under flat, single, differential taxation schemes, and bans on antibiotic usage.** HR = High Resistance, MR = Medium Resistance and LR = Low Resistance.


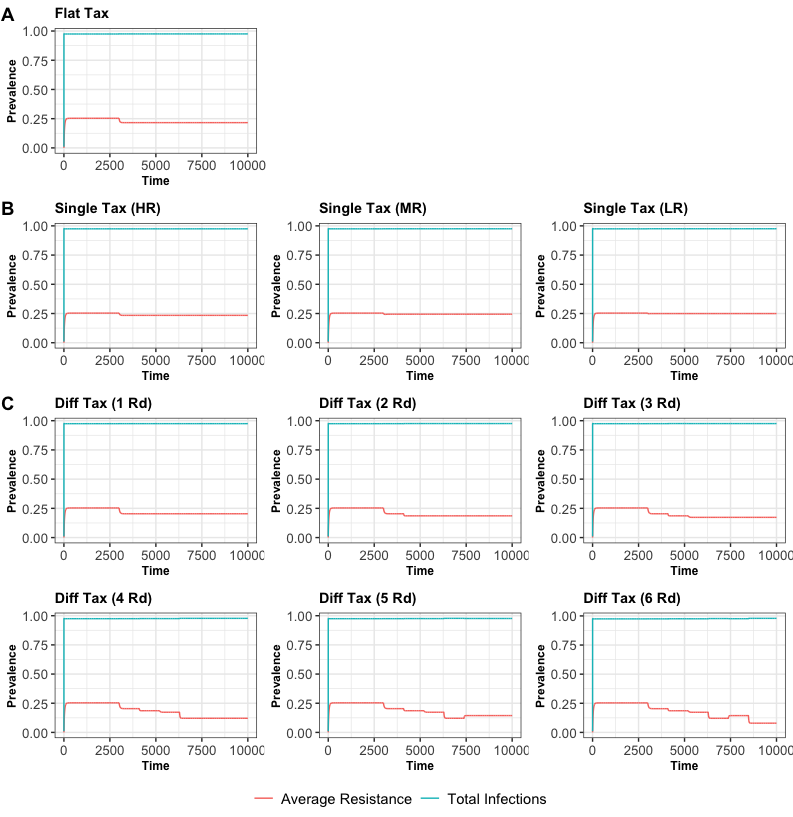


**Figure S28. Trajectory plots describing the dynamics of average resistance and total infections in a livestock population for flat, single and differential taxation schemes.** HR = High Resistance, MR = Medium Resistance and LR = Low Resistance.


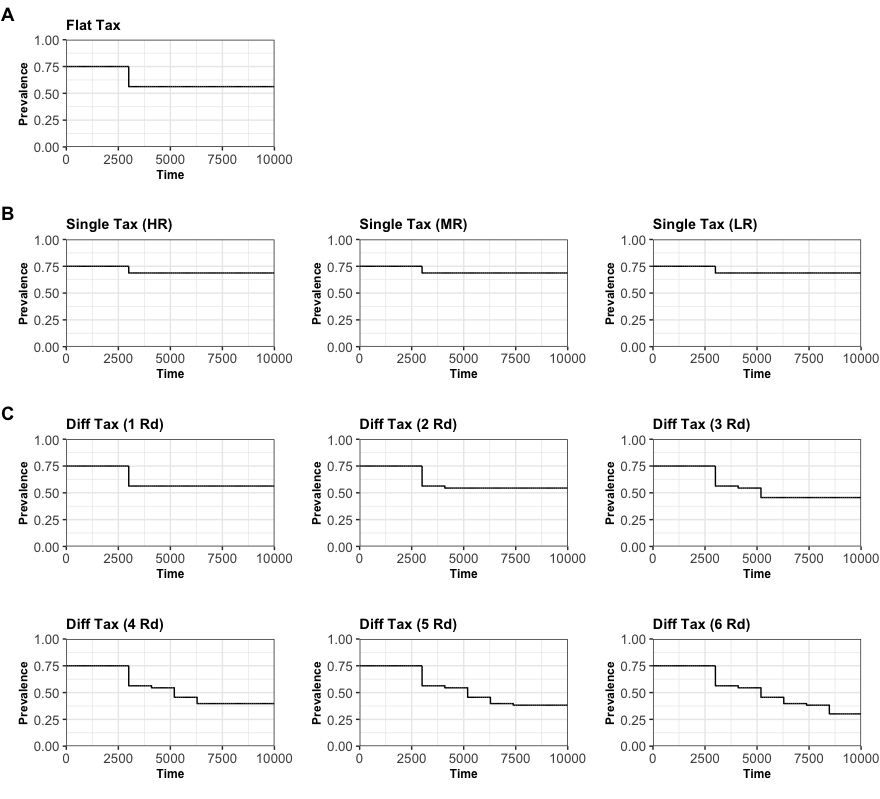


**Figure S29. Trajectory plots describing the change in overall antibiotic usage in a livestock population under flat, single and differential taxation schemes.** HR = High Resistance, MR = Medium Resistance LR = Low Resistance.

*Estimated Revenue from Taxation*

This study used two case studies, high-income countries (HICs) and Lower-Middle Income Countries (LMICs), to estimate the projected yearly revenue from each taxation strategy. The first step was to estimate the yearly projected revenue from veterinary antibiotic sales from each country belonging to HICs or LMICs. Note that the yearly revenue (before taxation) was scaled so that the modelled revenue would match with the estimated market value for veterinary antibiotics identified from literature, estimated at an average value of $2.731 billion [4].

This required information on the total kilograms of active ingredient and the price per kilogram of active ingredient across each antibiotic class.

Countries were first grouped into HIC (HIC) or LMIC (Aggregating LIC, LMIC and UMIC using World Bank classifications) [5]. A global estimation for antibiotic sales in each group was obtained from a global estimation of antibiotic sales of veterinary antibiotics [6]. US and Chinese prices for each antibiotic class were obtained from online retailers (valleyvet.com and alibaba.com) and used as proxies for the price of antibiotics for HICs and LMICs respectively (Table S5-6). Chinese prices were converted from RMB to USD using a flat conversion scaling factor of 1 RMB to $0.15. If multiple antibiotic products were available for each antibiotic class, the average price across all products was taken.

**Table S5. US pricing, aggregated sales data for HICs, and estimated yearly revenue from antibiotics in HICs.**

| **Class** | **Name** | **Size (mL)** | **mg/mL** | **mg/Purchase** | **Price ($)** | **Price per Kilo ($)** | **Average Price ($)** | **Sales HIC**  **(tonnes)** | **Total Yearly Revenue HICs ($)** |
| --- | --- | --- | --- | --- | --- | --- | --- | --- | --- |
| **Tetracyclines** | **Noromycin** | 500 | 300 | 150000 | 94.99 | 633.27 | 1121.23 | 7949000 | 8,912,639,606 |
|  | **Liquamycin** | 500 | 200 | 100000 | 78.99 | 789.9 |  |  |  |
|  | **Duramycin** | 500 | 200 | 100000 | 41.99 | 419.9 |  |  |  |
|  | **Bio-Mycin** | 500 | 200 | 100000 | 66.49 | 664.9 |  |  |  |
|  | **OxyTet** | 500 | 100 | 50000 | 30.99 | 619.8 |  |  |  |
|  | **Terramycin** | 100 Tablets | 250 | 25000 | 89.99 | 3599.6 |  |  |  |
| **Amphenicols** | **NuFlor** | 500 | 300 | 150000 | 339.99 | 2266.6 | 2502.53 | 735000 | 1,839,362,000 |
|  | **Resflor** | 100 | 300 | 30000 | 91.99 | 3066.33 |  |  |  |
|  | **Norfenicol** | 500 | 300 | 150000 | 356.99 | 2379.933 |  |  |  |
|  | **LonCor** | 500 | 300 | 150000 | 344.59 | 2297.27 |  |  |  |
| **Penicillins** | **Penicillin** | 500 | 180 | 90000 | 42.95 | 477.22 | 534.17 | 3233000 | 1,726,960,833 |
|  | **CombiPen** | 250 | 180 | 45000 | 28.69 | 637.56 |  |  |  |
|  | **DuraPen** | 250 | 180 | 45000 | 27.49 | 610.89 |  |  |  |
|  | **ProPen** | 500 | 180 | 90000 | 36.99 | 411 |  |  |  |
| **Cephalosporins** | **Today** | 12x10ml | 20 | 2400 | 53.49 | 22287.5 | 18965.0 | 239000 | 4,532,633,672 |
|  | **Tomorrow** | 12x10ml | 30 | 3600 | 42.99 | 11941.67 |  |  |  |
|  | **Excede (Swine)** | 100 | 100 | 10000 | 135.95 | 13595 |  |  |  |
|  | **Excede (Cattle)** | 250 | 200 | 50000 | 568.99 | 11379.8 |  |  |  |
|  | **Excenel** | 50 | 250 | 12500 | 184.95 | 14796 |  |  |  |
|  | **Naxcel** | 1 grams | NA | 1000 | 39.79 | 39790 |  |  |  |
| **Sulfonamides** | **SulfaMed** | 250 | 400 | 100000 | 33.99 | 339.9 | 1221.29 | 1244000 | 1,519,281,961 |
|  | **SMZ TMP** | 473.176 | 40 | 18927.04 | 59.95 | 3167.43 |  |  |  |
|  | **Di-Methox** | 3800 | 126 | 478800 | 74.95 | 156.54 |  |  |  |
| **Macrolides** | **Tylan** | 500 | 200 | 100000 | 79.99 | 799.9 | 19352.79 | 1546000 | 29,919,416,530 |
|  | **Macrosyn** | 500 | 100 | 50000 | 868.99 | 17379.8 |  |  |  |
|  | **Zactran** | 250 | 150 | 37500 | 445.99 | 11893.07 |  |  |  |
|  | **Increxxa** | 250 | 100 | 25000 | 513.99 | 20559.6 |  |  |  |
|  | **Draxxin** | 250 | 100 | 25000 | 895.99 | 35839.6 |  |  |  |
|  | **Zuprevo** | 250 | 180 | 45000 | 1176.2 | 26137.78 |  |  |  |
|  | **Tulissin** | 500 | 100 | 50000 | 1142.99 | 22859.8 |  |  |  |
| **Amino-glycosides** | **Gentamicin** | 250 | 5 | 1250 | 16.99 | 13592 | 13592 | 639000 | 8,685,288,000 |
| **Quinolones** | **Baytril** | 250 | 100 | 25000 | 145.99 | 5839.6 | 5318.8 | 415000 | 2,207,302,000 |
|  | **Enroflox** | 250 | 100 | 25000 | 119.95 | 4798 |  |  |  |
| **Lincosamide** | **LincoMed** | 100 | 300 | 30000 | 22.99 | 766.33 | 766.333333 | 477000 | 365,541,000 |
| **Pleuro-mutuilins** | | NA | NA | NA | NA | NA | 292.477 | 230000 | 67,269,710 |
| **Polymyxins** | | NA | NA | NA | NA | NA | 2165.44  (estimated) | 172000 | 168,659,760 |
| **Other**  **Antibiotics** | | NA | NA | NA | NA | NA | 5984.73  (averaged) | 495000 | 3,485,577,445 |

**Table S6. Chinese pricing, aggregated sales data for LMICs, and estimated yearly revenue from antibiotics in LMICs.**

| **Class** | **Prices (RMB)** | **Average Price**  **(RMB)** | **Average Price ($)** | **Total Sales (LMICs)**  **(Tonnes)** | **Total Yearly Revenue LMICs ($)** |
| --- | --- | --- | --- | --- | --- |
| **Tetracyclines** | 176.6 | 326.30 | 48.95 | 25356000 | 1,241,049,420 |
|  | 476 |  |  |  |  |
| **Amphenicols** | 228.33 | 228.33 | 34.25 | 4317000 | 147,855,092 |
| **Beta-lactams^1^** | 273.33 | 453.54 | 68.03 | 9514000 | 647,246,934 |
|  | 120 |  |  |  |  |
|  | 168.33 |  |  |  |  |
|  | 593.33 |  |  |  |  |
|  | 540 |  |  |  |  |
|  | 1000 |  |  |  |  |
|  | 433.33 |  |  |  |  |
|  | 500 |  |  |  |  |
| **Cephalosporins** | 540 | 618.33 | 92.74 | 115000 | 10,666,249.9 |
|  | 1000 |  |  |  |  |
|  | 433.33 |  |  |  |  |
|  | 500 |  |  |  |  |
| **Sulphonamides** | 273.33 | 210.55 | 31.58 | 5824000 | 183,936,480 |
|  | 166.66 |  |  |  |  |
| **Macrolides** | 786.33 | 638.17 | 95.72 | 3498000 | 638,962,706 |
|  | 490 |  |  |  |  |
| **Aminoglycosides** | 630 | 413.33 | 62.00 | 2183264 | 216,876,087 |
|  | 196.667 |  |  |  |  |
| **Quinolones** | 176.66 | 176.66 | 26.50 | 1969000 | 52,176,531 |
| **Lincosamides** | 372.66 | 372.66 | 55.90 | 1320000 | 73,786,680 |
| **Pleuromutilins** | NA | NA | 10.89  (estimated) | 2124000 | 20,906,306.5 |
| **Polymixins** | 220 | 220.00 | 33 | 3786000 | 124,938,000 |
| **Other Antibiotics** | NA | NA | 50.78  (averaged) | 3113000 | 158,062,198 |

^1^Classified as penicillins in most surveillance reports but classified as beta-lactams in Chinese Ministry of Agriculture reports.

Estimation was required for Polymyxins (US/HICs), Pleuromutilin (China/LMICs) and for so called “Other” Antibiotics (HICs/LMICs). To calculate prices for Polymyxins and Pleuromutilins, a scaling factor was created describing the average multiplication factor between the prices of US and Chinese antibiotics for antibiotic classes which had pricing data for both countries. This scaling factor was 0.0337. This scaling factor was used to transform Chinese pricing for Polymyxins to estimate a US price of $980.58 per kg of active ingredient. US prices for pleuromutilins (Tiamulin) was used with the scaling factor to estimate a price of $9.82 per kg of active ingredient. Due to the non-specific nature of the “other” antibiotic class, an average of US and Chinese price data (which was not estimated) was used to create prices of $5984.73 and $50.78 per kg of active ingredient for each respective case study.

Prevalence data averaged across swine, cattle and chickens and pathogen (*Salmonella* spp. and *Campylobacter* spp.) were identified for each antibiotic class. Data from the 2021 NARMS report (US) and >2015 Chinese data from resistancebank.org were used to identify the prevalence of resistance for each antibiotic class and used to assign groupings for each antibiotic class [7, 8] (Table S7). Groupings correspond to the three groups used in the PED equations based on first, second and last line antibiotic groupings (eqn 1.1). These US and Chinese groupings were used as proxies for the HIC and LMIC case studies.

**Table S7. Prevalence data across pathogen, antibiotic class and livestock species, and PED groupings for China (LMICs) and US (HICs).**

| **Antibiotic Class** | **Chickens** | | **Swine** | | **Cattle** | | **Average** | **Group** |  |
| --- | --- | --- | --- | --- | --- | --- | --- | --- | --- |
|  | ***Salmonella*** | ***Campylobacter*** | ***Salmonella*** | ***Campylobacter*** | ***Salmonella*** | ***Campylobacter*** |  |  |  |
| **China - 2015 onwards** | | | | | | | | | |
| **Tetracyclines** | 52 | 82.6 | 59.3 | NA | 59.5 | NA | 63.35 | 1 |  |
| **Amphenicols** | 32 | 15.5 | 36.5 | NA | 85 | NA | 42.25 | 1 |  |
| **Penicillin** | 52.2 | 20.5 | 27.7 | NA | 25.33 | NA | 31.43 | 2 |  |
| **Cephalosporins** | 14.6 | 53.85 | 6.76 | NA | 14.33 | NA | 22.39 | 2 |  |
| **Sulphonamides** | 39.76 | NA | 40.54 | NA | 78 | NA | 52.77 | 1 |  |
| **Macrolides** | NA | 7.5 | NA | NA | NA | NA | 7.5 | 3 |  |
| **Aminoglycosides** | 21.6 | 32.2 | 17.62 | NA | 21 | NA | 23.11 | 2 |  |
| **Quinolones** | 34 | 96.6 | 12.9 | NA | 16 | NA | 39.88 | 2 |  |
| **Lincosamides** | NA | 26 | NA | NA | NA | NA | 26 | 2 |  |
| **Pleuromutilins** | NA | NA | NA | NA | NA | NA | NA | 2  (assumed) |  |
| **Polymixins** | 5.5 | NA | 6 | NA | NA | NA | 5.75 | 3 |  |
| **Other Antibiotics** | NA | NA | NA | NA | NA | NA | NA | 2  (assumed) |  |
| **United States – 2021** | | | | | | | | | |
| **Tetracyclines** | 6.7 | 57.9 | 57.6 | 42.7 | 30.4 | 72.7 | 44.67 | 1 |  |
| **Amphenicols** | 3.3 | NA | 15 | NA | 2.4 | NA | 6.9 | 3 |  |
| **Penicillin** | 3 | NA | 14.2 | NA | 10.2 | NA | 9.13 | 2 |  |
| **Cephalosporins** | 9.8 | NA | 6.7 | NA | 1.8 | NA | 6.1 | 3 |  |
| **Sulphonamides** | 5 | NA | 39.3 | NA | 15.1 | NA | 19.8 | 1 |  |
| **Macrolides** | 0.9 | 0.4 | 0 | 0.8 | 1.3 | 17.6 | 3.5 | 3 |  |
| **Aminoglycosides** | 0.7 | 0.6 | 21.9 | 0.5 | 4.7 | 2.7 | 5.18 | 3 |  |
| **Quinolones** | 5.3 | 34.3 | 0 | 17.9 | 5.3 | 2.7 | 10.92 | 2 |  |
| **Lincosamides** | NA | 0.4 | NA | 0.8 | NA | 17.1 | 6.1 | 3 |  |
| **Pleuromutilins** | NA | NA | NA | NA | NA | NA | NA | 2 (assumed) |  |
| **Polymyxins** | NA | NA | NA | NA | NA | NA | NA | 3 (Assumed) |  |
| **Other Antibiotics** | NA | NA | NA | NA | NA | NA | NA | 2  (assumed) |  |

For certain antibiotic classes: Pleuromutilin, Polymyxins (US) and so called “other” antibiotics, several simplifying assumptions were required to assign PED groupings. For Pleuromutilins, literature suggests that resistance can range from 0-40% based on the clinical MIC breakpoint (of which there is no approved value for pleuromutilins) [9]. Pleuromutilins are a key treatment for swine dysentery, with widescale resistance predicted to lead to considerable consequences for swine production. In comparison, there is only a single approved pleuromutilin treatment in humans (retapamulin) used for topical usage, suggested limited health consequences. We therefore assigned a PED grouping value of 2. On the other hand, polymyxins include colistin, a last line drug in humans and widescale application for food animal treatment in agricultural settings, we therefore assigned a PED grouping value of 3 for polymyxins [10, 11]. For “other” antibiotics, we assigned an intermediate PED grouping of 2.

*Alternative Taxation Rates*

Revenue analyses were also conducted using alternative values for the taxation rate (25% and 75%) (Figure S30-31)

*
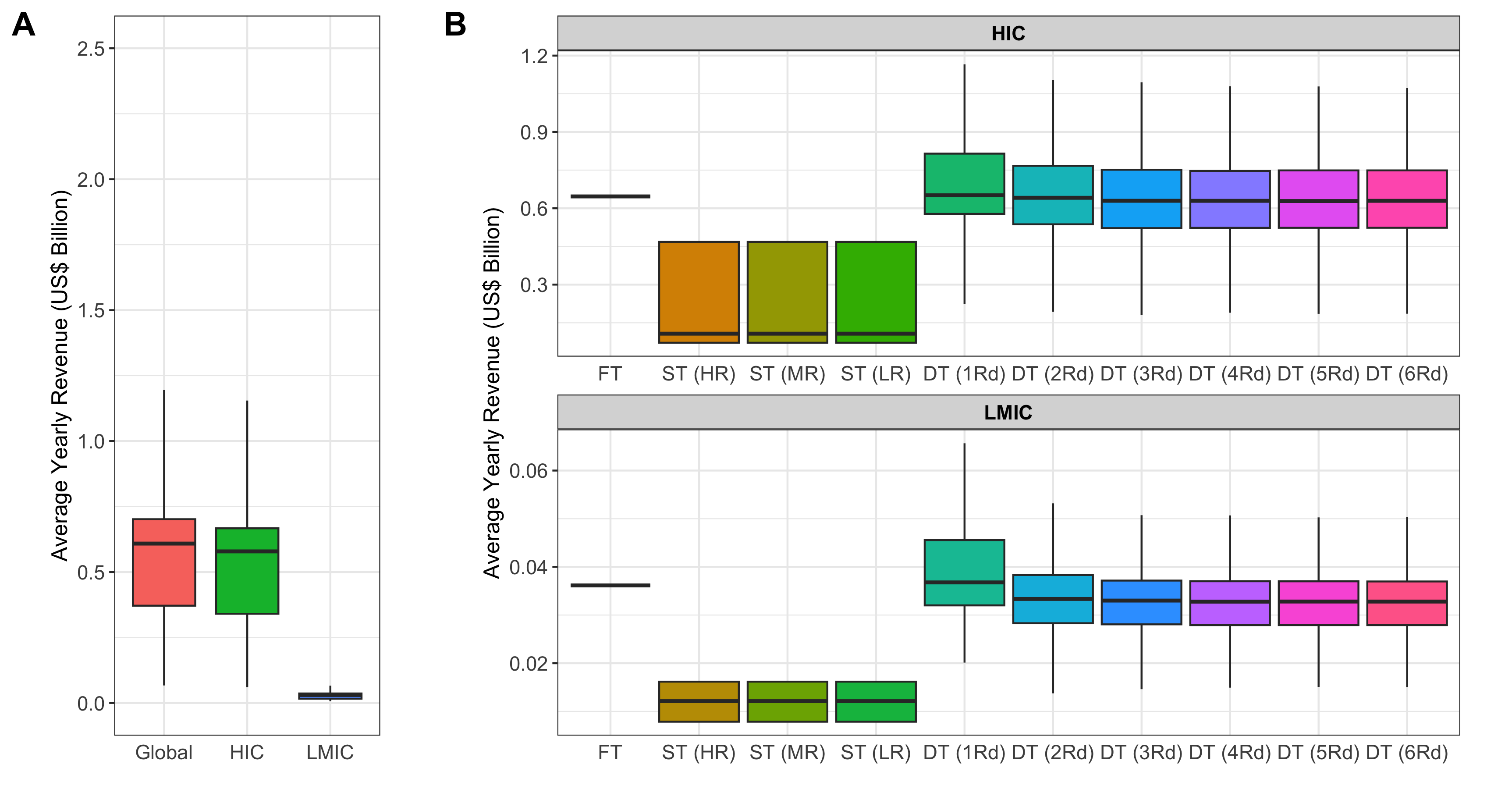
*

**Figure S30. Projected revenue from taxation strategies for High-Income Countries (HICs), Lower-Middle Income Countries (LMICs) using a 25% taxation rate. A) Aggregated median income for all countries, HICs and LMICs across all explored taxation strategies. B) Estimated revenue for each taxation strategy.** FT = Flat Tax, ST = Single Tax, DT = Differential Tax, HR = High Resistance, MR = Medium Resistance, LR = Low Resistance.

*
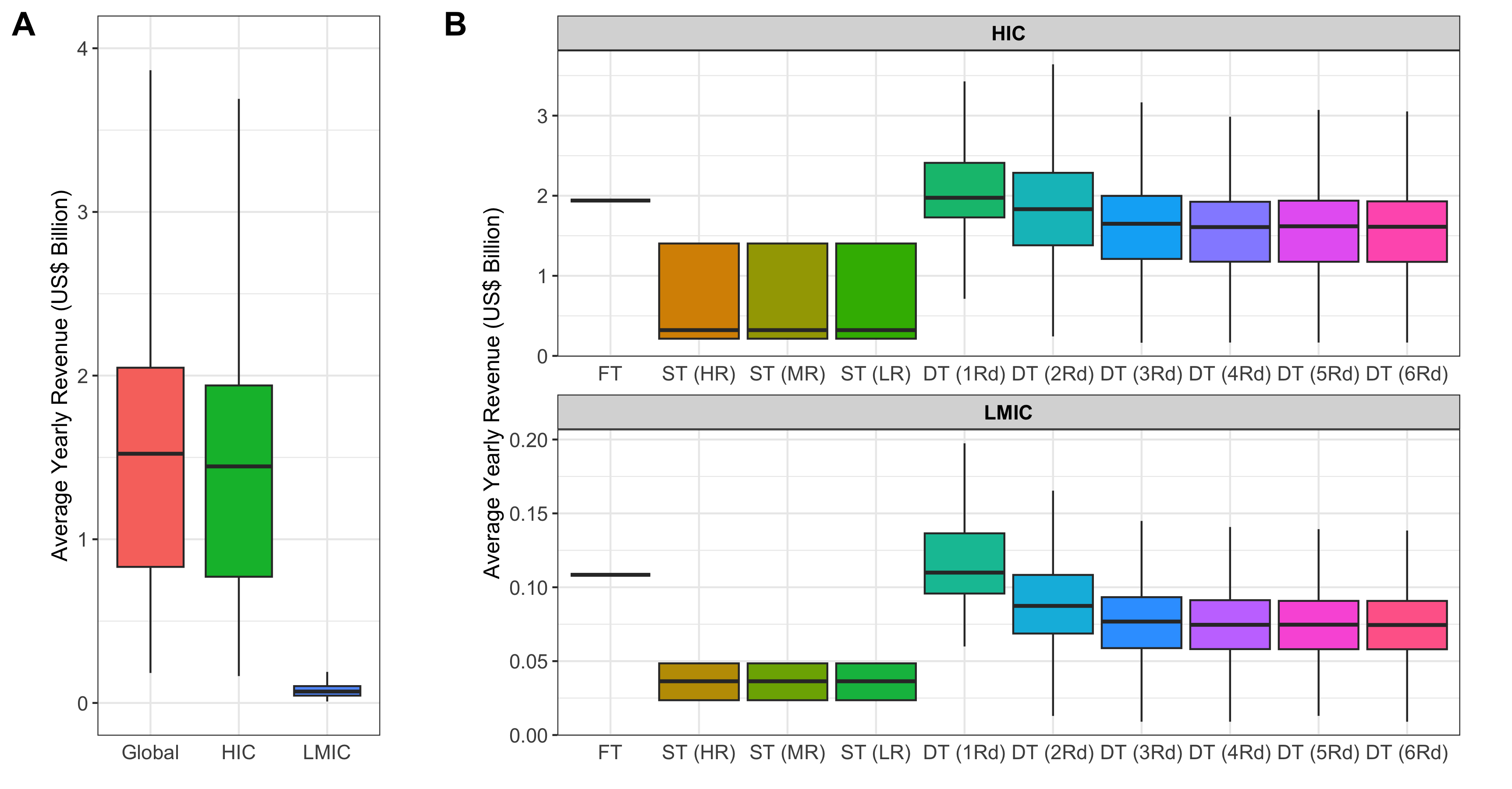
*

**Figure S31. Projected revenue from taxation strategies for High-Income Countries (HICs), Lower-Middle Income Countries (LMICs) using a 75% taxation rate. A) Aggregated median income for all countries, HICs and LMICs across all explored taxation strategies. B) Estimated revenue for each taxation strategy.** FT = Flat Tax, ST = Single Tax, DT = Differential Tax, HR = High Resistance, MR = Medium Resistance, LR = Low Resistance.

*References*

1. A Minter, Retkute R. Approximate Bayesian Computation for infectious disease modelling. *Epidemics*. (2019). 29:100368.

2. T Toni, Welch D, Strelkowa N, Ipsen A, Stumpf MP. Approximate Bayesian computation scheme for parameter inference and model selection in dynamical systems. *Journal of the Royal Society Interface*. (2009). 6(31):187-202.

3. JW White, Rassweiler A, Samhouri JF, Stier AC, White C. Ecologists should not use statistical significance tests to interpret simulation model results. *Oikos*. (2014). 123(4):385-8.

4. SCSP Global. *Farm Animal Antibiotics Market 2022: Animal Health Market Analysis*. Frauenfeld, Switzerland: Stonehaven Consulting. (2022). Available.

5. The World Bank. *World Bank Country and Lending Groups.* Washington, D.C., United States: The World Bank. (2021) [cited 2023 06/01/2023]. Available from: <https://datahelpdesk.worldbank.org/knowledgebase/articles/906519-world-bank-country-and-lending-groups>.

6. R Mulchandani, Wang Y, Gilbert M, Van Boeckel TP. Global trends in antimicrobial use in food-producing animals: 2020 to 2030. *PLOS Global Public Health*. (2023). 3(2):e0001305.

7. Food & Drug Administration. *NARMS Now.* Rockville, MD: U.S. Department of Health and Human Services. (2023) [Available from: <https://www.fda.gov/animal-veterinary/national-antimicrobial-resistance-monitoring-system/narms-now-integrated-data>.

8. NG Criscuolo, Pires J, Zhao C, Van Boeckel TP. resistancebank. org, an open-access repository for surveys of antimicrobial resistance in animals. *Scientific Data*. (2021). 8(1):1-10.

9. E van Duijkeren, Greko C, Pringle M, Baptiste KE, Catry B, Jukes H, et al. Pleuromutilins: use in food-producing animals in the European Union, development of resistance and impact on human and animal health. *J Antimicrob Chemother*. (2014). 69(8):2022-31.

10. RL Nation, Li J. Colistin in the 21st century. *Curr Opin Infect Dis*. (2009). 22(6):535-43.

11. H Kumar, Chen BH, Kuca K, Nepovimova E, Kaushal A, Nagraik R, et al. Understanding of Colistin Usage in Food Animals and Available Detection Techniques: A Review. *Animals (Basel)*. (2020). 10(10).
